# Synthesis of Thiazoloindole α-Amino Acids: Chromophores Amenable to One- and Two-Photon Induced Fluorescence

**DOI:** 10.1021/acs.orglett.3c03851

**Published:** 2023-12-06

**Authors:** Amy C. Dodds, Henry G. Sansom, Steven W. Magennis, Andrew Sutherland

**Affiliations:** School of Chemistry, The Joseph Black Building, University of Glasgow, Glasgow G12 8QQ, United Kingdom

## Abstract

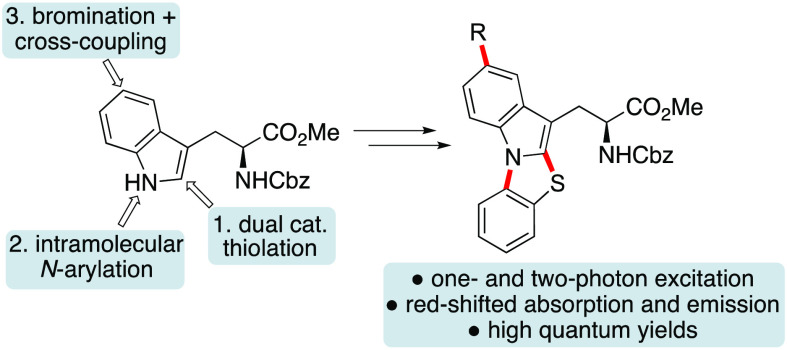

Thiazoloindole α-amino acids have been synthesized in four steps from tryptophan using a dual-catalytic thiolation reaction and a copper-mediated intramolecular N-arylation process. Late-stage diversification of the thiazoloindole core with electron-deficient aryl substituents produced chromophores that on one-photon excitation displayed blue-green emission, mega-Stokes shifts, and high quantum yields. The thiazoloindole amino acids could also be excited via two-photon absorption in the near-infrared, demonstrating their potential for biomedical imaging applications.

Fluorescence spectroscopy is a major technique for studying and understanding biological processes.^1^ Its high detection sensitivity has enabled the real-time monitoring of dynamic molecular processes *in vitro* and *in vivo* over a wide range of time scales and down to the single-molecule level.^2,3^ Continuing the evolution of fluorescence spectroscopy as a technique to study biological events requires the development of novel functional probes.^2,3^ In chemical biology, a typical strategy is the labeling of peptides and proteins with an extrinsic chromophore.^4^ However, the size of these fluorescent labels generally necessitates the use of a chemical spacer to prevent the disruption of the protein structure. Furthermore, the incorporation of these fluorophores is generally restricted to the N- or C-terminus of the protein. An alternative approach is the use of fluorescent amino acids that can be incorporated at predetermined positions of a protein, resulting in site specific imaging. Although fluorescent proteinogenic α-amino acids such as l-tryptophan (**1**) (Figure 1a) have been used for imaging, their sup-optimal properties have led to the development of brighter unnatural analogues.^5^ A major approach in achieving this aim has involved extending the conjugation of the indole side chain of l-tryptophan (**1**).^6,7^ This has led to unnatural amino acids with improved fluorescent properties, such as l-4-cyanotryptophan (**2**),^8^ which has been used to study peptide–membrane interactions, or BODIPY conjugate **3**,^9^ that has been used to visualize fungal infections in human tissue. Other strategies have investigated heterocyclic analogues of the indole unit, such as benzotriazoles (**4**),^10^ azaindoles, and pyrrolo-isoquinoline systems, some of which have been used to monitor protein conformational changes.^7b,7c^

**Figure 1:**
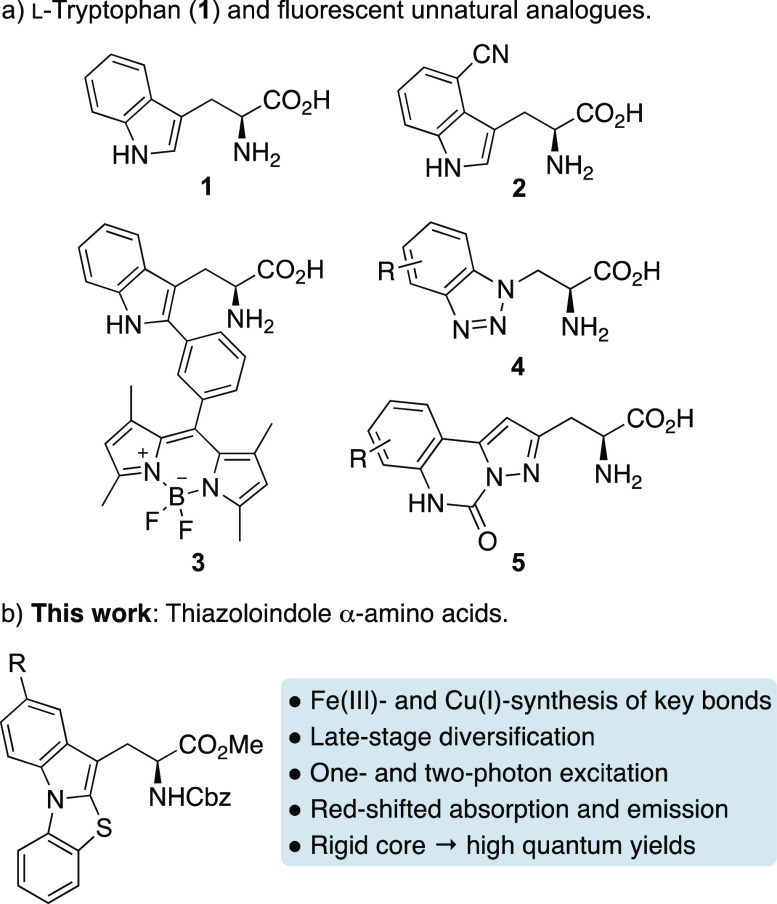
l-Tryptophan and fluorescent unnatural amino acids.

Although fluorescent unnatural α-amino acids with improved photophysical properties have been used for imaging experiments, a limitation is that ultraviolet (UV) light is often required for excitation. This is an issue, as UV light has limited penetration depth and can cause photobleaching of fluorophores and photodamage to biological tissue. In addition, the absorbance of UV light can result in the excitation of autofluorescence from intrinsic biological fluorophores, resulting in interference with the fluorescence signal from the probe. One approach to overcome these issues is the use of two-photon excitation, which involves the simultaneous absorption of two longer wavelength photons typically in the near-infrared.^11^ Thus, a one-photon chromophore that is excited around 350 nm can be excited by two photons at 700 nm, potentially reducing photobleaching, increasing penetration depth, and producing images with 3D spatial resolution. Although two-photon excited fluorescence has been used on a wide range of organic fluorophores for biomedical imaging,^11^ examples of fluorescent unnatural α-amino acids are relatively rare. The Mély and Vendrell groups described peptides containing either 3-hydroxflavone or tryptophan-BODIPY α-amino acids (e.g., **3**, Figure 1a), respectively, which were measured by two-photon microscopy for biological imaging applications.^9,12^ More recently, we reported two-photon-induced fluorescence by near-IR excitation of an α-amino acid bearing a pyrazoloquinazoline side chain (**5**).^13^ To benefit from the advantages of two-photon microscopy for novel bioimaging applications, further examples are required of two-photon excitable α-amino acids. Herein, we report a new class of unnatural α-amino acids bearing a thiazoloindole side chain (Figure 1b), in which a dual-catalytic thiolation of tryptophan followed by a copper-mediated N-arylation reaction are used as the key steps. We also describe the photophysical properties of these amino acids and show their potential for imaging applications via two-photon excitation.

Recently, reported methods for the syntheses of [3,2-*a*]thiazoloindoles have typically involved the preparation of N- or C2-alkynyl-substituted indoles, followed by base-mediated cyclization.^14^ Instead, we proposed an alternative two-step approach, utilizing a regioselective C2-thioarylation of the tryptophan indole ring via iron(III) and diphenyl selenide dual-catalyzed activation of *N*-thiosuccinimide **7**,^15−17^ followed by a copper-mediated N-arylation reaction between the *ortho*-bromide substituent and the adjacent indole amine (Scheme 1). It was then proposed that the [3,2-*a*]thiazoloindole ring system could be extended, allowing substituent-based tuning of the photophysical properties, by regioselective bromination, followed by a Suzuki–Miyaura cross-coupling reaction.

**Scheme 1:**
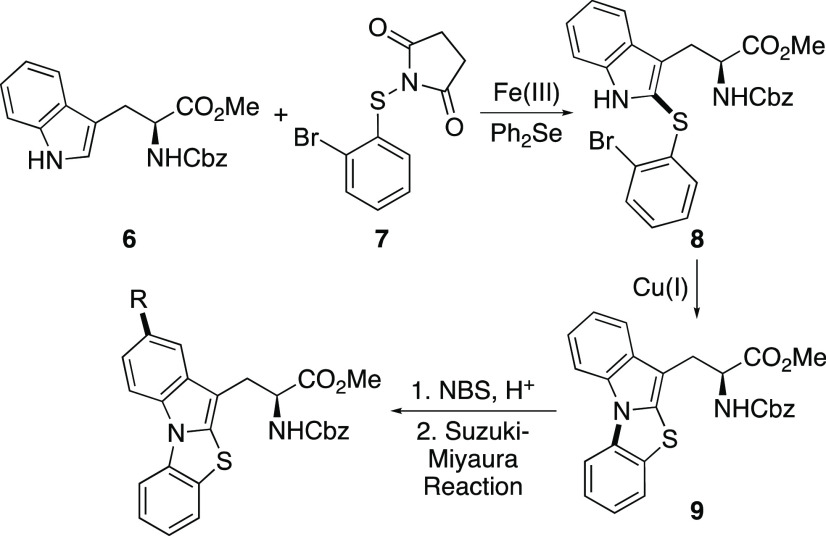
Proposed Synthesis of Thiazoloindole α-Amino Acids

The four-step synthesis of protected thiazoloindole amino acids is shown in Scheme 2. We have previously shown that iron-catalyzed thioarylation using *N*-(2-bromophenylthio)succinimide (**7**) can be accelerated by Lewis base catalysis.^17b^ This transformation proceeds by Fe^3+^ activation of the succinimide reagent, followed by reaction with a Lewis base, such as diphenyl selenide to give a cationic adduct.^18^ This more activated cationic species then rapidly reacts with the arene to yield the thioarylated product.^19^ Thioarylation of *N*-Cbz-tryptophan α-methyl ester (**6**) using succinimide **7** and a combination of iron(III) triflimide (10 mol %), prepared in situ from iron(III) chloride and the ionic liquid, [BMIM]NTf_2_, and diphenyl selenide (10 mol %), was found to give clean C2-thioarylation of the indole ring. At 90 °C and a reaction time of 18 h, adduct **8** was formed as the sole product in an 88% yield. The reaction was amenable to scale-up and was routinely performed on ∼1 g quantities. Cyclization to form the [3,2-*a*]thiazoloindole ring was then achieved using an Ullmann-type reaction.^20^ The use of commercially available copper(I) thiophene-2-carboxylate (CuTC) under neutral conditions gave thiazoloindole **9** in an 81% yield. The conjugation of the thiazoloindole core was then extended using a two-step approach. Regioselective bromination of **9** using NBS and catalytic HBr gave **10** in a 79% yield. A range of electron-rich and electron-deficient arene substituents were then incorporated via a Suzuki–Miyaura reaction.^21^ The use of the Buchwald XPhos Pd G2 catalyst allowed efficient cross-coupling reactions (74–88% yields), under relatively mild conditions and fast reaction times.^22,23^ Overall, this four-step route allowed efficient access to a small library of novel α-amino acids with the late-stage incorporation of diversity.

**Scheme 2:**
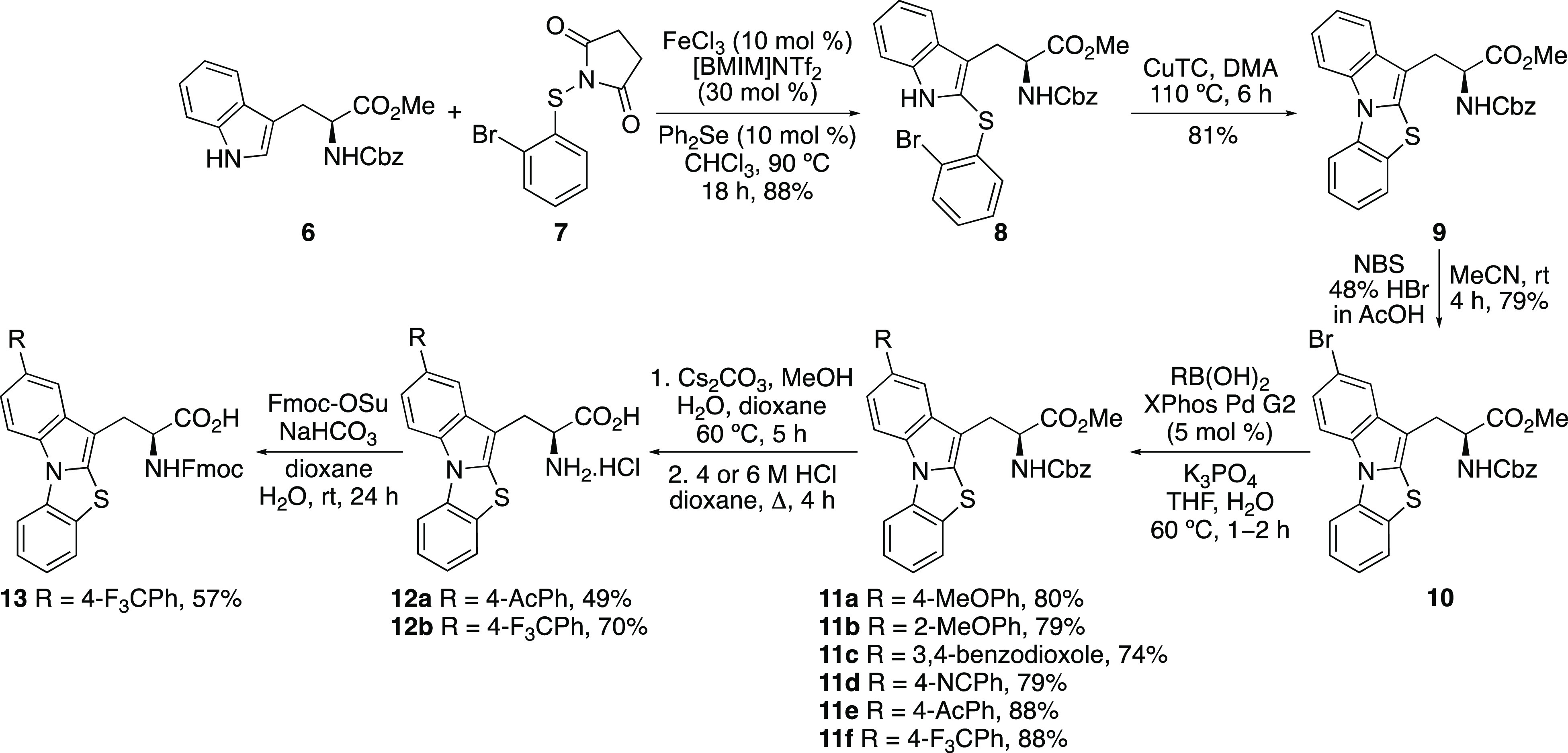
Synthesis of Thiazoloindole α-Amino Acids

The optical properties of protected α-amino acids **11a**–**11f** were then measured (Table 1).^24^ Although amino acids **11a**–**11c** with electron-rich aryl substituents showed red-shifted absorption and emission compared to l-tryptophan (**1**),^25^ the most interesting properties were found for amino acids **11d**–**11f**, bearing electron-deficient substituents. These were found to have significantly more red-shifted absorption and emission and much higher quantum yields, resulting in a bright blue-green fluorescence (Figure 2). Amino acid **11d** with a 4-cyanophenyl side chain was found to have an emission maximum at ∼500 nm, a quantum yield of 0.78, and a brightness of 16 × 10^3^ cm^–1^ M^–1^, while amino acid **11f** with a 4-trifluoromethylphenyl side chain possessed the highest quantum yield of 0.92, resulting in the strongest brightness (17 × 10^3^ cm^–1^ M^–1^). Although amino acid **11e** with a 4-acetylphenyl side chain had a lower quantum yield of 0.64, it exhibited a mega-Stokes shift (8112 cm^–1^) and the most red-shifted emission maximum at 541 nm. Thus, the combination of the electron-rich thiazoloindole core with the electron-deficient aryl substituents generated bright, charge-transfer-based fluorophores.

**Figure 2:**
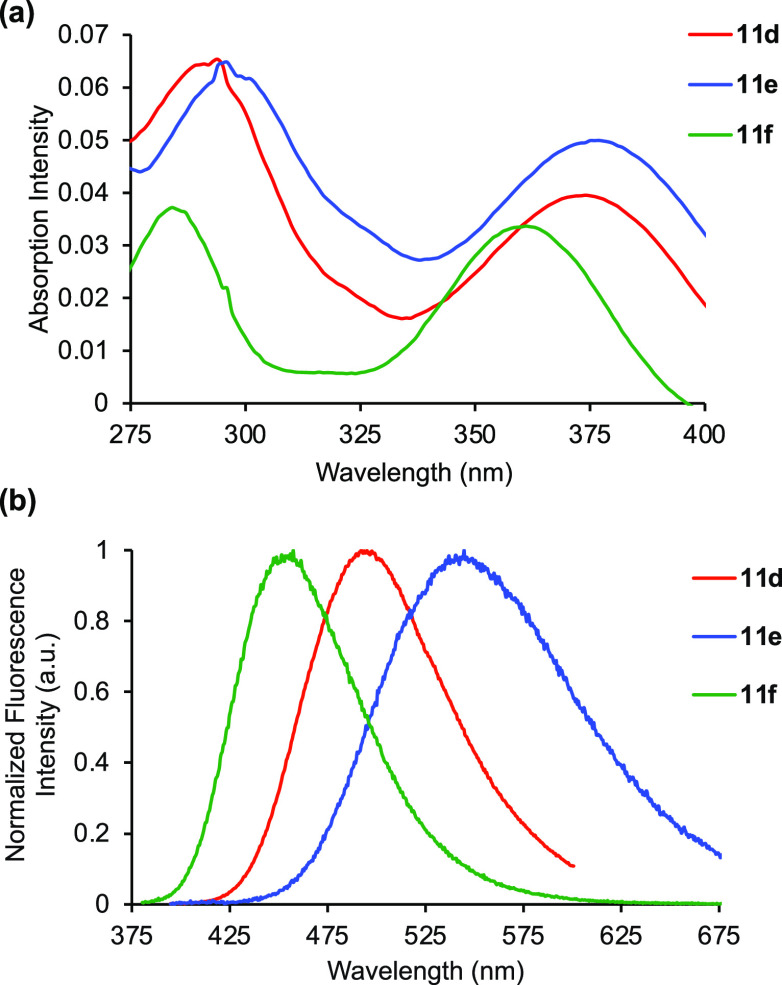
(a) Absorption spectra of **11d**–**11f**, recorded at 2 μM in DMSO. (b) Emission spectra of **11d**–**11f**, recorded at 2 μM in DMSO.

**Table 1:** Photophysical Data of α-Amino Acids^26^

amino acid	λ_Abs_ (nm)^a^	ε (×10^4^ cm^–1^ M^–1^)	λ_Em_ (nm)^a^	Φ_F_^b^	brightness (×10^3^ cm^–1^ M^–1^)
**1**	279	0.56	348	0.20	1.1
**11a**	348	1.91	392	0.049	0.93
**11b**	343	1.85	405	0.084	1.6
**11c**	350	2.11	408	0.14	2.9
**11d**	373	2.04	497	0.78	16
**11e**	376	2.08	541	0.64	13
**11f**	359	1.89	453	0.92	17
**12a**	378	1.48	545	0.46	6.7
**12b**	362	1.61	461	0.73	12

a Spectra were recorded at 2 μM in DMSO.

b Quantum yields (Φ_F_) were determined in DMSO using anthracene and l-tryptophan as standards.

Based on these properties, the thiazoloindole α-amino acids were further investigated to discover whether these would be responsive to two-photon excitation for potential biological applications. Amino acids **11e** and **11f** were selected for further analysis.^27^ Initially, these were deprotected to the parent amino acids **12a** and **12b** (Scheme 2). Ester hydrolysis was performed using cesium carbonate, and this was followed by removal of the Cbz-protecting group under acidic conditions. Following recrystallization, this gave the hydrochloride salts **12a** and **12b** in 49% and 79% yields, respectively, over the two steps. The photophysical properties of amino acids **12a** and **12b** were then measured. Although most properties were retained by the deprotected amino acids, a decrease in the quantum yield was observed (Table 1). However, the values for both were still relatively high, particularly amino acid **12b**, with a quantum yield of 0.73 and a brightness of ∼12 × 10^3^ cm^–1^ M^–1^.^28^ Fluorescence lifetimes following one-photon excitation were also measured and found to be 4.04 ns for **12a** and 3.54 ns for **12b**. Amino acids **12a** and **12b** were then subjected to two-photon excitation using a broadband Ti:sapphire laser with a central wavelength of 800 nm.^24^ As shown in Figure 3, the resulting emission spectra were found to have the same profile as that obtained from one-photon excitation at 400 nm. A log–log plot of fluorescence intensity versus power generated a slope of 1.92 for both amino acids, thereby confirming two-photon excitation.^24,29^ The two-photon cross sections of **12a** and **12b** were measured at excitation wavelengths of 700 and 800 nm. The cross sections of **12a** were 29 ± 4 GM at 700 nm and 31 ± 5 GM at 800 nm, while, for **12b**, these were 21 ± 3 and 1.2 ± 0.2 GM at 700 and 800 nm, respectively. Combining these values with the quantum yields results in two-photon brightness (σ_2_Φ_F_) of 14 ± 2 GM for **12a** at 800 nm and 15 ± 2 GM for **12b** at 700 nm, which are comparable to those of fluorescent nucleobase analogues that have been detected at the single-molecule level.^29a,29b^ These results demonstrate that thiazoloindole amino acids such as **12a** and **12b** can undergo two-photon absorption using near-IR excitation, avoiding UV excitation and the potential associated issues of photodamage. In combination with the relatively long fluorescence lifetimes, these amino acids show potential for biological imaging.

**Figure 3:**
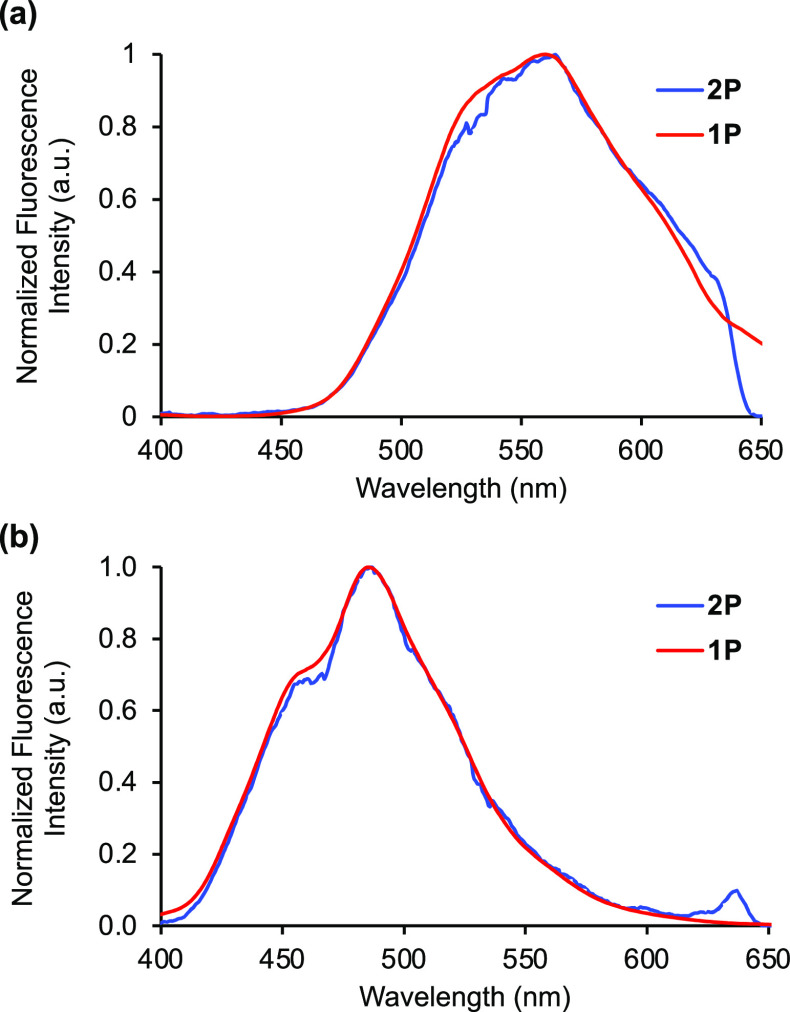
(a) Emission spectrum of **12a** after one- and two-photon absorption (5.7 μM in DMSO). (b) Emission spectrum of **12b** after one- and two-photon absorption (5.5 μM in DMSO).

In summary, the synthesis and photophysical properties of a novel class of α-amino acid is reported. The thiazoloindole side chain of these amino acids was prepared using a dual-catalytic, regioselective C2-thiolation of tryptophan, followed by a copper-mediated N-arylation reaction. Late-stage diversification via bromination and Suzuki–Miyaura cross-coupling reactions allowed for the preparation of a small library of analogues. Thiazoloindole amino acids with electron-deficient aryl groups were found to have red-shifted absorption and emission spectra relative to tryptophan, with enhanced quantum yields and bright charge-transfer-based fluorescence. Significantly, two of the amino acids were found to be amenable to two-photon absorption by near-IR excitation, thereby avoiding excitation with UV light. Amino acid **12b** was readily converted to Fmoc-derivative **13** (Scheme 2), and thus, future work will exploit these amino acids for solid phase peptide synthesis and subsequent bioimaging applications.

## Data Availability

The data underlying this study are available in the published article and its Supporting Information.

## Supporting Information Available

Experimental procedures, characterization data, photophysical data, and NMR spectra of all compounds (PDF)
