# Supplementary material for: Synthesis of Thiazoloindole α-Amino Acids: Chromophores Amenable to One- and Two-Photon Induced Fluorescence
Source: Org Lett. 2023 Dec 6;25(49):8942–6. doi: 10.1021/acs.orglett.3c03851 (PMC10729019; doi:10.1021/acs.orglett.3c03851)

Supporting Information for:

Synthesis of Thiazoloindole α-Amino Acids: Chromophores Amenable to One- and Two-Photon Induced Fluorescence

*Amy C. Dodds, Henry G. Sansom, Steven W. Magennis and Andrew Sutherland**

School of Chemistry, The Joseph Black Building, University Avenue, University of Glasgow, Glasgow, G12 8QQ, United Kingdom.

Table of Contents

1. Proposed Mechanism for the Dual-Catalytic Thioarylation Reaction S2
2. General Experimental S2–S3
3. Experimental Procedures and Spectroscopic Data for all Compounds S3–S14
4. Photophysical Data for α-Amino Acids S15–S23
5. Two-Photon Calculations, Experimental Details and Spectra S24–S26
6. Time Correlated Single-Photon Counting (TCSPC) S26–S27
7. References S28
8. ^{1}H and ^{13}C NMR Spectra for all Novel Compounds S29–S53

1. Proposed Mechanism for the Dual-Catalytic Thioarylation Reaction.

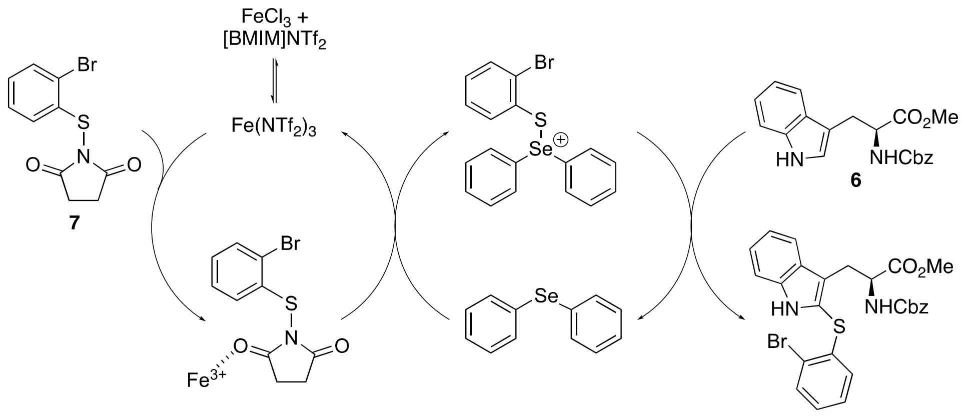

2. General Experimental: All reagents and starting materials were obtained from commercial sources and used as received. *N*-(2-Bromophenylthio)succinimide (**7**) was prepared as previously described.[1] Dry solvents were purified using a PureSolv 500 MD solvent purification system. All reactions were performed open to air unless otherwise mentioned. All reactions performed at elevated temperatures were heated using an oil bath. Brine refers to a saturated aqueous solution of sodium chloride. Flash column chromatography was carried out using Merck Millipore matrix silicagel 60 (40–63 μM). Merck aluminium-backed plates pre-coated with silica gel 60 (UV_{254}) were used for thin-layer chromatography and visualized with a UV lamp. ^{1}H NMR and ^{13}C NMR spectra were recorded on a Bruker DPX 400 MHz spectrometer, with chemical shift values reported in ppm relative to tetramethylsilane (δ_H 0.00 and δ_c 0.00), residual chloroform (δ_H 7.26) or dimethylsulfoxide (δ_H 2.50) as standard. For ^{13}C NMR the chemical shifts are reported relative to the central resonance of $CDCl_3$ (δ_c 77.2) or DMSO-d_6 (δ_c 39.5) as standard. Carbon assignments are based on two-dimensional HMBC and DEPT experiments. Mass spectra were obtained using a Bruker Microtof-q or Agilent 6125B. Infrared spectra were obtained neat using a Shimadzu IR Prestige-21 spectrometer or Shimadzu 8400S spectrometer; wavenumbers are indicated in cm^{-1}. Melting points were determined on either a Reichert platform melting point apparatus or Stuart Scientific melting point apparatus. Optical rotations were determined as solutions irradiating with the sodium D line (λ = 589 nm) using an Autopol V polarimeter. $[\alpha]_D$ values are given in units 10^{-1} deg cm^2 g^{-1}. Both UV-Vis spectra and fluorescence spectra were recorded on a Horiba Duetta Fluorescence and Absorbance spectrometer. Absorbance spectra were recorded with an integration time of 0.05 s, and a band pass of 5 nm. Uncorrected fluorescence spectra for the thiazoloindole α-amino acids were recorded with excitation and emission band pass of 5 nm, an integration time of 0.05 s, and with detector accumulations set to 1. Respective standard samples were recorded with the same parameters. Quantum yields were determined using a comparative method against two standards. Anthracene (Φ = 0.27, in ethanol) and L-tryptophan (Φ = 0.14 in water) were used as standard references.[2] The integrated fluorescence

intensity of each compound was determined from the emission spectra given. Measurements were performed at five different concentrations. Concentrations were chosen to ensure the absorption value was below 0.1 to avoid re-absorption effects. Integrated fluorescence intensity was plotted as a function of the measured absorbance and a linear fit was calculated. The resultant gradient was then used to calculate the quantum yield, using the equation below:

$$\phi_x = \phi_{ST}(\frac{Grad_{ST}}{Grad_X})(\frac{\eta_x^2}{\eta_{ST}^2})$$

Subscript ST signifies the quantities associated with the quantum yield standard. Subscript X signifies the quantities associated with the novel compound. $Grad_X$ is the determined gradient associated with the novel compound. $Grad_{ST}$ is the determined gradient associated with quantum yield standard. η is the refractive index of the solvent used in the fluorescence measurements. η = 1.333 for water, 1.361 for ethanol, 1.331 for methanol and 1.4772 for DMSO.

3. Experimental Procedures and Spectroscopic Data for all Compounds

_N_-Benzyloxycarbonyl-L-tryptophan methyl ester (6)[3]

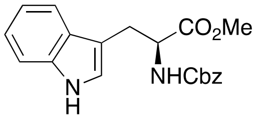

To a solution of L-tryptophan methyl ester hydrochloride (2.00 g, 7.85 mmol) and sodium hydrogencarbonate (3.30 g, 39.3 mmol) in water (20 mL) and dichloromethane (60 mL) at 0 °C, was added benzyl chloroformate (1.23 mL, 8.64 mmol) dropwise. The reaction mixture was warmed to room temperature and left to stir for 2 h. The two layers were separated and the aqueous layer was further extracted with dichloromethane (2 × 30 mL). The combined organic layers were washed with brine (50 mL), dried over $MgSO_4$, filtered and concentrated *in vacuo*. Purification by flash column chromatography (hexane/ethyl acetate, 2:3) gave *N*-benzyloxycarbonyl-L-tryptophan methyl ester (**6**) as a colorless oil (2.63 g, 95%). $[\alpha]_D^{23}$ +49.0 (*c* 0.1, $CHCl_3$), lit.[3] $[\alpha]_D^{20}$ +47.6 (*c* 0.2, $CHCl_3$); ^{1}H NMR (400 MHz, $CDCl_3$) δ 8.13 (br s, 1H), 7.53 (dd, 1H, *J* = 7.2, 1.3 Hz), 7.42–7.26 (m, 6H), 7.19 (ddd, 1H, *J* = 8.1, 7.0, 1.3 Hz), 7.10 (ddd, 1H, *J* = 8.1, 7.2, 1.1 Hz), 6.94 (d, 1H, *J* = 2.5 Hz), 5.34 (d, 1H, *J* = 8.3 Hz), 5.14 (d, 1H, *J* = 12.3 Hz), 5.09 (d, 1H, *J* = 12.3 Hz), 4.73 (dt, 1H, *J* = 8.3, 5.5 Hz), 3.68 (s, 3H), 3.32 (d, 2H, *J* = 5.5 Hz); $^{13}C\{^1H\}$ NMR (101 MHz, $CDCl_3$) δ 172.5 (C), 155.9 (C), 136.4 (C), 136.2 (C), 128.6 (2 × CH), 128.3 (2 × CH), 128.2 (CH), 127.7 (C), 122.9 (CH), 122.4 (CH), 119.8 (CH), 118.7 (CH), 111.4 (CH), 110.0 (C), 67.0 (CH_2), 54.6 (CH), 52.5 (CH_3), 28.1 (CH_2); MS (ESI) *m/z* 375 (M + Na^+, 100).

N-[(Benzyloxycarbonyl)amino]-[2'-(2"-bromophenylthio)]-L-tryptophan methyl ester (8)

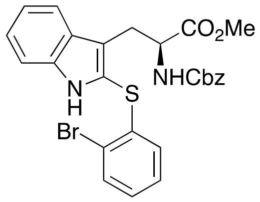

Iron(III) trichloride (0.0529 g, 0.326 mmol) was dissolved in [BMIM]NTf_2 (0.285 mL, 0.978 mmol) and stirred for 0.5 h at room temperature before being added to a solution of *N*-(2-bromophenylthio)succinimide (**7**) (1.21 g, 3.91 mmol) in chloroform (10 mL). *N*-[(Benzyloxycarbonyl)amino]-L-tryptophan methyl ester (**6**) (1.15 g, 3.26 mmol) and diphenyl selenide (0.0563 mL, 0.326 mmol) were then added and the mixture was left to stir at 90 °C for 18 h. The reaction mixture was concentrated *in vacuo*. Purification by flash column chromatography (hexane/ethyl acetate, 4:1) gave *N*-[(benzyloxycarbonyl)amino]-[2'-(2"-bromophenylthio)]-L-tryptophan methyl ester (**8**) (1.54 g, 88%) as a white solid. Mp 82−84 °C; IR (neat) 3295, 2951, 1698, 1506, 1444, 1343, 1209, 1018, 741 cm^{-1}; $[\alpha]_D^{24}$ +33.5 (*c* 0.1, $CHCl_3$); ^1H NMR (400 MHz, $CDCl_3$) δ 8.17 (br s, 1H), 7.63 (br d, 1H, *J* = 8.2 Hz), 7.48 (dd, 1H, *J* = 7.8, 1.5 Hz), 7.38–7.27 (m, 7H), 7.15 (ddd, 1H, *J* = 8.2, 6.9, 1.2 Hz), 7.01 (ddd, 1H, *J* = 7.8, 7.5, 1.5 Hz), 6.93 (ddd, 1H, *J* = 7.8, 7.5, 1.7 Hz), 6.50 (dd, 1H, *J* = 7.8, 1.7 Hz), 5.30 (d, 1H, *J* = 8.7 Hz), 5.05 (s, 2H), 4.72 (ddd, 1H, *J* = 8.7, 6.4, 5.7 Hz), 3.69 (s, 3H), 3.45 (dd, 1H, *J* = 14.4, 5.7 Hz), 3.33 (dd, 1H, *J* = 14.4, 6.4 Hz); $^{13}C\{^1H\}$ NMR (101 MHz, $CDCl_3$) δ 172.2 (C), 155.8 (C), 138.3 (C), 137.4 (C), 136.4 (C), 133.0 (CH), 128.6 (2 × CH), 128.24 (CH), 128.23 (2 × CH), 128.20 (CH), 128.0 (C), 127.1 (CH), 127.0 (CH), 124.3 (CH), 122.4 (C), 120.6 (CH), 120.2 (C), 119.6 (CH), 119.0 (C), 111.4 (CH), 67.0 (CH_2), 54.5 (CH), 52.7 (CH_3), 27.9 (CH_2); MS (ESI) *m/z* 561 (M + Na^+, 100); HRMS (ESI) *m/z*: $[M + Na]^+$ Calcd for $C_{26}H_{23}{}^{79}BrN_2O_4SNa$ 561.0454; Found 561.0460.

Methyl (2*S*)-2-{[(benzyloxy)carbonyl]amino}-3-{8'-thia-1'-azatetracyclo[7.7.0.0^{2,7}.0^{11,16}]hexadeca-2'(7'),3',5',9',11',13',15'-heptaen-10'-yl}propanoate (9)

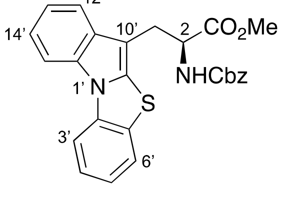

To a solution of *N*-[(benzyloxycarbonyl)amino]-[2'-(2"-bromophenylthio)]-L-tryptophan methyl ester (**8**) (0.845 g, 1.57 mmol) in dimethylacetamide (45 mL) was added copper(I) thiophene-2-carboxylate (0.299 g, 1.57 mmol). The reaction mixture was degassed under argon and stirred at 110 °C for 6 h. The reaction mixture was cooled to room temperature, extracted with ethyl acetate (50

mL) and washed with 5% aqueous lithium chloride (50 mL). The aqueous layer was further extracted with ethyl acetate (2 × 30 mL) and the combined organic layers were washed with 5% aqueous lithium chloride (2 × 50 mL), dried over $MgSO_4$, filtered and concentrated *in vacuo*. Purification by flash column chromatography (hexane/dichloromethane 2:3) gave methyl (2*S*)-2-{[(benzyloxy)carbonyl]amino}-3-{8'-thia-1'-azatetracyclo[7.7.0.0^{2,7}.0^{11,16}]hexadeca-2'(7'),3',5',9',11',13',15'-heptaen-10'-yl}propanoate (**9**) as a white solid (0.581 g, 81%). Mp 211–213 °C; IR (neat) 3315, 2950, 1699, 1505, 1473, 1208, 1055, 732 cm^{-1}; $[\alpha]_D^{24}$ +73.1 (*c* 0.1, $CHCl_3$); ^{1}H NMR (400 MHz, $CDCl_3$) δ 7.97–7.93 (m, 1H), 7.91 (ddd, 1H, *J* = 8.1, 1.1, 0.6 Hz), 7.62–7.50 (m, 2H), 7.44 (ddd, 1H, *J* = 8.1, 7.4, 1.2 Hz), 7.40–7.20 (m, 8H), 5.46 (d, 1H, *J* = 8.1 Hz), 5.22 (d, 1H, *J* = 12.4 Hz), 5.11 (d, 1H, *J* = 12.4 Hz), 4.82 (dt, 1H, *J* = 8.1, 5.0 Hz), 3.70 (s, 3H), 3.44 (d, 2H, *J* = 5.0 Hz); ^{13}C{^{1}H} NMR (101 MHz, $CDCl_3$) δ 172.1 (C), 155.8 (C), 136.5 (C), 136.1 (C), 135.5 (C), 133.0 (C), 131.3 (C), 130.2 (C), 128.6 (2 × CH), 128.32 (2 × CH), 128.27 (CH), 126.3 (CH), 123.9 (CH), 123.1 (CH), 121.6 (CH), 120.8 (CH), 118.0 (CH), 112.2 (CH), 111.1 (CH), 100.1 (C), 67.1 (CH_2), 54.6 (CH), 52.9 (CH_3), 28.3 (CH_2); MS (ESI) *m/z* 459 (M + H$^+$, 100); HRMS (ESI) *m/z*: [M + H]$^+$ Calcd for $C_{26}H_{22}N_2O_4SH$ 459.1373; Found 459.1369.

Methyl (2*S*)-2-{[(benzyloxy)carbonyl]amino}-3-{13'-bromo-8'-thia-1'-azatetracyclo[7.7.0.0^{2,7}.0^{11,16}]hexadeca-2'(7'),3',5',9',11',13',15'-heptaen-10'-yl}propanoate (10)

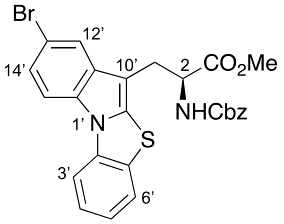

To a solution of methyl (2*S*)-2-{[(benzyloxy)carbonyl]amino}-3-{8-thia-1-azatetracyclo[7.7.0.0^{2,7}.0^{11,16}]hexadeca-2(7),3,5,9,11,13,15-heptaen-10-yl}propanoate (**9**) (0.585 g, 1.28 mmol) in anhydrous acetonitrile (100 mL) was added 11 drops of hydrobromic acid (48% in acetic acid) and the resulting reaction mixture was stirred at room temperature for 10 minutes. To the reaction mixture was added a solution of *N*-bromosuccinimide (0.238 g, 1.34 mmol) in acetonitrile (20 mL) dropwise over 10 minutes. The resulting reaction mixture was left to stir at room temperature in the dark for 4 h. The reaction mixture was concentrated *in vacuo*, diluted with dichloromethane (30 mL) and washed with saturated aqueous ammonium chloride (30 mL). The aqueous layer was further extracted with dichloromethane (2 × 30 mL). The combined organic layers were dried over $MgSO_4$, filtered and concentrated *in vacuo*. Purification by flash column chromatography (hexane/dichloromethane, 2:3) gave methyl (2*S*)-2-{[(benzyloxy)carbonyl]amino}-3-{13'-bromo-

8’-thia-1’-azatetracyclo[7.7.0.0^{2,7}.0^{11,16}]hexadeca-2’(7’),3’,5’,9’,11’,13’,15’-heptaen-10’-yl}propanoate (**10**) as an off-white solid (0.541 g, 79%). Mp 177−179 °C; IR (neat) 3307, 2947, 1736, 1687, 1527, 1479, 1292, 1225, 1026, 735 cm^{-1}; $[\alpha]_D^{23}$ +66.9 (*c* 0.1, $CHCl_3$); ^1H NMR (400 MHz, $CDCl_3$) δ 8.06 (d, 1H, J = 1.5 Hz), 7.83 (br d, 1H, J = 7.9 Hz), 7.55 (dd, 1H, J = 7.9, 1.2 Hz), 7.45 (td, 1H, J = 7.9, 1.2 Hz), 7.40–7.22 (m, 8H), 5.44 (d, 1H, J = 7.9 Hz), 5.22 (d, 1H, J = 12.2 Hz), 5.09 (d, 1H, J = 12.2 Hz), 4.79 (ddd, 1H, J = 7.9, 5.6, 4.6 Hz), 3.70 (s, 3H), 3.42 (dd, 1H, J = 14.9, 5.6 Hz), 3.37 (dd, 1H, J = 14.9, 4.6 Hz); $^{13}C\{^1H\}$ NMR (101 MHz, $CDCl_3$) δ 171.9 (C), 155.7 (C), 136.5 (C), 136.2 (C), 135.6 (C), 131.73 (C), 131.67 (C), 130.2 (C), 128.7 (2 × CH), 128.39 (2 × CH), 128.35 (CH), 126.5 (CH), 124.7 (CH), 124.0 (CH), 123.6 (CH), 119.1 (CH), 114.12 (C), 114.10 (CH), 112.3 (CH), 100.4 (C), 67.1 (CH_2), 54.6 (CH), 53.0 (CH_3), 28.2 (CH_2); MS (ESI) *m/z* 559 (M + Na$^+$, 100); HRMS (ESI) *m/z*: [M + Na]$^+$ Calcd for $C_{26}H_{21}{}^{79}BrN_2O_4SNa$ 559.0298; Found 559.0295.

Methyl (2*S*)-2-{[(benzyloxy)carbonyl]amino}-3-{13’-(4”-methoxyphenyl)-8’-thia-1’-azatetracyclo[7.7.0.0^{2,7}.0^{11,16}]hexadeca-2’(7’),3’,5’,9’,11’,13’,15’-heptaen-10’-yl}propanoate (11a)

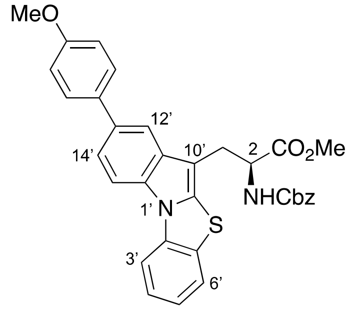

To a vial containing methyl (2*S*)-2-{[(benzyloxy)carbonyl]amino}-3-{13’-bromo-8’-thia-1’-azatetracyclo[7.7.0.0^{2,7}.0^{11,16}]hexadeca-2’(7’),3’,5’,9’,11’,13’,15’-heptaen-10’-yl}propanoate (**10**) (100 mg, 0.225 mmol) and water (2 mL) was added 4-methoxyphenylboronic acid (51.2 mg, 0.337 mmol) and potassium phosphate tribasic (95.5 mg, 0.450 mmol). The reaction mixture was degassed under argon prior to the addition of XPhos Pd G2 (8.85 mg, 0.0113 mmol) and tetrahydrofuran (2 mL). The reaction mixture was heated to 60 °C and stirred for 1 h. The reaction mixture was cooled to room temperature and concentrated *in vacuo*. The residue was dissolved in water (20 mL) and extracted with ethyl acetate (3 × 20 mL). The combined organic layers were dried over $MgSO_4$, filtered and concentrated *in vacuo*. Purification by flash column chromatography (hexane/dichloromethane, 2:3 + 0.5% ethyl acetate) gave methyl (2*S*)-2-{[(benzyloxy)carbonyl]amino}-3-{13’-(4”-methoxyphenyl)-8’-thia-1’-azatetracyclo[7.7.0.0^{2,7}.0^{11,16}]hexadeca-2’(7’),3’,5’,9’,11’,13’,15’-heptaen-10’-yl}propanoate (**11a**) as an off-white solid (84.0 mg, 80%). Mp 143−145 °C; IR (neat) 3304, 2954, 1688, 1516, 1474, 1245, 1026, 735 cm^{-1}; $[\alpha]_D^{22}$ +58.8 (*c* 0.1, $CHCl_3$); ^1H NMR (400 MHz, $CDCl_3$) δ 8.06 (d, 1H, J = 1.6 Hz),

7.95 (dd, 1H, J = 8.3, 1.0 Hz), 7.65–7.61 (m, 2H), 7.59–7.51 (m, 2H), 7.47–7.42 (m, 2H), 7.40–7.31 (m, 5H), 7.23 (td, 1H, J = 7.6, 1.0 Hz), 7.07–7.02 (m, 2H), 5.49 (d, 1H, J = 8.1 Hz), 5.24 (d, 1H, J = 12.2 Hz), 5.11 (d, 1H, J = 12.2 Hz), 4.83 (ddd, 1H, J = 8.1, 5.0, 4.6 Hz), 3.89 (s, 3H), 3.73 (s, 3H), 3.47 (dd, 1H, J = 15.1, 4.6 Hz), 3.42 (dd, 1H, J = 15.1, 5.0 Hz); $^{13}C\{^1H\}$ NMR (101 MHz, $CDCl_3$) δ 172.1 (C), 159.0 (C), 155.8 (C), 136.5 (C), 136.1 (C), 135.7 (C), 134.8 (C), 134.2 (C), 132.0 (C), 131.8 (C), 130.3 (C), 128.63 (2 × CH), 128.61 (2 × CH), 128.35 (2 × CH), 128.27 (CH), 126.3 (CH), 123.9 (CH), 123.2 (CH), 121.1 (CH), 118.2 (CH), 114.4 (2 × CH), 112.2 (CH), 109.4 (CH), 100.1 (C), 67.1 (CH_2), 55.5 (CH_3), 54.6 (CH), 52.9 (CH_3), 28.3 (CH_2); MS (ESI) *m/z* 587 (M + Na^+, 100); HRMS (ESI) *m/z*: $[M + Na]^+$ Calcd for $C_{33}H_{28}N_2O_5SNa$ 587.1611; Found 587.1622.

Methyl (2*S*)-2-{[(benzyloxy)carbonyl]amino}-3-{13’-(2”-methoxyphenyl)-8’-thia-1’-azatetracyclo[7.7.0.0^{2,7}.0^{11,16}]hexadeca-2’(7’),3’,5’,9’,11’,13’,15’-heptaen-10’-yl}propanoate (11b)

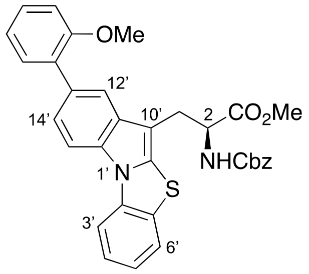

To a vial containing methyl (2*S*)-2-{[(benzyloxy)carbonyl]amino}-3-{13’-bromo-8’-thia-1’-azatetracyclo[7.7.0.0^{2,7}.0^{11,16}]hexadeca-2’(7’),3’,5’,9’,11’,13’,15’-heptaen-10’-yl}propanoate (**10**) (60.0 mg, 0.112 mmol) and water (1 mL) was added 2-methoxyphenylboronic acid (25.0 mg, 0.167 mmol) and potassium phosphate tribasic (48.0 mg, 0.224 mmol). The reaction mixture was degassed under argon prior to the addition of XPhos Pd G2 (4.50 mg, 0.00575 mmol) and tetrahydrofuran (1 mL). The reaction mixture was heated to 60 °C and stirred for 2 h. The reaction mixture was cooled to room temperature and concentrated *in vacuo*. The residue was dissolved in water (30 mL) and extracted with ethyl acetate (3 × 30 mL). The combined organic layers were dried over $MgSO_4$, filtered and concentrated *in vacuo*. Purification by flash column chromatography (hexane/dichloromethane, 2:3 + 0.5% ethyl acetate) gave methyl (2*S*)-2-{[(benzyloxy)carbonyl]amino}-3-{13’-(2”-methoxyphenyl)-8’-thia-1’-azatetracyclo[7.7.0.0^{2,7}.0^{11,16}]hexadeca-2’(7’),3’,5’,9’,11’,13’,15’-heptaen-10’-yl}propanoate (**11b**) as a yellow solid (51.0 mg, 79%). Mp 129−131 °C; IR (neat) 3435, 2943, 2359, 1707, 1504, 1351, 1180, 1027, 699 cm^{-1}; $[\alpha]_D^{24}$ +60.1 (*c* 0.1, $CHCl_3$); ^1H NMR (400 MHz, $CDCl_3$) δ 8.14 (d, 1H, J = 1.5 Hz), 7.91 (br d, 1H, J = 8.0 Hz), 7.61–7.52 (m, 2H), 7.47–7.31 (m, 9H), 7.22 (td, 1H, J = 8.0, 0.9 Hz), 7.11 (td, 1H, J = 7.4, 1.1 Hz), 7.06 (dd, 1H, J = 8.0, 1.5 Hz), 5.51 (d, 1H, J = 8.1 Hz), 5.24 (d,

1H, *J* = 12.3 Hz), 5.13 (d, 1H, *J* = 12.3 Hz), 4.85 (ddd, 1H, *J* = 8.1, 5.3, 5.1 Hz), 3.86 (s, 3H), 3.74 (s, 3H), 3.49 (dd, 2H, *J* = 15.0, 5.3 Hz), 3.46 (dd, 1H, *J* = 15.0, 5.1 Hz); $^{13}C\{^1H\}$ NMR (101 MHz, $CDCl_3$) *δ* 172.1 (C), 156.8 (C), 155.8 (C), 136.6 (C), 136.2 (C), 135.7 (C), 131.9 (C), 131.52 (C), 131.45 (CH), 131.43 (C), 131.35 (C), 130.3 (C), 128.6 (2 × CH), 128.5 (CH), 128.3 (2 × CH), 128.2 (CH), 126.3 (CH), 123.8 (CH), 123.6 (CH), 123.1 (CH), 121.1 (CH), 117.4 (CH), 112.3 (CH), 112.2 (CH), 111.6 (CH), 100.2 (C), 67.1 (CH_2), 55.8 (CH_3), 54.7 (CH), 52.9 (CH_3), 28.3 (CH_2); MS (ESI) *m/z* 587 (M + Na^+, 100); HRMS (ESI) *m/z*: $[M + Na]^+$ Calcd for $C_{33}H_{28}N_2O_5SNa$ 587.1611; Found 587.1627.

Methyl (2*S*)-2-{[(benzyloxy)carbonyl]amino}-3-{13'-[(3",4"-methylenedioxy)phenyl]-8'-thia-1'-azatetracyclo[7.7.0.0^{2,7}.0^{11,16}]hexadeca-2'(7'),3',5',9',11',13',15'-heptaen-10'-yl}propanoate (11c)

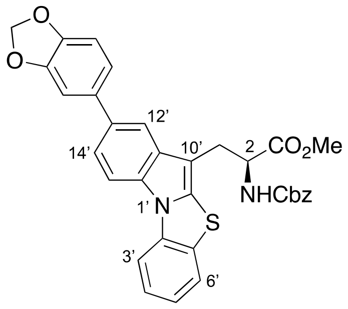

To a vial containing methyl (2*S*)-2-{[(benzyloxy)carbonyl]amino}-3-{13'-bromo-8'-thia-1'-azatetracyclo[7.7.0.0^{2,7}.0^{11,16}]hexadeca-2'(7'),3',5',9',11',13',15'-heptaen-10'-yl}propanoate (**10**) (60.0 mg, 0.112 mmol) and water (1 mL) was added 3,4-(methylenedioxy)phenylboronic acid (28.0 mg, 0.167 mmol) and potassium phosphate tribasic (48.0 mg, 0.224 mmol). The reaction mixture was degassed under argon prior to the addition of XPhos Pd G2 (4.50 mg, 0.00575 mmol) and tetrahydrofuran (1 mL). The reaction mixture was heated to 60 °C and stirred for 2 h. The reaction mixture was cooled to room temperature and concentrated *in vacuo*. The residue was dissolved in water (30 mL) and extracted with ethyl acetate (3 × 30 mL). The combined organic layers were dried over $MgSO_4$, filtered and concentrated *in vacuo*. Purification by flash column chromatography (hexane/dichloromethane, 2:3 + 0.5% ethyl acetate) gave methyl (2*S*)-2-{[(benzyloxy)carbonyl]amino}-3-{13'-[(3",4"-methylenedioxy)phenyl]-8'-thia-1'-azatetracyclo[7.7.0.0^{2,7}.0^{11,16}]hexadeca-2'(7'),3',5',9',11',13',15'-heptaen-10'-yl}propanoate (**11c**) as a white solid (48.0 mg, 74%). Mp 129−131 °C; IR (neat) 3316, 2898, 2360, 1738, 1689, 1471, 1222, 1032, 734 cm^{-1}; $[\alpha]_D^{24}$ +52.9 (*c* 0.1, $CHCl_3$); ^1H NMR (400 MHz, $CDCl_3$) *δ* 8.02 (d, 1H, *J* = 1.5 Hz), 7.93 (dd, 1H, *J* = 8.0, 1.5 Hz), 7.61–7.50 (m, 2H), 7.47–7.29 (m, 7H), 7.23 (t, 1H, *J* = 7.7 Hz), 7.19–7.13 (m, 2H), 6.94 (d, 1H, *J* = 8.0 Hz), 6.03 (s, 2H), 5.50 (d, 1H, *J* = 7.9 Hz), 5.24 (d, 1H, *J* = 12.3 Hz), 5.11 (d, 1H, *J* = 12.3 Hz), 4.83 (dt, 1H, *J* = 7.9, 5.0 Hz), 3.73 (s, 3H), 3.46 (dd, 1H, *J* =

14.1, 5.0 Hz), 3.41 (dd, 1H, *J* = 14.1, 5.0 Hz); $^{13}C\{^1H\}$ NMR (101 MHz, $CDCl_3$) *δ* 172.1 (C), 155.8 (C), 148.3 (C), 146.9 (C), 136.6 (C), 136.5 (C), 136.1 (C), 135.9 (C), 134.2 (C), 132.0 (C), 131.9 (C), 130.3 (C), 128.6 (2 × CH), 128.4 (2 × CH), 128.3 (CH), 126.4 (CH), 123.9 (CH), 123.2 (CH), 121.2 (CH), 121.0 (CH), 118.2 (CH), 112.3 (CH), 109.5 (CH), 108.8 (CH), 108.2 (CH), 101.3 (CH_2), 100.2 (C), 67.1 (CH_2), 54.6 (CH), 52.9 (CH_3), 28.3 (CH_2); MS (ESI) *m/z* 601 (M + Na^+, 100); HRMS (ESI) *m/z*: $[M + Na]^+$ Calcd for $C_{33}H_{26}N_2O_6SNa$ 601.1404; Found 601.1419.

Methyl (2*S*)-2-{[(benzyloxy)carbonyl]amino}-3-{13'-(4"-cyanophenyl)-8'-thia-1'-azatetracyclo[7.7.0.0^{2,7}.0^{11,16}]hexadeca-2'(7'),3',5',9',11',13',15'-heptaen-10'-yl}propanoate (11d)

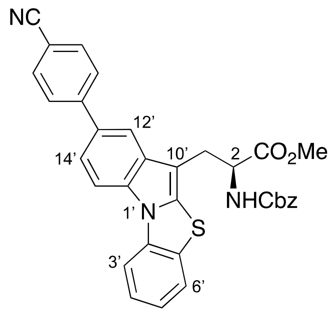

To a vial containing methyl (2*S*)-2-{[(benzyloxy)carbonyl]amino}-3-{13'-bromo-8'-thia-1'-azatetracyclo[7.7.0.0^{2,7}.0^{11,16}]hexadeca-2'(7'),3',5',9',11',13',15'-heptaen-10'-yl}propanoate (**10**) (200 mg, 0.372 mmol) and water (3 mL) was added 4-cyanophenylboronic acid (82.0 mg, 0.558 mmol) and potassium phosphate tribasic (158 mg, 0.744 mmol). The reaction mixture was degassed under argon prior to the addition of XPhos Pd G2 (14.6 mg, 0.0186 mmol) and tetrahydrofuran (3 mL). The reaction mixture was heated to 60 °C and stirred for 1 h. The reaction mixture was cooled to room temperature and concentrated *in vacuo*. The residue was dissolved in water (30 mL) and extracted with ethyl acetate (3 × 30 mL). The combined organic layers were dried over $MgSO_4$, filtered and concentrated *in vacuo*. Purification by flash column chromatography (hexane/dichloromethane, 2:3 + 0.5% ethyl acetate) gave methyl (2*S*)-2-{[(benzyloxy)carbonyl]amino}-3-{13'-(4"-cyanophenyl)-8'-thia-1'-azatetracyclo[7.7.0.0^{2,7}.0^{11,16}]hexadeca-2'(7'),3',5',9',11',13',15'-heptaen-10'-yl}propanoate (**11d**) as a yellow solid (165 mg, 79%). Mp 218–219 °C; IR (neat) 3316, 2898, 2222, 1689, 1531, 1474, 1250, 1028, 736 cm^{-1}; $[\alpha]_D^{22}$ +55.9 (*c* 0.1, $CHCl_3$); ^1H NMR (400 MHz, $CDCl_3$) *δ* 8.11 (d, 1H, *J* = 1.5 Hz), 7.95 (br d, 1H, *J* = 8.1 Hz), 7.80 (d, 2H, *J* = 8.0 Hz), 7.76 (d, 2H, *J* = 8.0 Hz), 7.64–7.58 (m, 2H), 7.51–7.42 (m, 2H), 7.40–7.25 (m, 6H), 5.48 (d, 1H, *J* = 7.9 Hz), 5.24 (d, 1H, *J* = 12.3 Hz), 5.10 (d, 1H, *J* = 12.3 Hz), 4.83 (ddd, 1H, *J* = 7.9, 5.4, 4.4 Hz), 3.73 (s, 3H), 3.49 (dd, 1H, *J* = 15.1, 5.4 Hz), 3.44 (dd, 1H, *J* = 15.1, 4.4 Hz); $^{13}C\{^1H\}$ NMR (101 MHz, $CDCl_3$) *δ* 172.0 (C), 155.8 (C), 146.7 (C), 137.3 (C), 136.5 (C), 135.9 (C), 133.2 (C), 132.8 (2 × CH), 132.0 (C), 131.8 (C), 130.3 (C),

128.7 (2 × CH), 128.41 (2 × CH), 128.35 (CH), 128.1 (2 × CH), 126.6 (CH), 124.1 (CH), 123.6 (CH), 121.1 (CH), 119.3 (C), 118.6 (CH), 112.3 (CH), 110.4 (C), 109.9 (CH), 100.4 (C), 67.1 (CH_2), 54.6 (CH), 53.0 (CH_3), 28.3 (CH_2); MS (ESI) *m/z* 560 (M + H^+, 100); HRMS (ESI) *m/z*: $[M + Na]^+$ Calcd for $C_{33}H_{25}N_3O_4SH$ 560.1639; Found 560.1642.

Methyl (2*S*)-2-{[(benzyloxy)carbonyl]amino}-3-{13'-[4"-acetylphenyl]-8'-thia-1'-azatetracyclo[7.7.0.0^{2,7}.0^{11,16}]hexadeca-2'(7'),3',5',9',11',13',15'-heptaen-10'-yl}propanoate (11e)

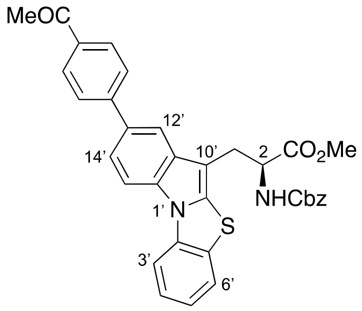

To a vial containing methyl (2*S*)-2-{[(benzyloxy)carbonyl]amino}-3'-{13'-bromo-8'-thia-1'-azatetracyclo[7.7.0.0^{2,7}.0^{11,16}]hexadeca-2'(7'),3',5',9',11',13',15'-heptaen-10'-yl}propanoate (**10**) (200 mg, 0.372 mmol) and water (3 mL) was added 4-acetylphenylboronic acid (92.0 mg, 0.558 mmol) and potassium phosphate tribasic (158 mg, 0.744 mmol). The reaction mixture was degassed under argon prior to the addition of XPhos Pd G2 (15.0 mg, 0.0186 mmol) and tetrahydrofuran (3 mL). The reaction mixture was heated to 60 °C and stirred for 2 h. The reaction mixture was cooled to room temperature and concentrated *in vacuo*. The residue was dissolved in water (30 mL) and extracted with ethyl acetate (3 × 30 mL). The combined organic layers were dried over $MgSO_4$, filtered and concentrated *in vacuo*. Purification by flash column chromatography (hexane/dichloromethane, 1:9 + 0.5% ethyl acetate) gave methyl (2*S*)-2-{[(benzyloxy)carbonyl]amino}-3-{13'-[4"-acetylphenyl]-8'-thia-1'-azatetracyclo[7.7.0.0^{2,7}.0^{11,16}]hexadeca-2'(7'),3',5',9',11',13',15'-heptaen-10'-yl}propanoate (**11e**) as a yellow solid (189 mg, 88%). Mp 201−203 °C; IR (neat) 3314, 2908, 2356, 1695, 1674, 1533, 1476, 1254, 1024, 733 cm^{-1}; $[\alpha]_D^{26}$ +56.3 (*c* 0.1, $CHCl_3$); ^1H NMR (400 MHz, $CDCl_3$) δ 8.15 (d, 1H, J = 2.0 Hz), 8.09 (d, 2H, J = 8.0 Hz), 7.97 (d, 1H, J = 7.3 Hz), 7.80 (d, 2H, J = 8.0 Hz), 7.63–7.54 (m, 2H), 7.52–7.44 (m, 2H), 7.40–7.24 (m, 6H), 5.48 (d, 1H, J = 8.0 Hz), 5.24 (d, 1H, J = 12.3 Hz), 5.11 (d, 1H, J = 12.3 Hz), 4.83 (ddd, 1H, J = 8.0, 5.5, 4.7 Hz), 3.73 (s, 3H), 3.47 (dd, 1H, J = 15.0, 5.5 Hz), 3.43 (dd, 1H, J = 15.0, 4.7 Hz), 2.67 (s, 3H); $^{13}C\{^1H\}$ NMR (101 MHz, $CDCl_3$) δ 197.9 (C), 172.0 (C), 155.8 (C), 146.8 (C), 136.9 (C), 136.5 (C), 136.0 (C), 135.6 (C), 133.0 (C), 132.8 (C), 131.9 (C), 130.3 (C), 129.2 (2 × CH), 128.7 (2 × CH), 128.4 (2 × CH), 128.3 (CH), 127.6 (2 × CH), 126.5 (CH), 124.0 (CH), 123.5 (CH), 121.3 (CH), 118.5 (CH), 112.4 (CH), 109.9 (CH), 100.4 (C),

67.1 (CH_2), 54.6 (CH), 53.0 (CH_3), 28.3 (CH_2), 26.8 (CH_3); MS (ESI) *m/z* 599 (M + Na^+, 100); HRMS (ESI) *m/z*: $[M + Na]^+$ Calcd for $C_{34}H_{28}N_2O_5SNa$ 599.1611; Found 599.1623.

Methyl (2*S*)-2-{[(benzyloxy)carbonyl]amino}-3-{13'-[4"-trifluoromethylphenyl]-8'-thia-1'-azatetracyclo[7.7.0.0^{2,7}.0^{11,16}]hexadeca-2'(7'),3',5',9',11',13',15'-heptaen-10'-yl}propanoate (11f)

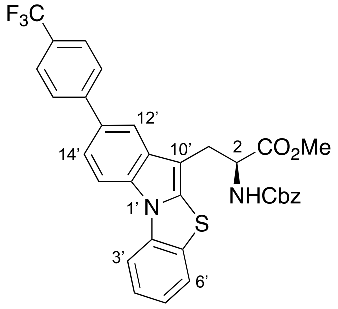

To a vial containing methyl (2*S*)-2-{[(benzyloxy)carbonyl]amino}-3-{13'-bromo-8'-thia-1'-azatetracyclo[7.7.0.0^{2,7}.0^{11,16}]hexadeca-2'(7'),3',5',9',11',13',15'-heptaen-10'-yl}propanoate (**10**) (200 mg, 0.372 mmol) and water (3 mL) was added 4-trifluoromethylphenylboronic acid (106 mg, 0.558 mmol) and potassium phosphate tribasic (158 mg, 0.744 mmol). The reaction mixture was degassed under argon prior to the addition of XPhos Pd G2 (15.0 mg, 0.0186 mmol) and tetrahydrofuran (3 mL). The reaction mixture was heated to 60 °C and stirred for 2 h. The reaction mixture was cooled to room temperature and concentrated *in vacuo*. The residue was dissolved in water (30 mL) and extracted with ethyl acetate (3 × 30 mL). The combined organic layers were dried over $MgSO_4$, filtered and concentrated *in vacuo*. Purification by flash column chromatography (hexane/dichloromethane, 1:1 + 0.5% ethyl acetate) gave methyl (2*S*)-2-{[(benzyloxy)carbonyl]amino}-3-{13'-[4"-trifluoromethylphenyl]-8'-thia-1'-azatetracyclo[7.7.0.0^{2,7}.0^{11,16}]hexadeca-2'(7'),3',5',9',11',13',15'-heptaen-10'-yl}propanoate (**11f**) as an off-white solid (198 mg, 88%). Mp 221−223 °C; IR (neat) 3310, 2959, 1739, 1691, 1476, 1329, 1257, 1106, 1066, 737 cm^{-1}; $[\alpha]_D^{26}$ +49.3 (*c* 0.1, $CHCl_3$); ^{1}H NMR (400 MHz, $CDCl_3$) δ 8.11 (d, 1H, J = 1.6 Hz), 7.95 (d, 1H, J = 7.9 Hz), 7.80 (d, 2H, J = 8.5 Hz), 7.74 (d, 2H, J = 8.5 Hz), 7.63–7.54 (m, 2H), 7.50–7.43 (m, 2H), 7.41–7.30 (m, 5H), 7.27–7.22 (m, 1H), 5.49 (d, 1H, J = 7.9 Hz), 5.25 (d, 1H, J = 12.3 Hz), 5.11 (d, 1H, J = 12.3 Hz), 4.84 (ddd, 1H, J = 7.9, 5.4, 4.4 Hz), 3.73 (s, 3H), 3.48 (dd, 1H, J = 15.0, 5.4 Hz), 3.43 (dd, 1H, J = 15.0, 4.4 Hz); $^{13}C\{^1H\}$ NMR (101 MHz, $CDCl_3$) δ 172.0 (C), 155.8 (C), 145.7 (C), 136.8 (C), 136.5 (C), 136.0 (C), 132.9 (C), 132.7 (C), 131.9 (C), 130.3 (C), 129.1 (q, $^2J_{CF}$ = 32.4 Hz, C), 128.7 (2 × CH), 128.4 (2 × CH), 128.3 (CH), 127.8 (2 × CH), 126.5 (CH), 125.9 (q, $^3J_{CF}$ = 3.6 Hz, 2 × CH), 124.9 (q, $^1J_{CF}$ = 273.3 Hz, C), 124.0 (CH), 123.5 (CH), 121.2 (CH), 118.5 (CH), 112.3 (CH), 109.9 (CH), 100.3 (C), 67.1 (CH_3), 54.6 (CH), 53.0 (CH_3), 28.3 (CH_2);

MS (ESI) *m/z* 603 (M + H$^+$, 100); HRMS (ESI) *m/z*: [M + Na]$^+$ Calcd for $C_{33}H_{25}F_3N_2O_4SH$ 603.1560; Found 603.1557.

(2*S*)-2-Amino-3-{13'-[4''-acetylphenyl]-8'-thia-1'-azatetracyclo[7.7.0.0^{2,7}.0^{11,16}]hexadeca-2'(7'),3',5',9',11',13',15'-heptaen-10'-yl}propanoic acid hydrochloride (12a)

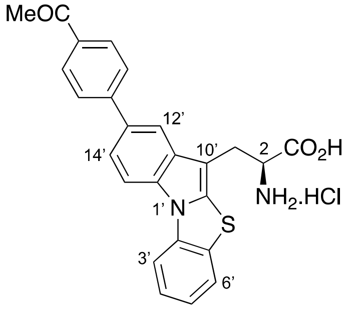

To a stirred solution of methyl (2*S*)-2-{[(benzyloxy)carbonyl]amino}-3-{13'-[4''-acetylphenyl]-8'-thia-1'-azatetracyclo[7.7.0.0^{2,7}.0^{11,16}]hexadeca-2'(7'),3',5',9',11',13',15'-heptaen-10'-yl}propanoate (**11e**) (175 mg, 0.303 mmol) in methanol (10 mL), 1,4-dioxane (5 mL) and water (5 mL) was added caesium carbonate (129 mg, 0.395 mmol). The reaction mixture was left to stir at 60 °C for 5 h. The reaction mixture was concentrated *in vacuo,* diluted with water (10 mL) and acidified to pH 1 with 1 M aqueous hydrochloric acid. The aqueous layer was extracted with dichloromethane (3 × 20 mL). The combined organic layers were dried over $MgSO_4$, filtered and concentrated under reduced pressure to give (2*S*)-2-{[(benzyloxy)carbonyl]amino}-3-{13'-[4''-acetylphenyl]-8'-thia-1'-azatetracyclo[7.7.0.0^{2,7}.0^{11,16}]hexadeca-2'(7'),3',5',9',11',13',15'-heptaen-10'-yl}propanoic acid as a yellow solid (171 mg, 100%). This was used for the next reaction without any further purification. A suspension of (2*S*)-2-{[(benzyloxy)carbonyl]amino}-3-{13'-[4''-acetylphenyl]-8'-thia-1'-azatetracyclo[7.7.0.0^{2,7}.0^{11,16}]hexadeca-2'(7'),3',5',9',11',13',15'-heptaen-10'-yl}propanoic acid (50.0 mg, 0.0889 mmol) in 4 M hydrochloric acid in dioxane (8 mL) was heated under reflux for 4 h. After cooling to room temperature, the reaction mixture was concentrated *in vacuo* and the resulting residue was recrystallized from ethanol and diethyl ether to give amino acid (**12a**) as a dark brown solid (20.0 mg, 49%). Mp 312−314 °C (decomposition); IR (neat) 2901, 1760, 1676, 1600, 1474, 1266, 1197, 1092, 735 cm^{-1}; $[\alpha]_D^{24}$ −40.0 (*c* 0.01, DMSO); ^{1}H NMR (400 MHz, DMSO-*d*$_6$) δ 8.55 (d, 1H, *J* = 1.6 Hz), 8.51 (d, 1H, *J* = 7.9, 1.0 Hz), 8.44 (s, 2H), 8.13–8.01 (m, 4H), 7.96 (br d, 1H, *J* = 7.9, 1.2 Hz), 7.88 (d, 1H, *J* = 8.5 Hz), 7.73 (dd, 1H, *J* = 8.5, 1.6 Hz), 7.57 (td, 1H, *J* = 7.9, 1.2 Hz), 7.37 (td, 1H, *J* = 7.9, 1.0 Hz), 4.20 (t, 1H, *J* = 6.2 Hz), 3.55–3.40 (m, 2H), 2.64 (s, 3H); $^{13}C\{^1H\}$ NMR (101 MHz, DMSO-*d*$_6$) δ 197.5 (C), 170.5 (C), 145.3 (C), 137.1 (C), 135.1 (C), 135.0 (C), 132.1 (C), 131.8 (C), 131.4 (C), 129.5 (C), 128.8 (2 × CH), 127.3 (2 × CH), 126.8 (CH), 124.3 (CH), 123.8 (CH), 120.8 (CH), 118.6 (CH), 113.1 (CH), 109.6 (CH), 99.0 (C), 52.1 (CH), 26.8 (CH_3), 26.0 (CH_2);

MS (ESI) *m/z* 429 (M + H^+, 100); HRMS (ESI) *m/z*: $[M + H]^+$ Calcd for $C_{25}H_{20}N_2O_3SH$ 429.1267; Found 429.1264.

(2*S*)-2-Amino-3-{13'-[4"-trifluoromethylphenyl]-8'-thia-1'-azatetracyclo[7.7.0.0^{2,7}.0^{11,16}]hexadeca-2'(7'),3',5',9',11',13',15'-heptaen-10'-yl}propanoic acid hydrochloride (12b)

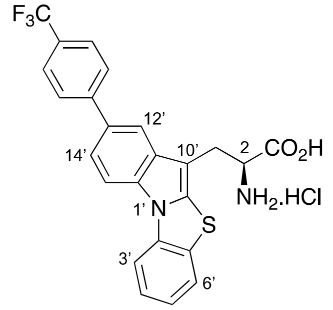

To a stirred solution of methyl (2*S*)-2-{[(benzyloxy)carbonyl]amino}-3-{13'-[4"-trifluoromethylphenyl]-8'-thia-1'-azatetracyclo[7.7.0.0^{2,7}.0^{11,16}]hexadeca-2'(7'),3',5',9',11',13',15'-heptaen-10'-yl}propanoate (**11f**) (260 mg, 0.431 mmol) in methanol (14.0 mL), 1,4-dioxane (7.0 mL) and water (7.0 mL) was added caesium carbonate (183 mg, 0.561 mmol). The reaction mixture was left to stir at 60 °C for 5 h. The reaction mixture was concentrated *in vacuo,* diluted with water (10 mL) and acidified to pH 1 with 1 M aqueous hydrochloric acid. The aqueous layer was extracted with dichloromethane (3 × 20 mL). The combined organic layers were dried over $MgSO_4$, filtered and concentrated under reduced pressure to give (2*S*)-2-{[(benzyloxy)carbonyl]amino}-3-{13'-[4"-trifluoromethylphenyl]-8'-thia-1'-azatetracyclo[7.7.0.0^{2,7}.0^{11,16}]hexadeca-2'(7'),3',5',9',11',13',15'-heptaen-10'-yl}propanoic acid as a white solid (254 mg, 100%). This was used for the next reaction without any further purification. A suspension of (2*S*)-2-{[(benzyloxy)carbonyl]amino}-3-{13'-[4"-trifluoromethylphenyl]-8'-thia-1'-azatetracyclo[7.7.0.0^{2,7}.0^{11,16}]hexadeca-2'(7'),3',5',9',11',13',15'-heptaen-10'-yl}propanoic acid (75.0 mg, 0.127 mmol) in 6 M aqueous hydrochloric acid (9 mL) and 1,4-dioxane (4.5 mL) was heated under reflux in a sealed tube for 4 h. After cooling to room temperature, the reaction mixture was concentrated *in vacuo* and the resulting residue was recrystallized from ethanol and diethyl ether to give amino acid (**12b**) as an off-white solid (44.0 mg, 70%). Mp 315−317 °C (decomposition); IR (neat) 3104, 2890, 1762, 1587, 1477, 1330, 1108, 1070, 736 cm^{-1}; $[\alpha]_D^{18}$ −44.0 (*c* 0.1, DMSO); ^{1}H NMR (400 MHz, DMSO-d_6) δ 8.61–8.44 (m, 4H), 8.12 (d, 2H, J = 8.4 Hz), 7.96 (dd, 1H, J = 7.7, 1.3 Hz), 7.91 (d, 1H, J = 8.4 Hz), 7.84 (d, 2H, J = 8.4 Hz), 7.70 (dd, 1H, J = 8.4, 1.6 Hz), 7.56 (td, 1H, J = 7.7, 1.3 Hz), 7.37 (td, 1H, J = 7.7, 1.1 Hz), 4.18 (dd, 1H, J = 7.0, 6.1 Hz), 3.48 (dd, 1H, J = 15.0, 6.1 Hz), 3.42 (dd, 1H, J = 15.0, 7.0 Hz); ^{13}C{^{1}H} NMR (101 MHz, DMSO-d_6) δ 170.5 (C), 144.9 (C), 137.1 (C), 135.0 (C), 132.1 (C), 131.6 (C), 131.3 (C), 129.5 (C), 127.9 (2 × CH), 127.2 (q, $^2J_{CF}$ = 33.0 Hz, C), 126.8 (CH), 125.6 (q, $^3J_{CF}$ = 3.7 Hz, 2 × CH),

124.5 (q, ${}^{1}J_{CF}$ = 272.1 Hz, C), 124.3 (CH), 123.8 (CH), 120.8 (CH), 118.7 (CH), 113.1 (CH), 109.8 (CH), 99.0 (C), 52.1 (CH), 26.0 (CH_2); MS (ESI) *m/z* 455 (M + H^+, 100); HRMS (ESI) *m/z*: $[M + H]^+$ Calcd for $C_{24}H_{17}F_3N_2O_2SH$ 455.1036; Found 455.1037.

(2*S*)-2-[(9*H*-Fluoren-9-ylmethoxycarbonyl)amino-3-{13'-[4"-trifluoromethylphenyl]-8'-thia-1'-azatetracyclo[7.7.0.0^{2,7}.0^{11,16}]hexadeca-2'(7'),3',5',9',11',13',15'-heptaen-10'-yl}propanoic acid (13)

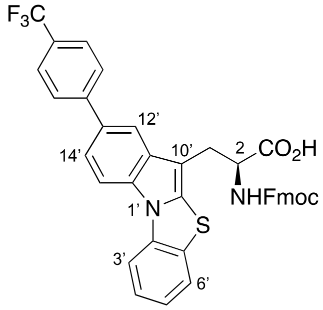

To a suspension of (2*S*)-2-amino-3-{13'-[4"-trifluoromethylphenyl]-8'-thia-1'-azatetracyclo[7.7.0.0^{2,7}.0^{11,16}]hexadeca-2'(7'),3',5',9',11',13',15'-heptaen-10'-yl}propanoic acid hydrochloride (**12b**) (17.0 mg, 0.0346 mmol) in 1,4-dioxane (0.5 mL) and water (0.5 mL) was added sodium hydrogencarbonate (11.6 mg, 0.138 mmol) followed by *N*-(9-fluorenylmethoxycarbonyloxy)succinimide (11.4 mg, 0.0339 mmol). The reaction mixture was stirred at room temperature for 24 h, acidified to pH 2 with 1 M aqueous hydrochloric acid and concentrated *in vacuo*. The resulting residue was recrystallized from chloroform and diethyl ether to give (2*S*)-2-[(9*H*-fluoren-9-ylmethoxycarbonyl)amino-3-{13-[4"-trifluoromethylphenyl]-8-thia-1-azatetracyclo[7.7.0.0^{2,7}.0^{11,16}]hexadeca-2(7),3,5,9,11,13,15-heptaen-10-yl}propanoic acid (**13**) as an off-white solid (13.0 mg, 57%). Mp 215−217 °C (decomposition); IR (neat) 3302, 3043, 2917, 1690, 1541, 1476, 1328, 1107, 735 cm^{-1}; $[\alpha]_D^{22}$ +26.7 (*c* 0.1, DMSO); ^{1}H NMR (400 MHz, DMSO-d_6) δ 12.86 (br s, 1H), 8.49 (br s, 1H), 8.45 (br d, 1H, *J* = 8.0 Hz), 8.06 (d, 2H, *J* = 8.1 Hz), 7.89–7.80 (m, 7H), 7.68–7.59 (m, 3H), 7.54 (t, 1H, *J* = 8.0 Hz), 7.38–7.32 (m, 3H), 7.24 (t, 1H, *J* = 7.5 Hz), 7.16 (t, 1H, *J* = 7.5 Hz), 4.36 (td, 1H, *J* = 8.8, 5.2 Hz), 4.27–4.10 (m, 3H), 3.39 (dd, 1H, *J* = 14.7, 5.2 Hz), 3.26 (dd, 1H, *J* = 14.7, 8.8 Hz); ^{13}C data unavailable due to compound decomposition in DMSO-d_6 over time;[4] MS (ESI) *m/z* 677 (M + H^+, 100); HRMS (ESI) *m/z*: $[M + H]^+$ Calcd for $C_{39}H_{27}F_3N_2O_4SH$ 677.1716; Found 677.1718.

4. Photophysical Data for α-Amino Acids

Spectra were recorded at 2 μM in DMSO using an excitation and emission bandpass of 5 nm.

Absorption and Emission Spectra for 11a.

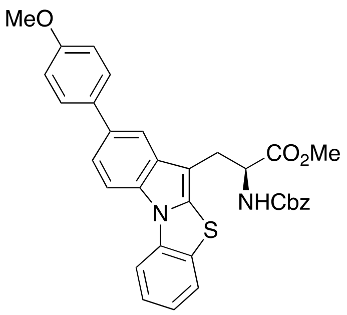

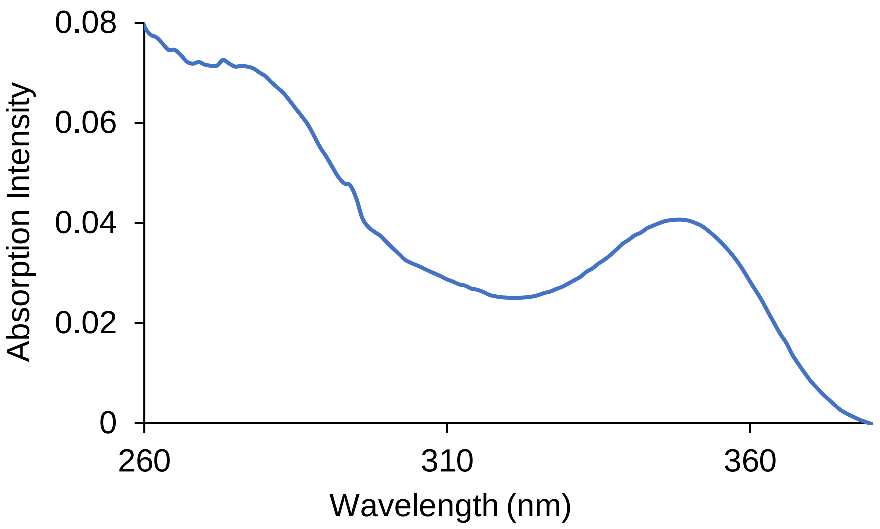

Excitation at 348 nm:

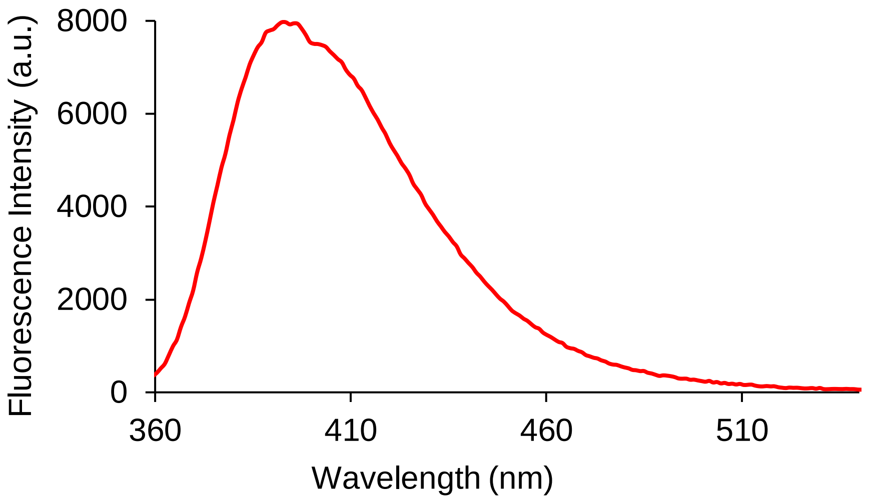

Absorption and Emission Spectra for 11b.

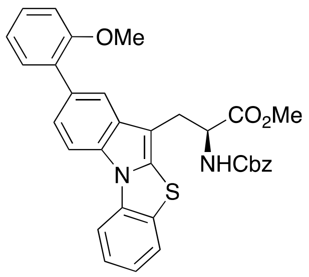

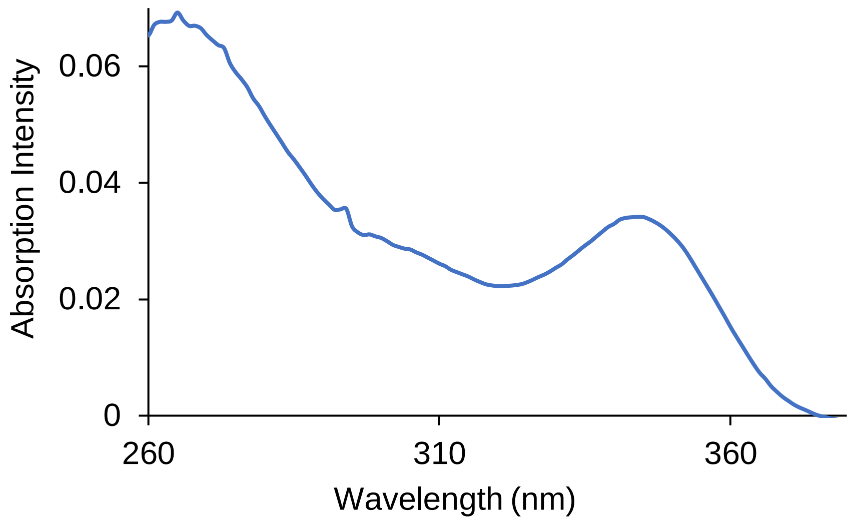

Excitation at 343 nm:

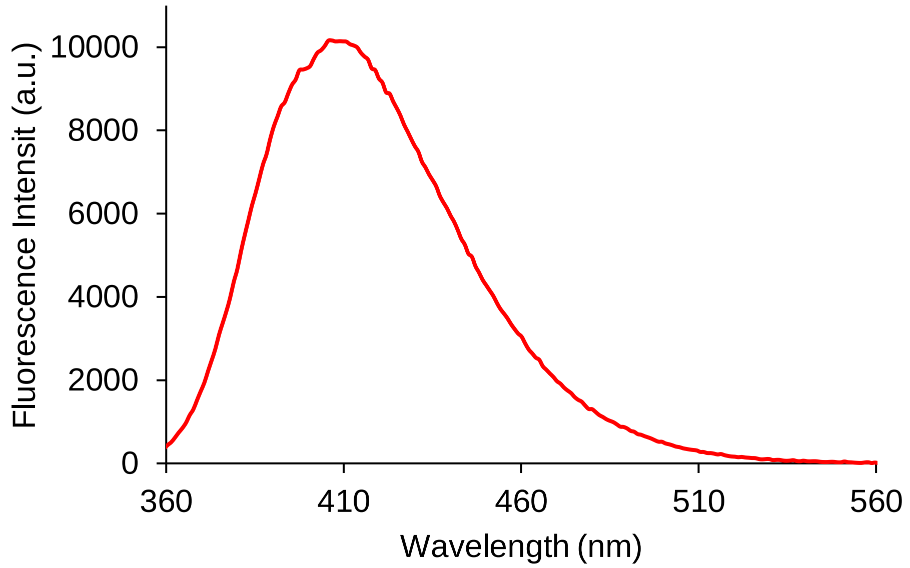

Absorption and Emission Spectra for 11c.

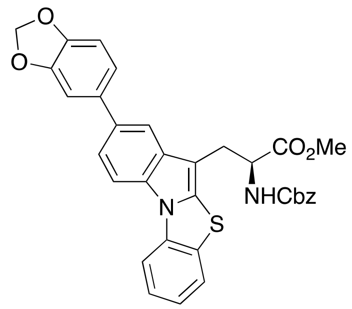

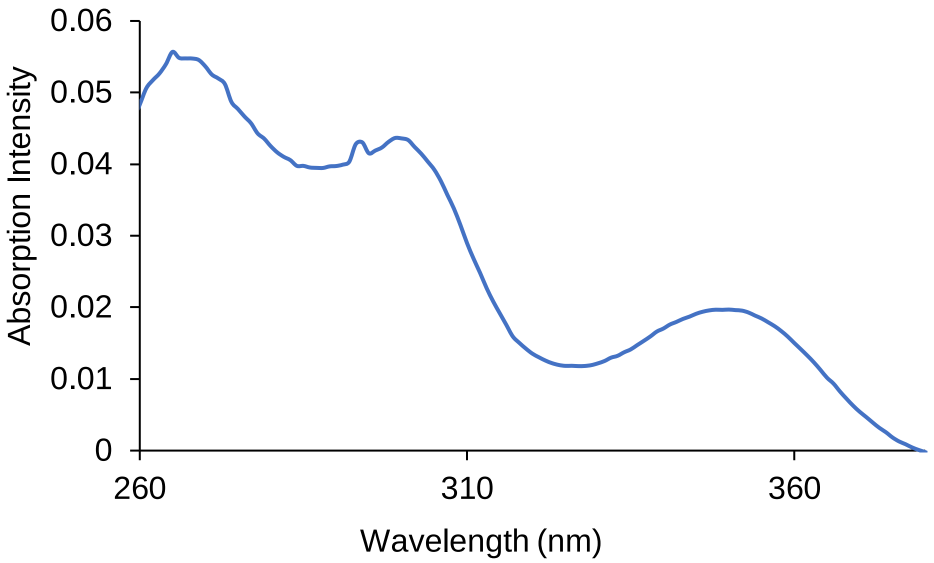

Excitation at 350 nm:

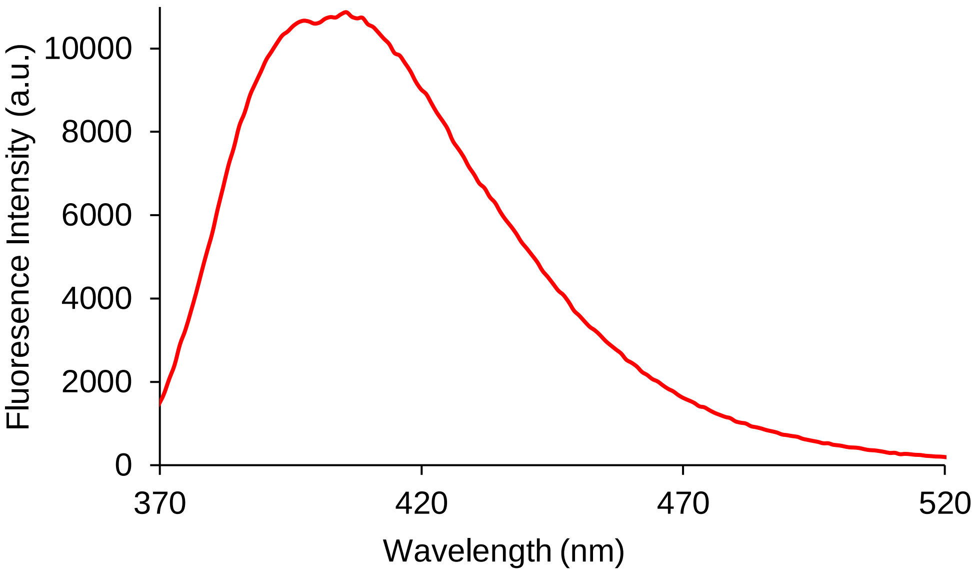

Absorption and Emission Spectra for 11d.

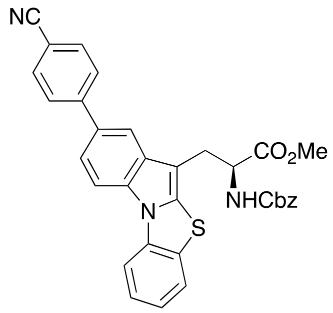

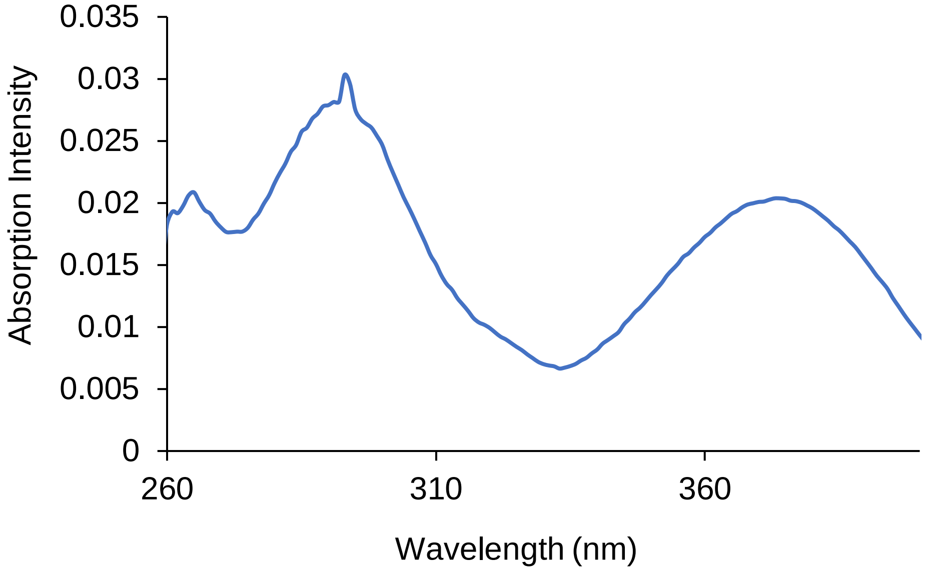

Excitation at 373 nm:

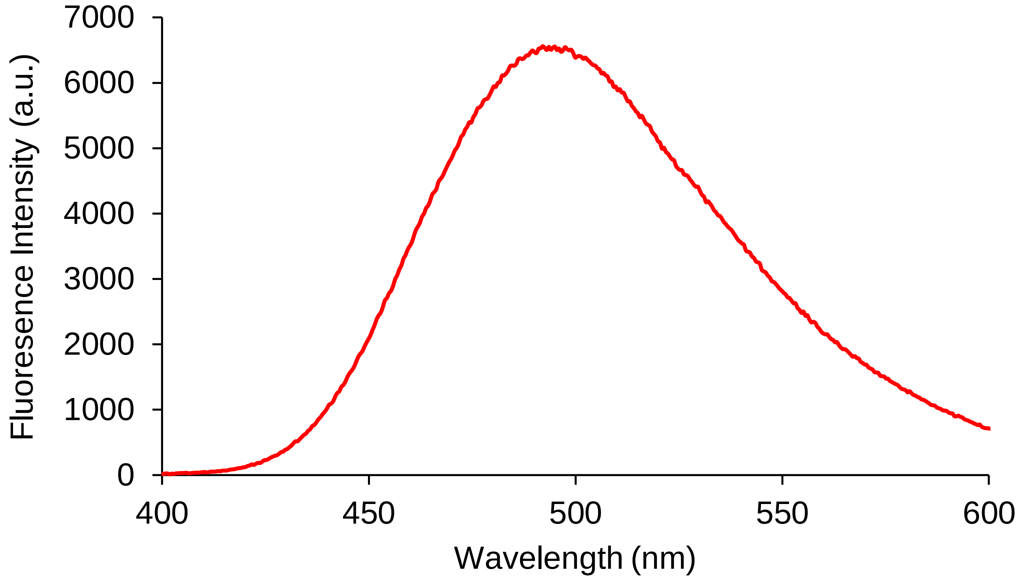

Absorption and Emission Spectra for 11e.

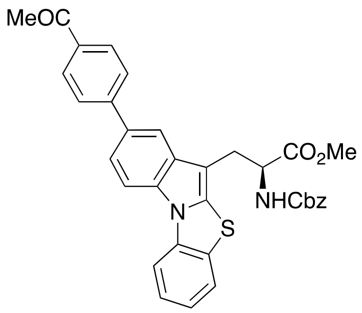

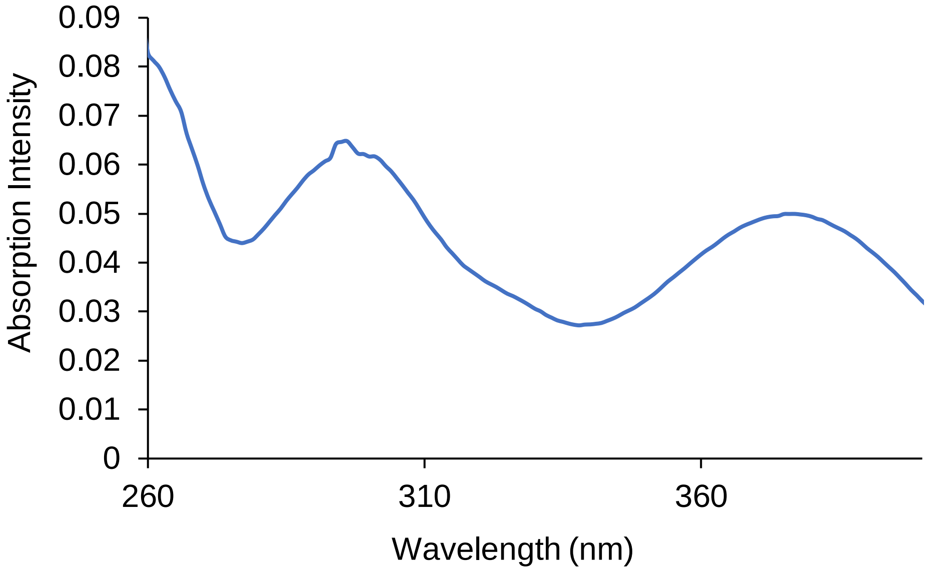

Excitation at 376 nm:

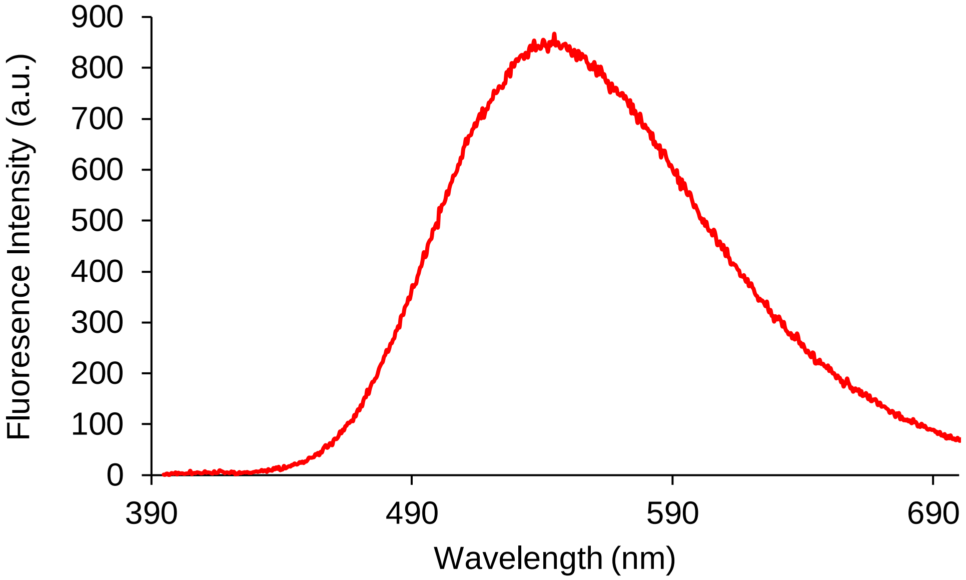

Absorption and Emission Spectra for 11f.

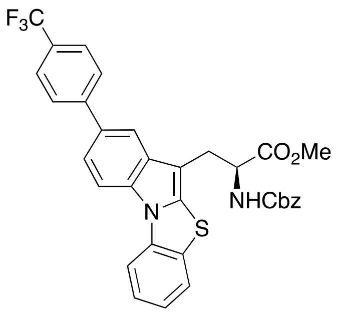

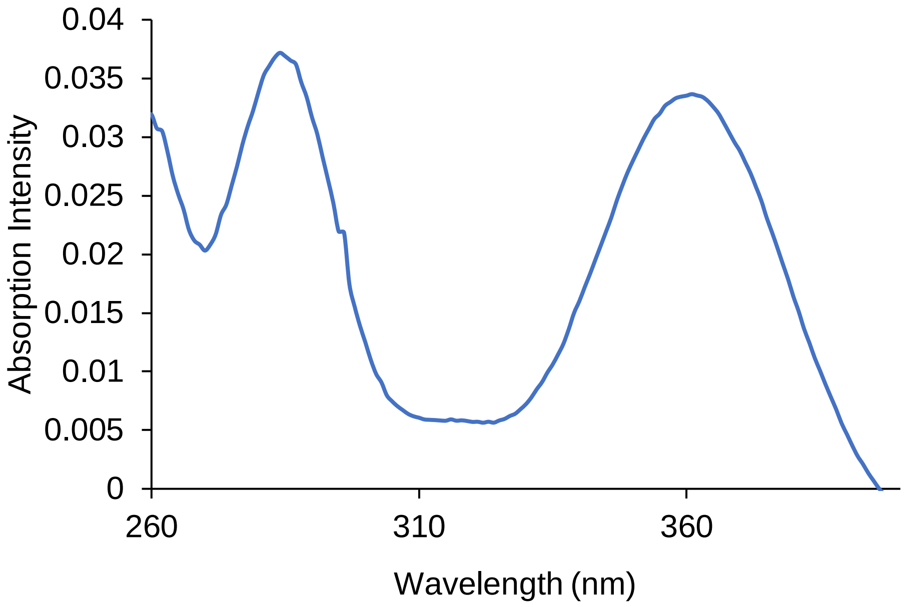

Excitation at 359 nm:

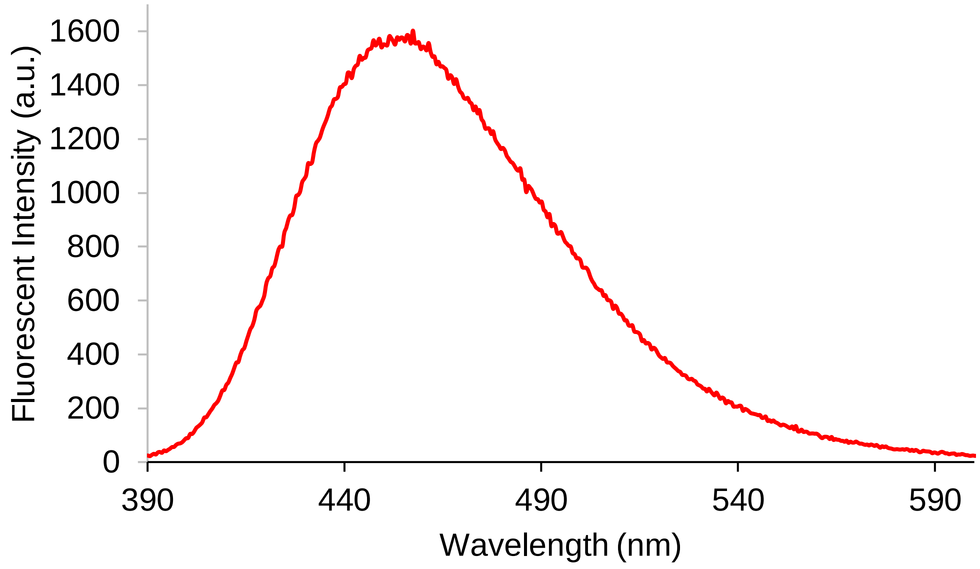

Absorption and Emission Spectra for 12a.

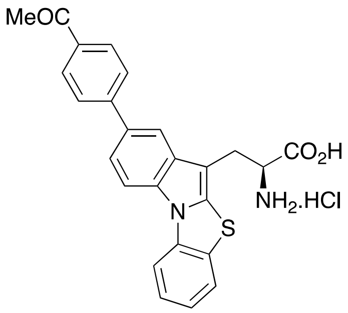

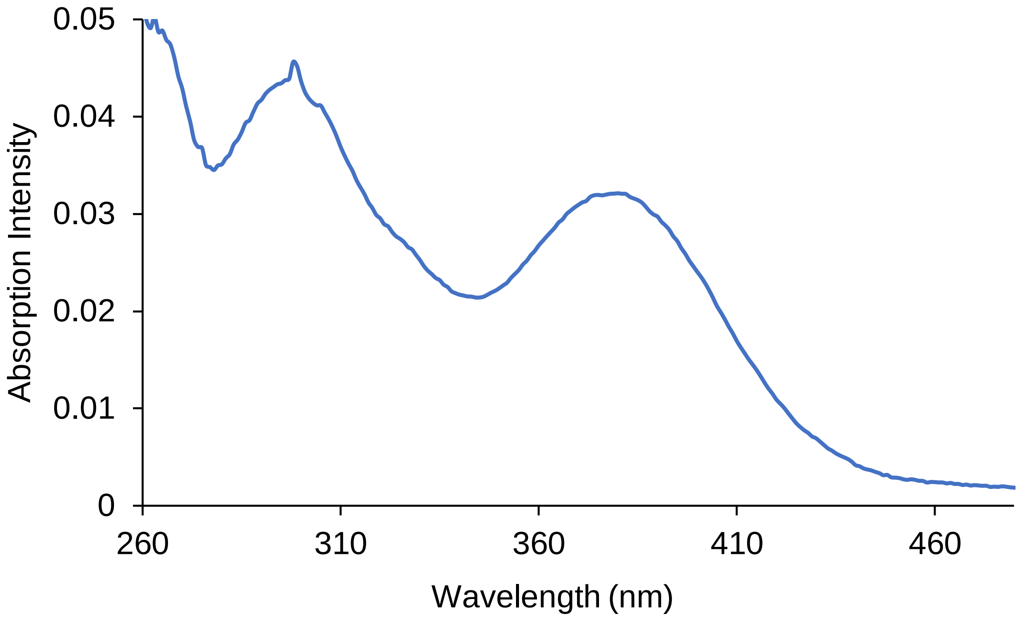

Excitation at 378 nm:

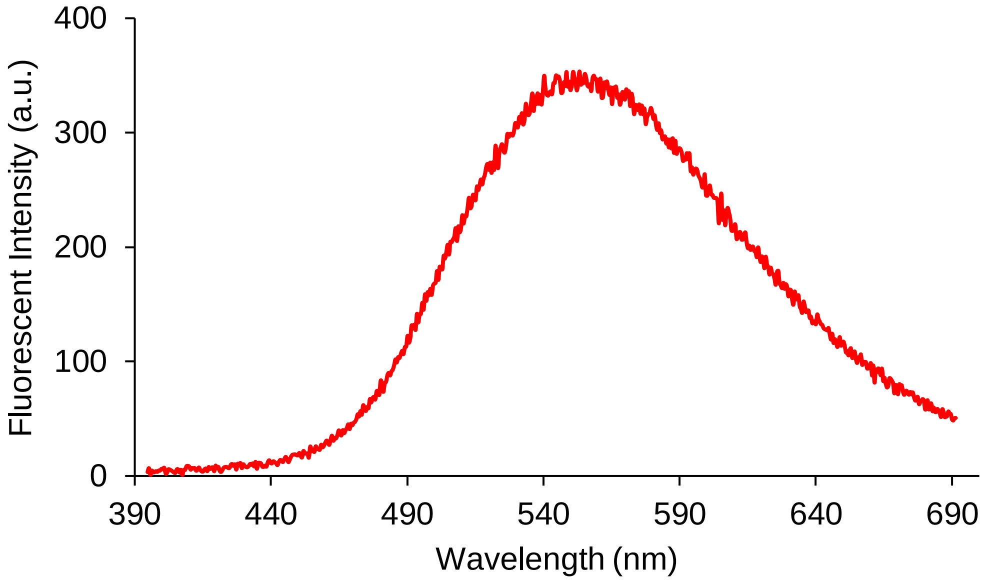

Absorption and Emission Spectra for 12b.

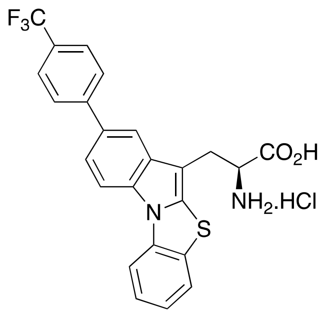

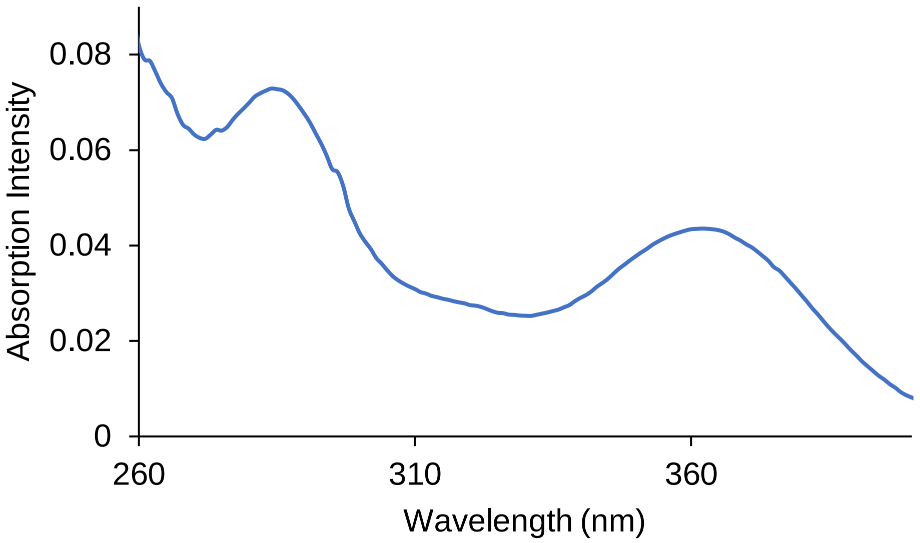

Excitation at 362 nm:

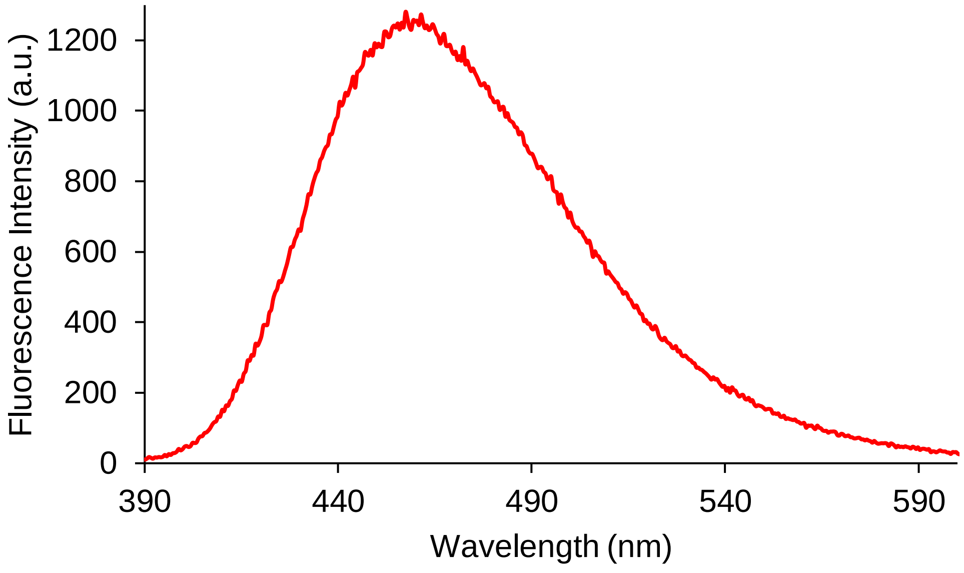

Solvatochromic Study for 12b.

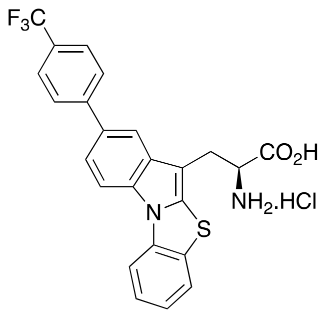

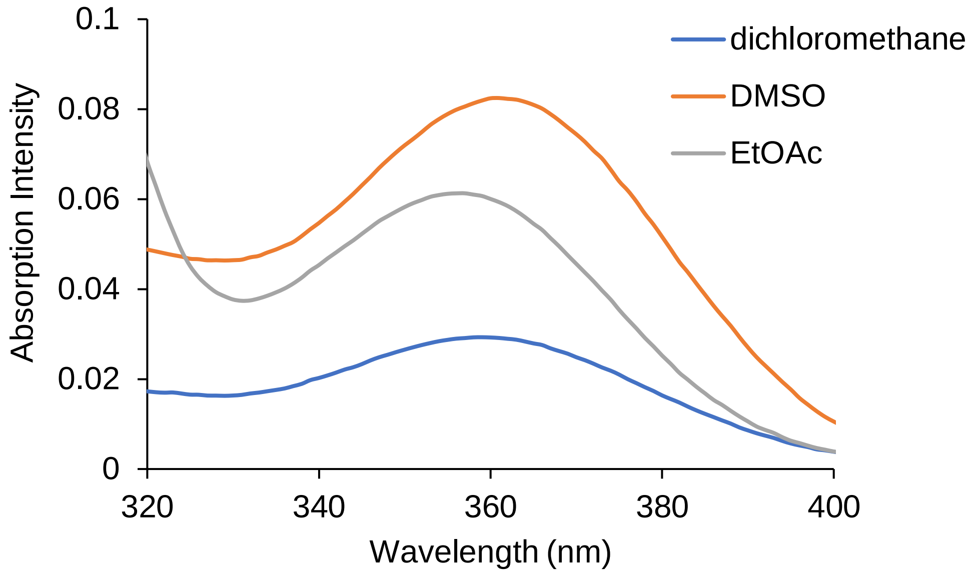

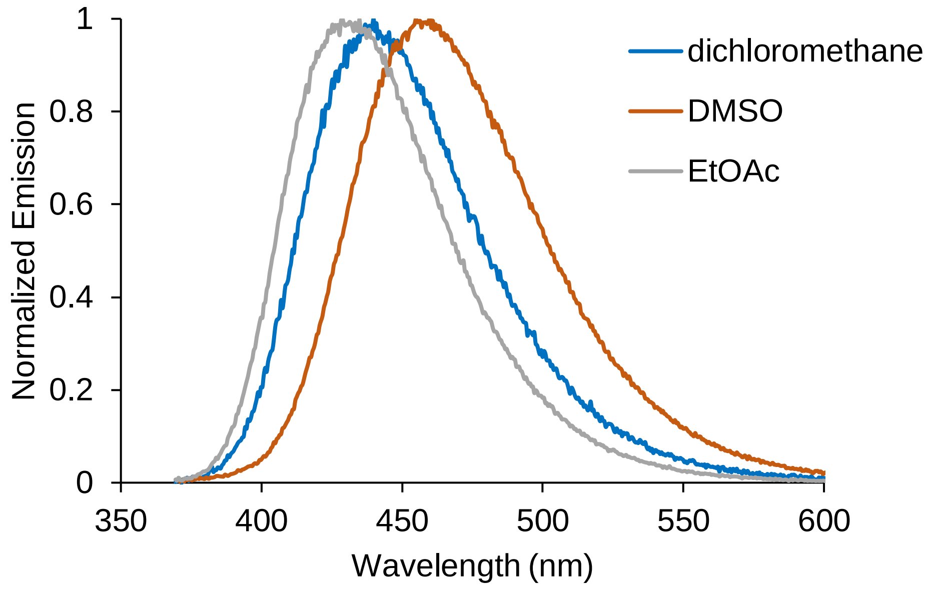

5. Two-Photon Calculations, Experimental Details and Spectra

Setup: A schematic of the two-photon spectroscopy setup can be found below. The excitation source is a broadband Ti:Sapphire laser with a repetition rate of 80 MHz (Vitara UBB, Coherent). The compressed pulses from the oscillator have a duration of 15 fs. For power dependence measurements, the laser was attenuated by a combination of 2 neutral density filters in series (Thorlabs) and a clean-up filter (Chroma, ET6651p) to obtain different excitation powers. For cross-section measurements, the laser was filtered with a bandpass filter (Thorlabs 700 nm, 10 nm FWHM or Thorlabs 800 nm, 10 nm FWHM) and 1 neutral density filter. For the cross-section measurement at 800 nm, the neutral density filter was changed to achieve different excitation powers; due to the power available at 700 nm, this measurement was made at a single laser power. To reduce reabsorption effects, the incident light was focused close to the cuvette wall nearest the objective lens. The light passes through a dichroic mirror (Semrock FF652-Di01) and is focused by an objective (Nikon Plan Fluor 10×) into a cuvette with the sample. The emitted light is collected by the same objective and reflected by the dichroic mirror onto a short-pass filter (Semrock, FF01-650/SP) to filter out any residual laser light. Subsequently it is focused by a lens (Edmund PCX UV 25×38, coating UV-VIS CTD TS) onto an optical fibre and guided into the spectrometer (AvaSpec ULS2048L-USB2 with the grating VA from Avantes). One-photon spectra were recorded on the same spectrometer to confirm that the same emission spectra were generated via one- and two-photon excitation; the excitation source was a fibre coupled 400 nm LED (Avantes, AVALIGHT-LED-400). Emission spectra were not corrected for the spectral response of the detector.

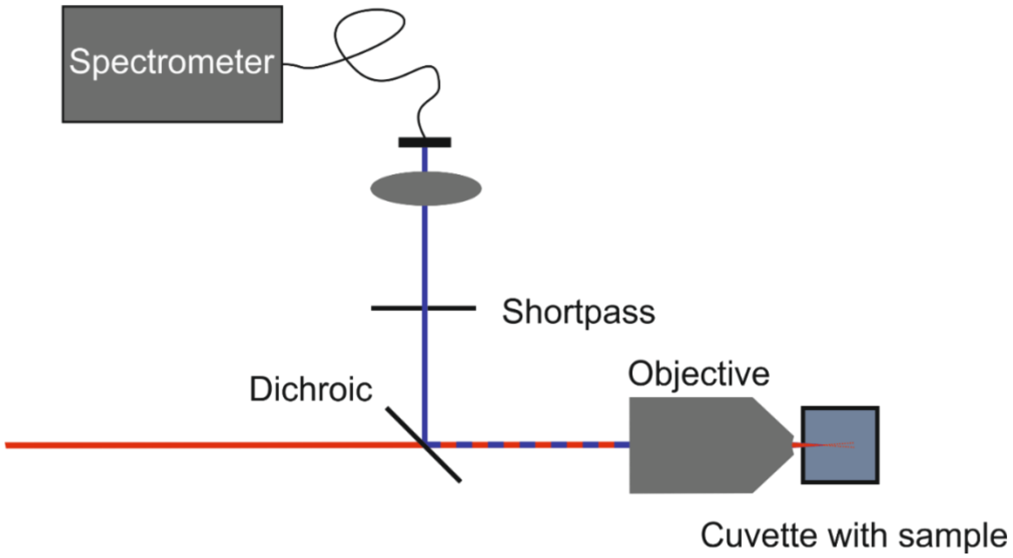

Power dependence:

To confirm two photon excitation of **12a** and **12b**, the emission intensity dependence with the excitation power was recorded. The results are shown below. The slope of the linear fit to the logarithmic plot was 1.92 ± 0.02 for **12a** and 1.92 ± 0.002 for **12b**, which is in good agreement with a two-photon excitation.

12a:

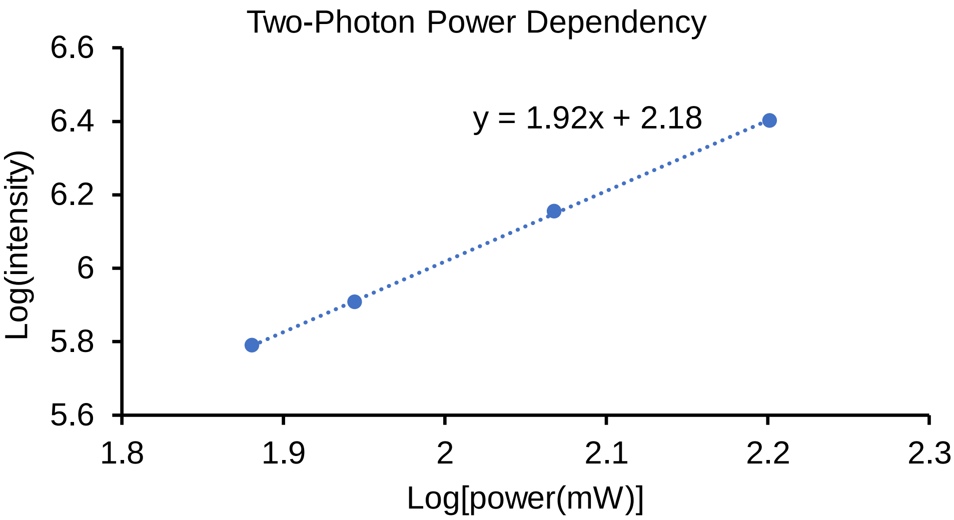

12b:

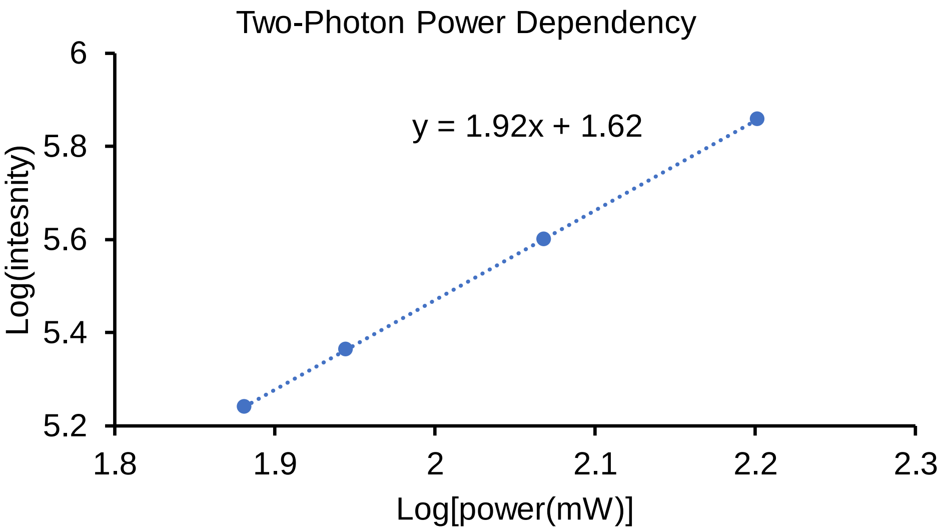

Two-photon cross sections:

The two-photon cross sections of the samples, σ_2^S, were calculated using the following equation by using a standard with a known cross section, σ_2^R:

$$\frac{\sigma_2^S \phi^S}{\sigma_2^R \phi^R} = \frac{\eta^R (n_{\lambda emission}^S)^2 n_{\lambda excitation}^R C^R F^S \langle P^R \rangle^2}{\eta^S (n_{\lambda emission}^R)^2 n_{\lambda excitation}^S C^S F^R \langle P^S \rangle^2}$$

Where ϕ is the quantum yield of the fluorescence, η accounts for the wavelength dependence of the detection efficiency, n is the refractive index of the solvent C is the concentration, F is the integrated fluorescence signal and $\langle P \rangle$ is the excitation power. The superscripts R and S denote the reference and sample respectively.

Simplifying by $\eta^R = \eta^S$ and $n_{excitation} = n_{emission} = n$, the equation can be rewritten as:

$$\frac{\sigma_2^S \phi^S}{\sigma_2^R \phi^R} = \frac{n^S C^R M^S}{n^R C^S M^R}$$

Where $M = \frac{F}{\langle P \rangle^2}$, the gradient of the integrated fluorescence intensity plotted against the square of the excitation power.

The standard used was rhodamine B with a quantum yield of 0.7[5] and a cross section of 120 ± 18 GM at 800 nm and 240 ± 36 GM at 700 nm.[6] $n_{methanol}$: 1.331, n_{DMSO}: 1.477. Sample concentrations were 2.3 μM for rhodamine B, 55 μM for **12a** and 60 μM for **12b**.

6. Time-Correlated Single-Photon Counting (TCSPC)

Fluorescence lifetime measurements were recorded for **12a** (5.6 μM) and **12b** (5.5 μM) in DMSO using a FluoTime 300 fluorescence spectrometer (PicoQuant, Berlin, Germany). The excitation light was provided by a Fianium WhiteLase super-continuum laser (NKT Photonics, Birkerød, Denmark) and passed through a SuperChrome filter (NKT Photonics, Birkerød, Denmark) before arriving at the sample holder. The FWHM of the instrument response function was ≈ 120 ps at 10 MHz rep rate. The excitation wavelength for both samples were 390 nm. The emission wavelength was 545 nm for **12a** and 461 nm for **12b**. The excitation bandpass was 10 nm, while the emission bandpass was 2.7 nm. All fluorescence measurements were carried out under magic-angle conditions to avoid fluorescence polarization artefacts. The time/channel was 8 ps. The measurements were recorded until they reached five thousand counts in the peak channel. The fluorescence decays were best fit to a mono-exponential decay function. The fitting was carried out using the FluoFit data analysis software (PicoQuant,

Berlin, Germany). Decays were fitted by iterative re-convolution, assuming a mono-exponential function, given in eq 1.

$$I(t) = I_0 \exp\left(-\frac{t}{\tau}\right) \qquad (1)$$

where I is the fluorescence intensity as a function of time, t, (normalized to the intensity at $t = 0$) and τ is the fluorescence lifetime. The χ^2 value was 0.979 ± 0.01 for **12a** and 0.975 ± 0.004 for **12b**. The residuals were randomly distributed around zero.

Fluorescence decay for **12a**:

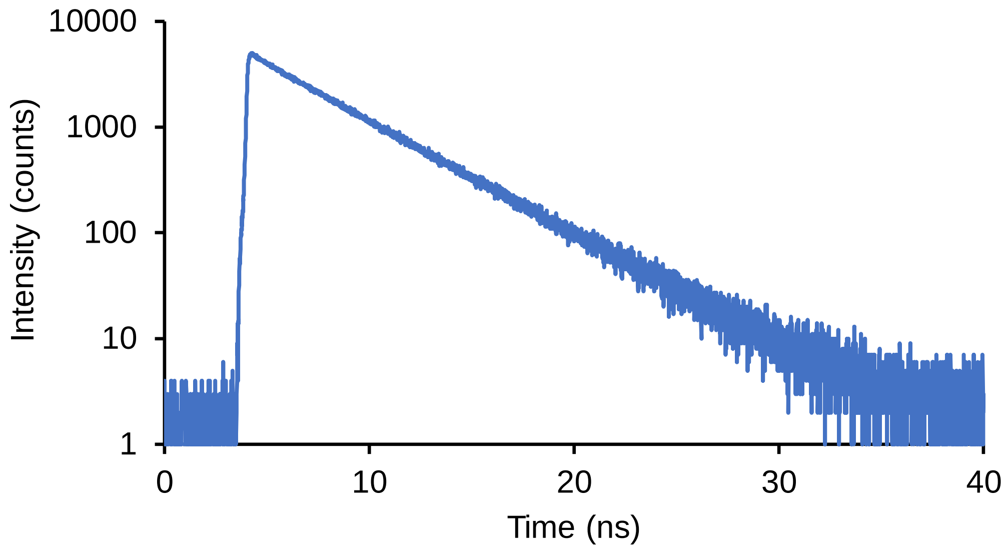

Fluorescence decay for **12b**:

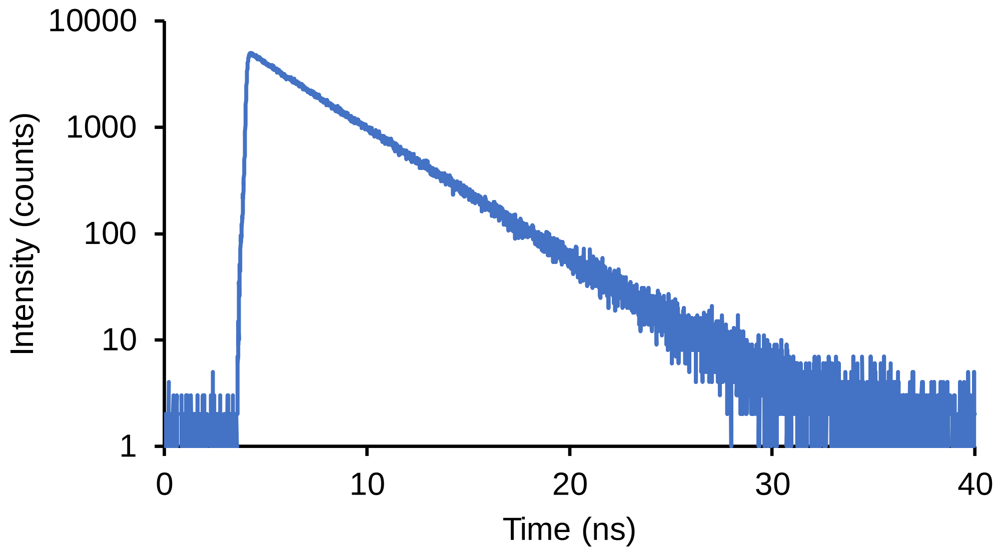

The fluorescence lifetimes of **12a** and **12b** were found to be 4.04 ± 0.007 ns and 3.54 ± 0.002 ns, respectively.

7. References

1. Dodds, A. C.; Sutherland, A. Regioselective C–H Thioarylation of Electron-Rich Arenes by Iron(III) Triflimide Catalysis. *J. Org. Chem*. **2021**, *86*, 5922–5932.
2. Williams, A. T. R.; Winfield, S. A.; Miller, J. N. Relative Fluorescence Quantum Yields Using a Computer-controlled Luminescence Spectrometer. *Analyst* **1983**, *108*, 1067–1071.
3. Kandukuri, S. R.; Schiffner, J. A.; Oestreich, M. Aerobic Palladium(II)-Catalyzed 5-*endo-trig* Cyclization: An Entry into the Diastereoselective C-2 Alkenylation of Indoles with Tri- and Tetrasubstituted Double Bonds. *Angew. Chem. Int. Ed.* **2012**, *51*, 1265–1269.
4. Höck, S.; Marti, R.; Riedl, R.; Simeunovic, M. Thermal Cleavage of the Fmoc Protection Group. *Chimia* **2010**, *64*, 200–202.
5. Crosby, G. A.; Demas, J. N. Measurement of Photoluminescence Quantum Yields. *J. Phys Chem.* **1971**, *75*, 991–1024.
6. Makarov, N. S.; Drobizhev, M.; Rebane, A. Two-Photon Absorption Standards in the 550–1600 nm Excitation Wavelength Range. *Opt. Express* **2008**, *16*, 4029–4047.

8. ^{1}H and ^{13}C NMR Spectra for all Novel Compounds

^{1}H NMR (400 MHz, $CDCl_3$)

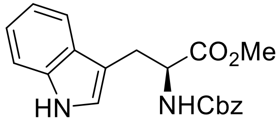

6

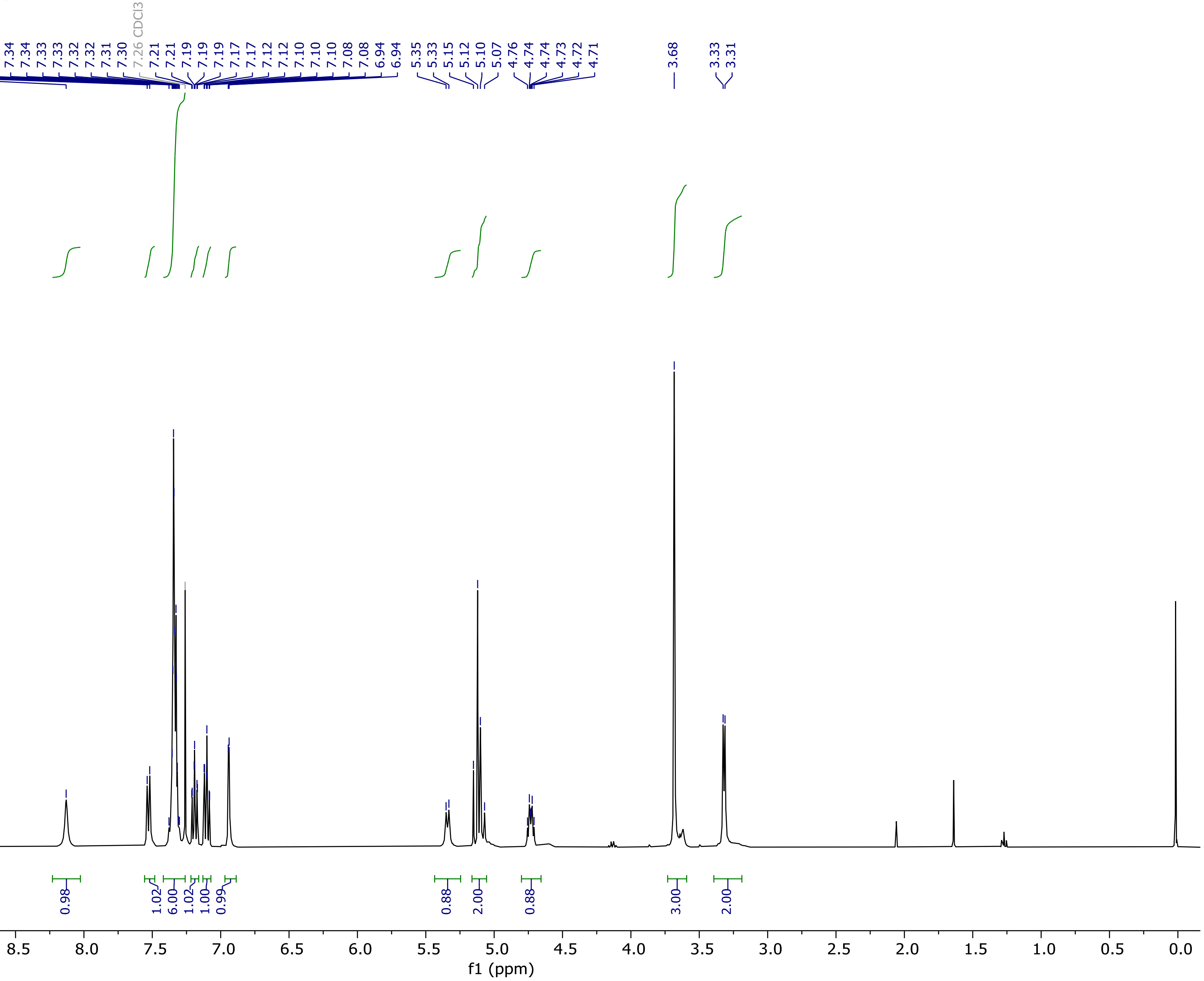

$^{13}C\{^1H\}$ NMR (101 MHz, $CDCl_3$)

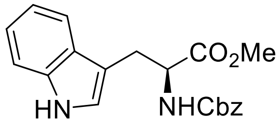

6

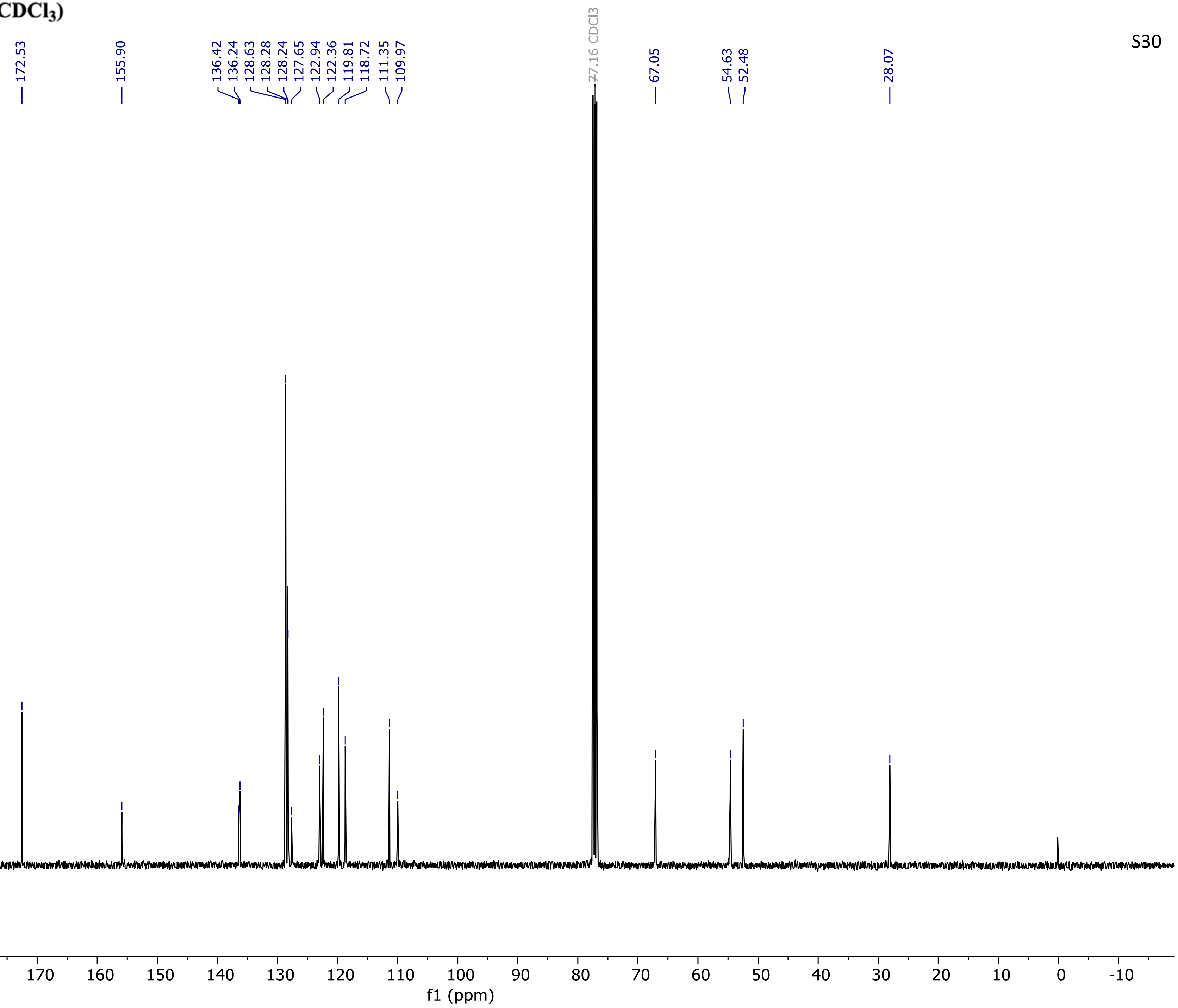

^{1}H NMR (400 MHz, $CDCl_3$)

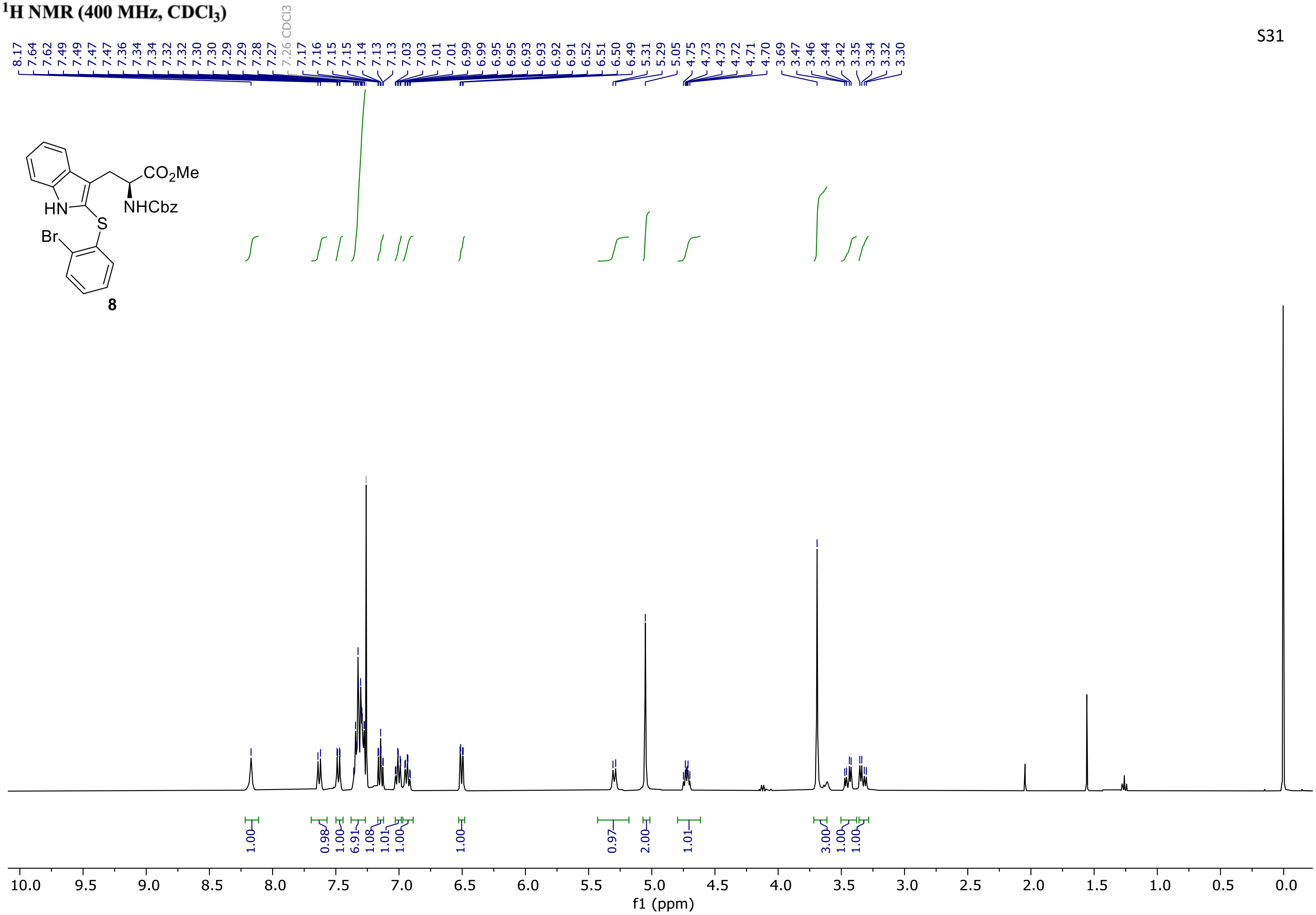

$^{13}C\{^1H\}$ NMR (101 MHz, $CDCl_3$)

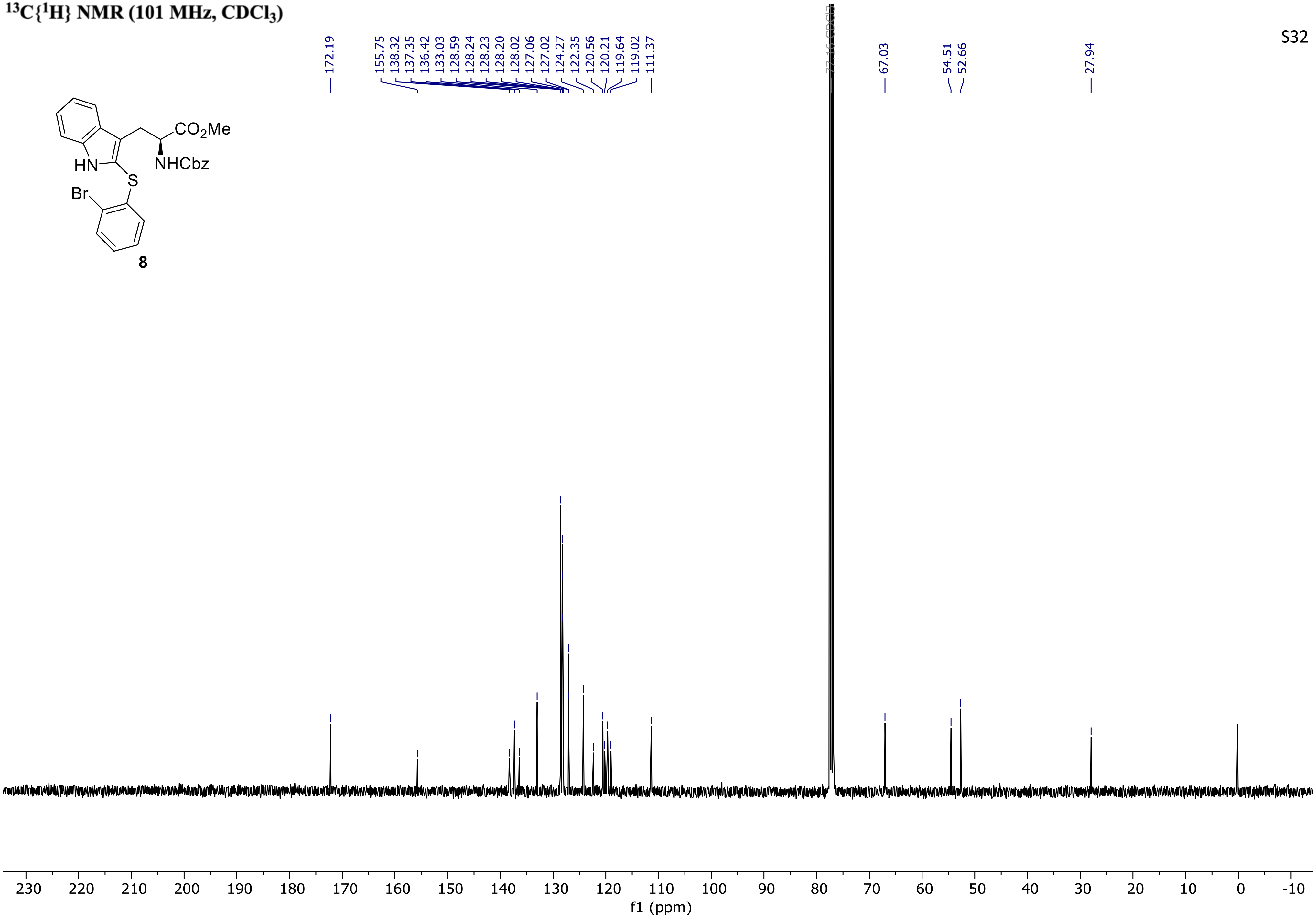

^1H NMR (400 MHz, $CDCl_3$)

9

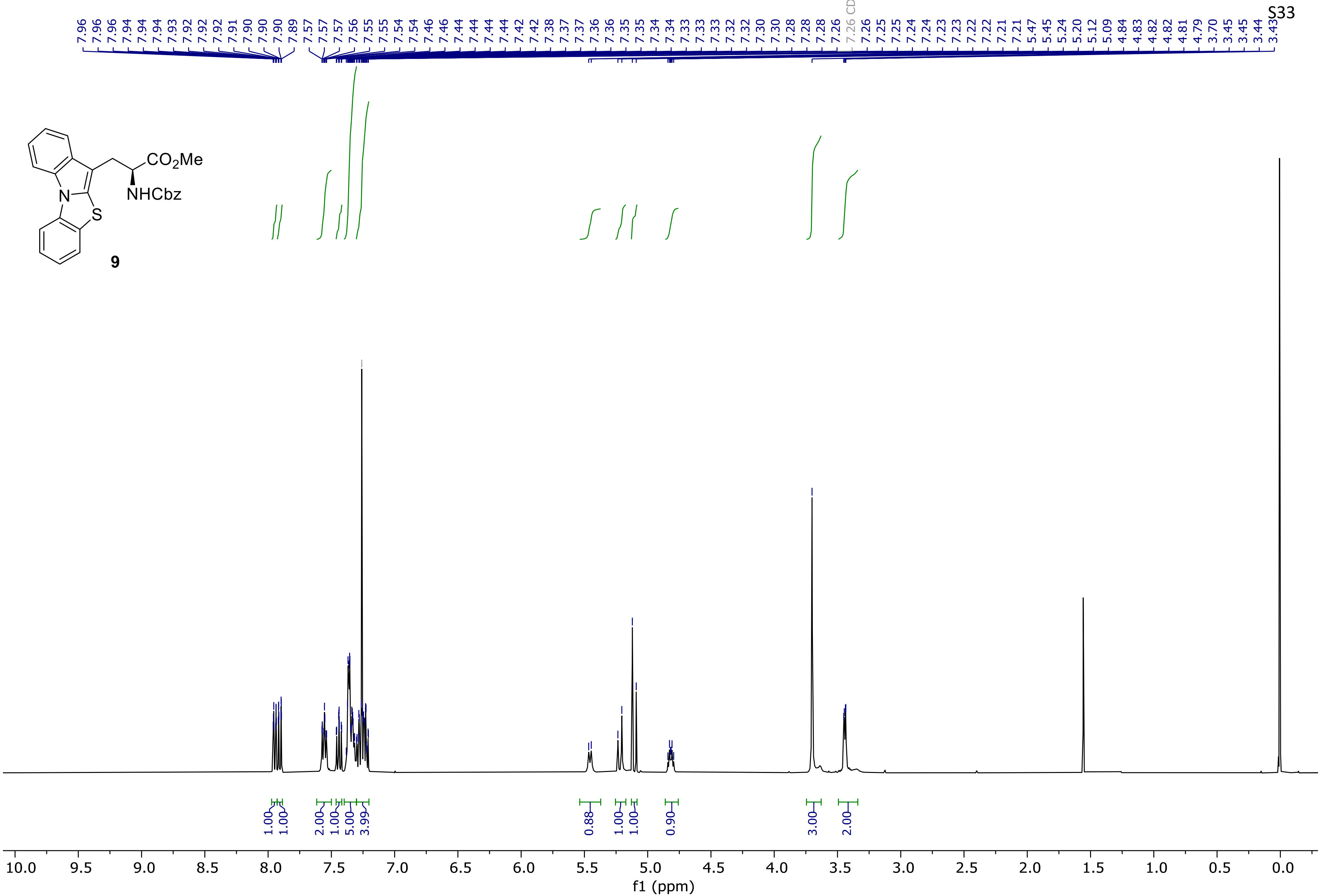

$^{13}C\{^{1}H\}$ NMR (101 MHz, $CDCl_3$)

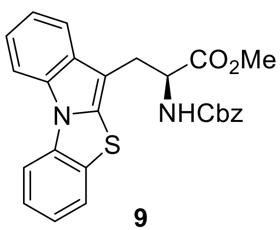

9

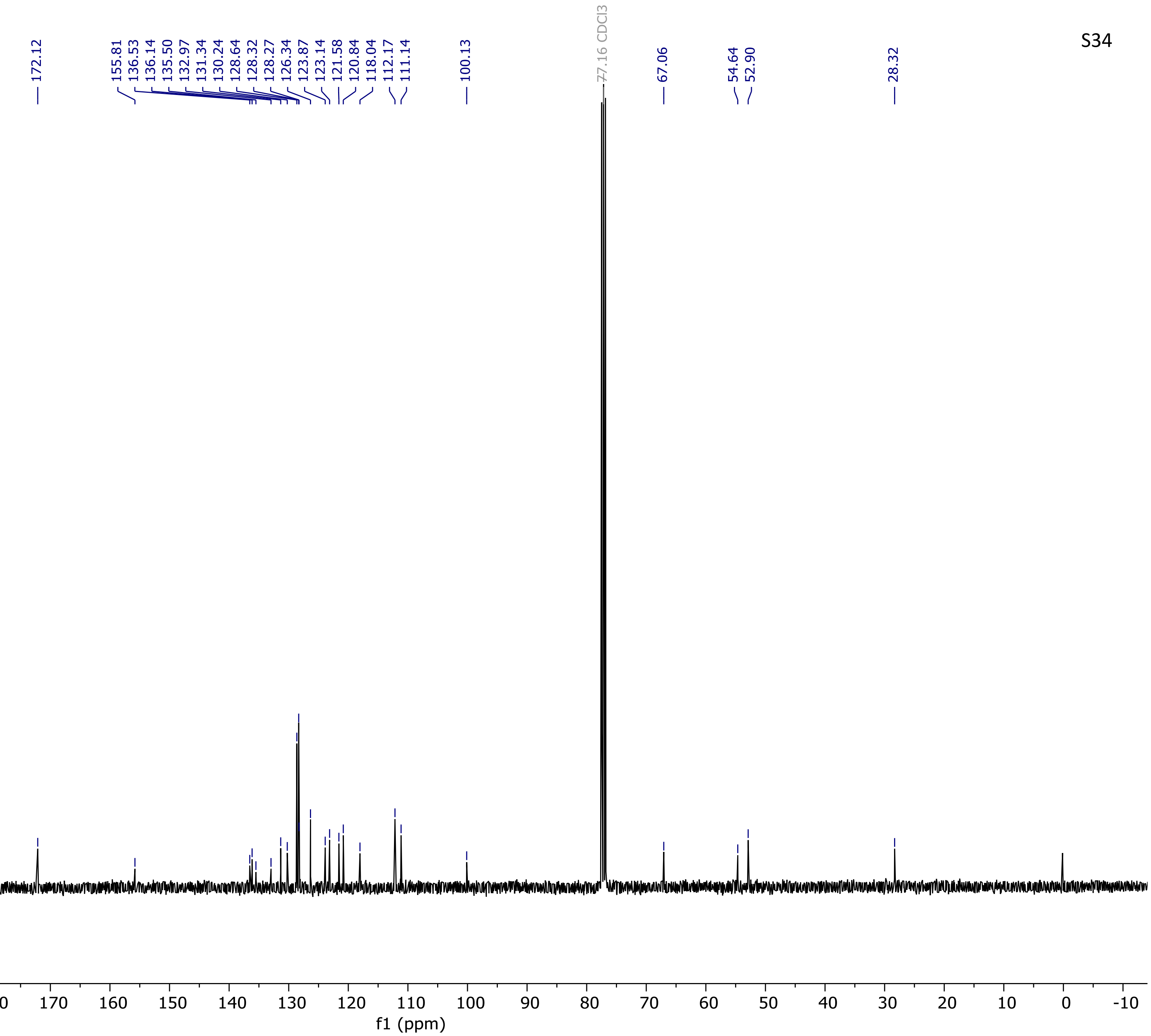

^{1}H NMR (400 MHz, $CDCl_3$)

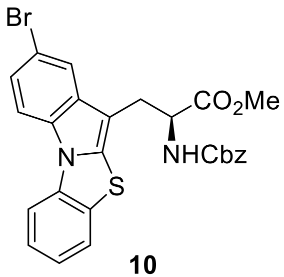

10

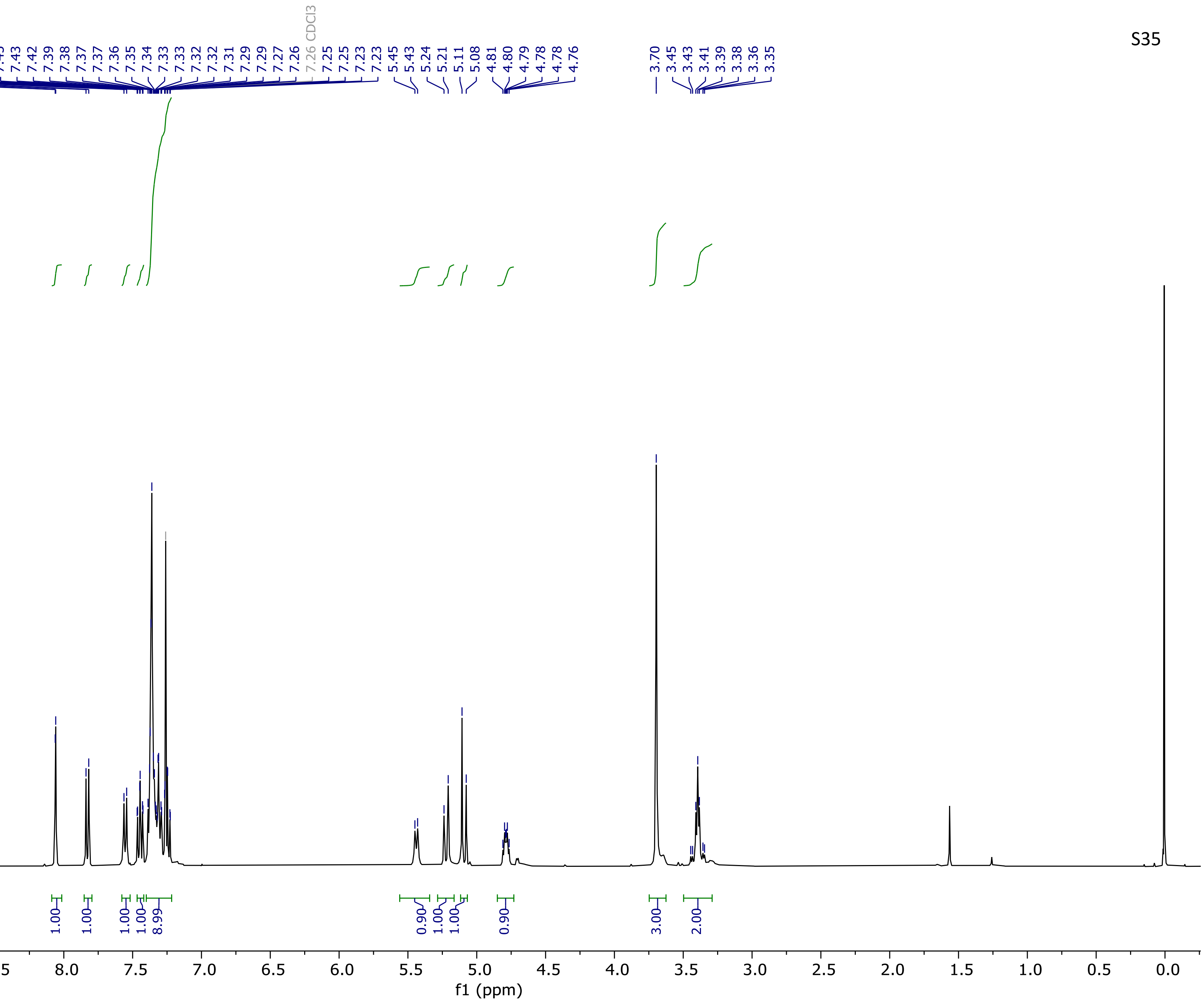

$^{13}C\{^1H\}$ NMR (101 MHz, $CDCl_3$)

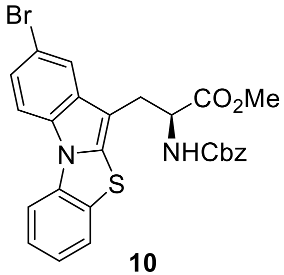

10

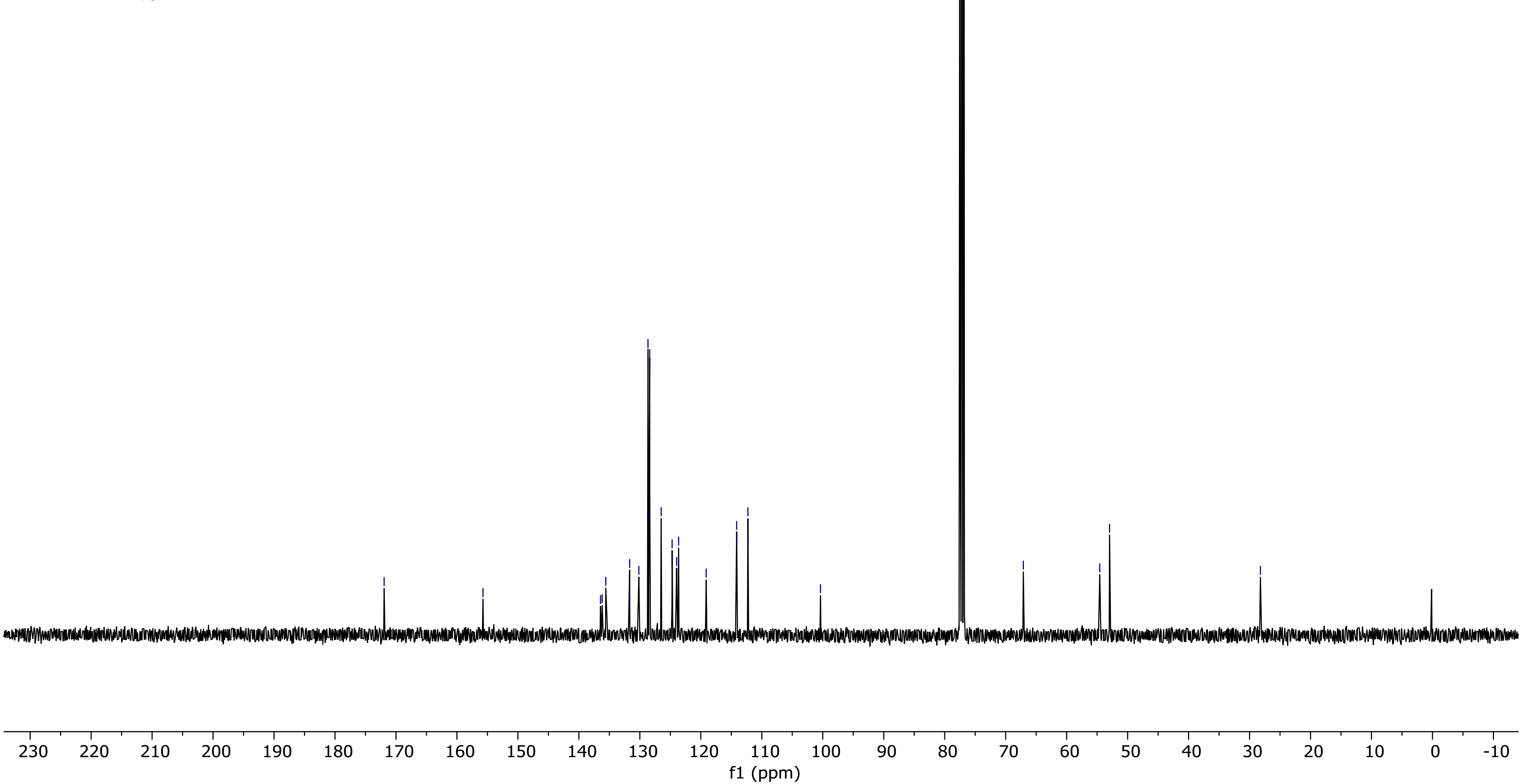

^{1}H NMR (400 MHz, $CDCl_3$)

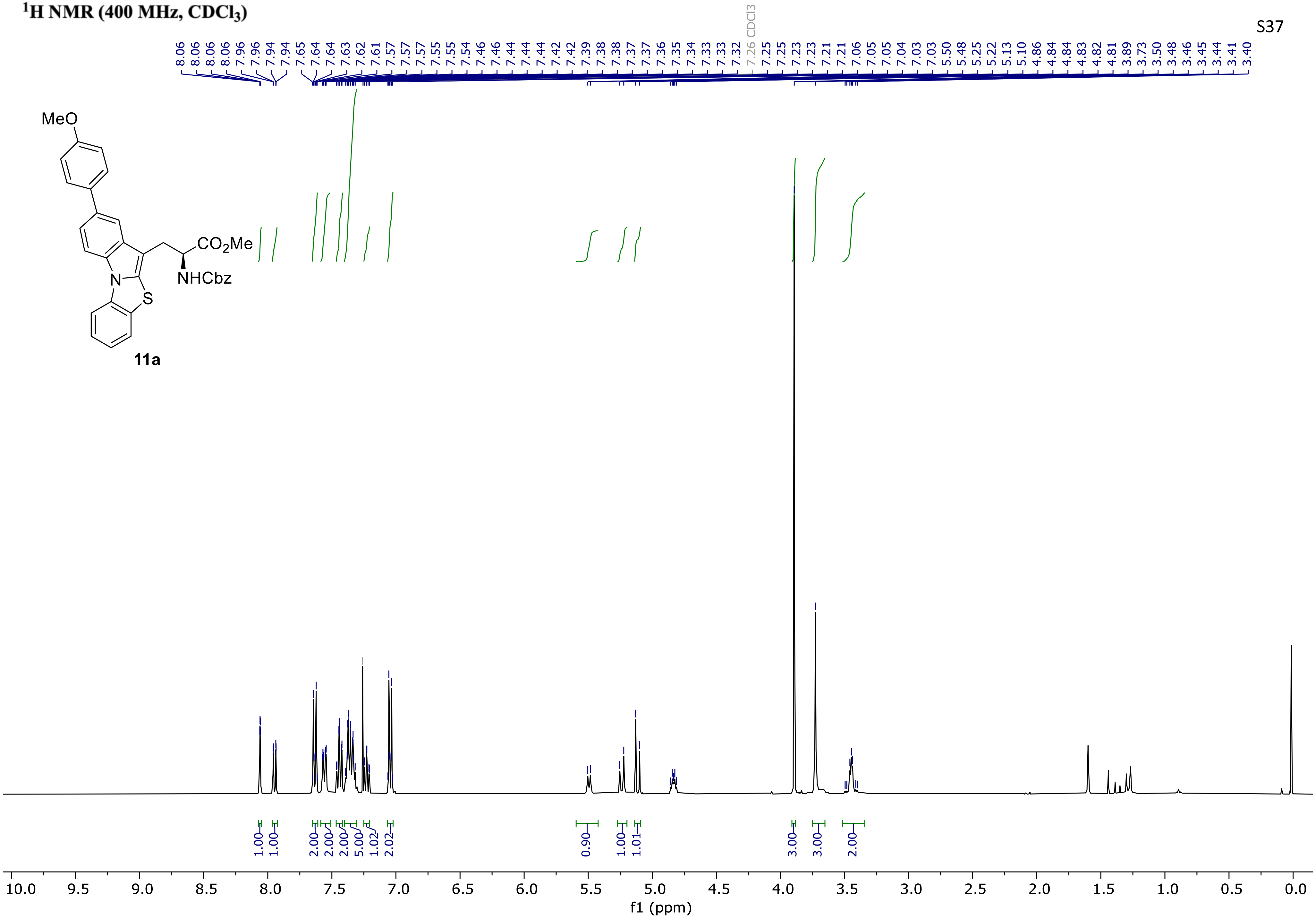

$^{13}C\{^{1}H\}$ NMR (101 MHz, $CDCl_3$)

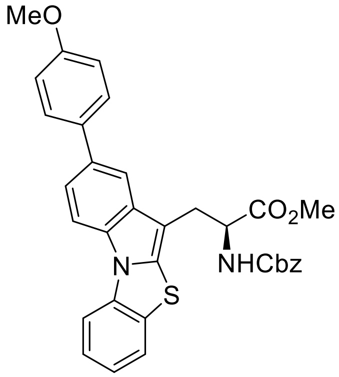

11a

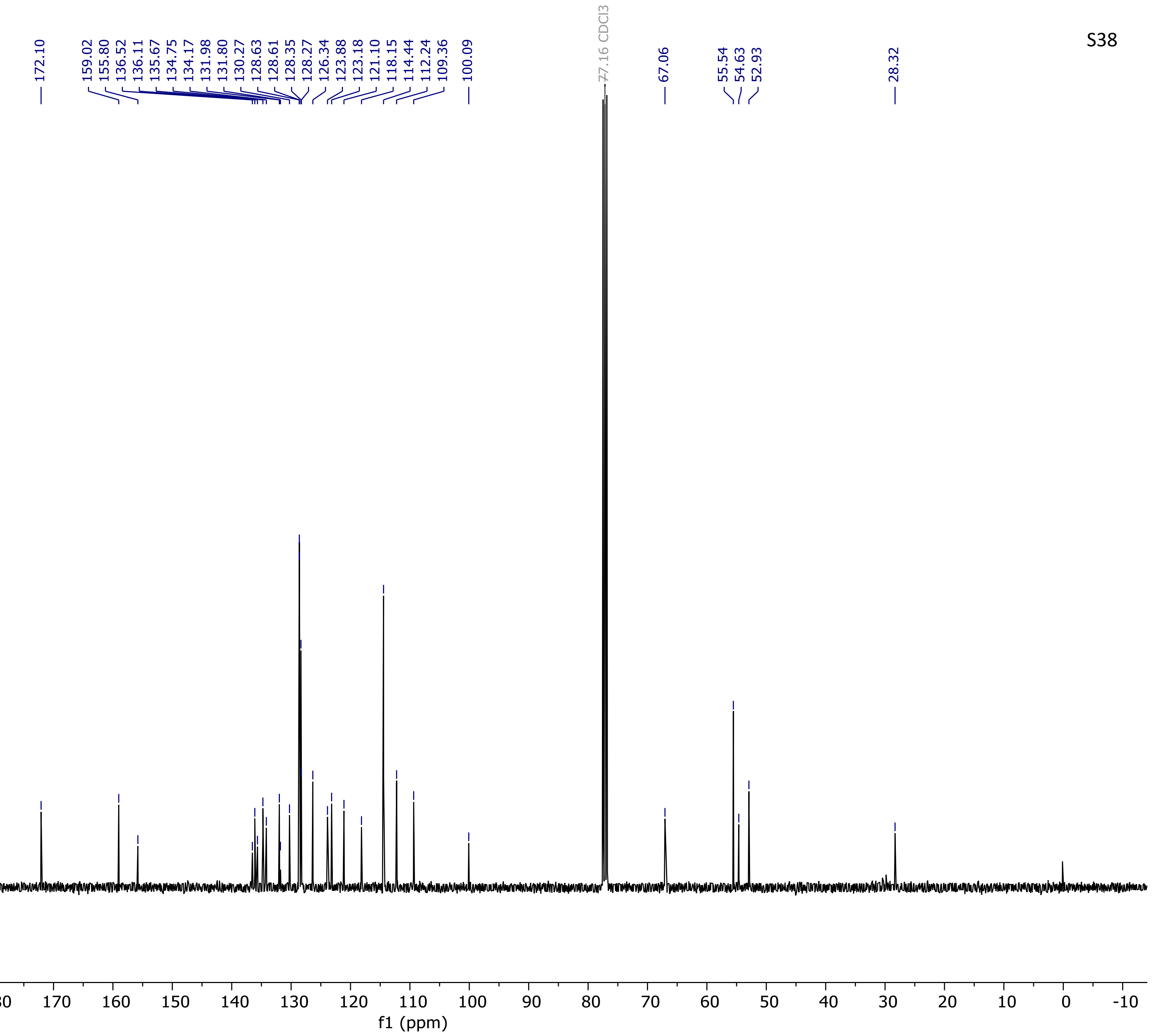

^{1}H NMR (400 MHz, $CDCl_3$)

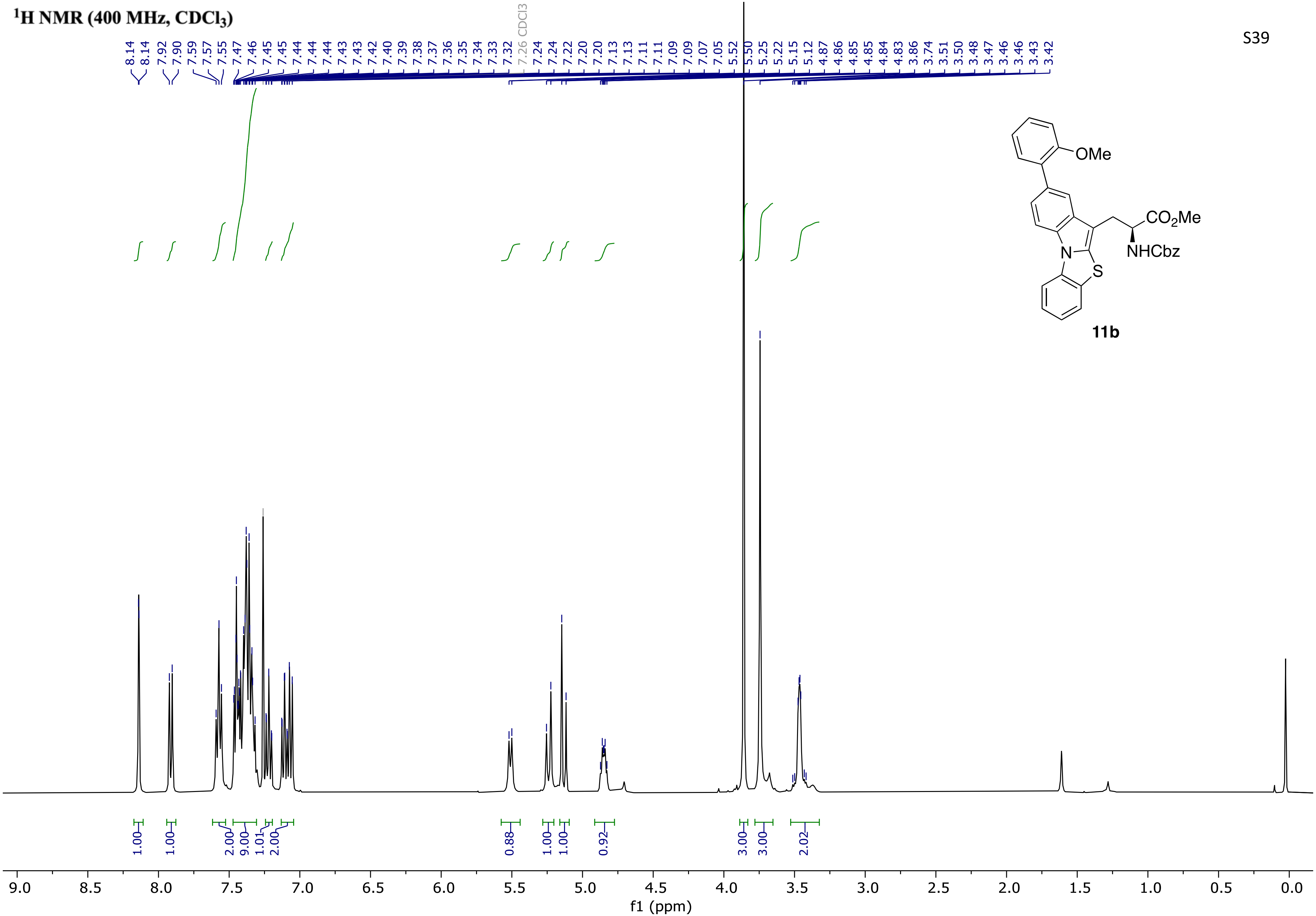

$^{13}C\{^1H\}$ NMR (101 MHz, $CDCl_3$)

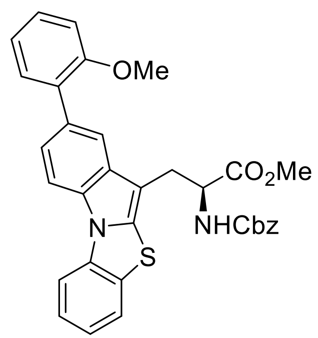

11b

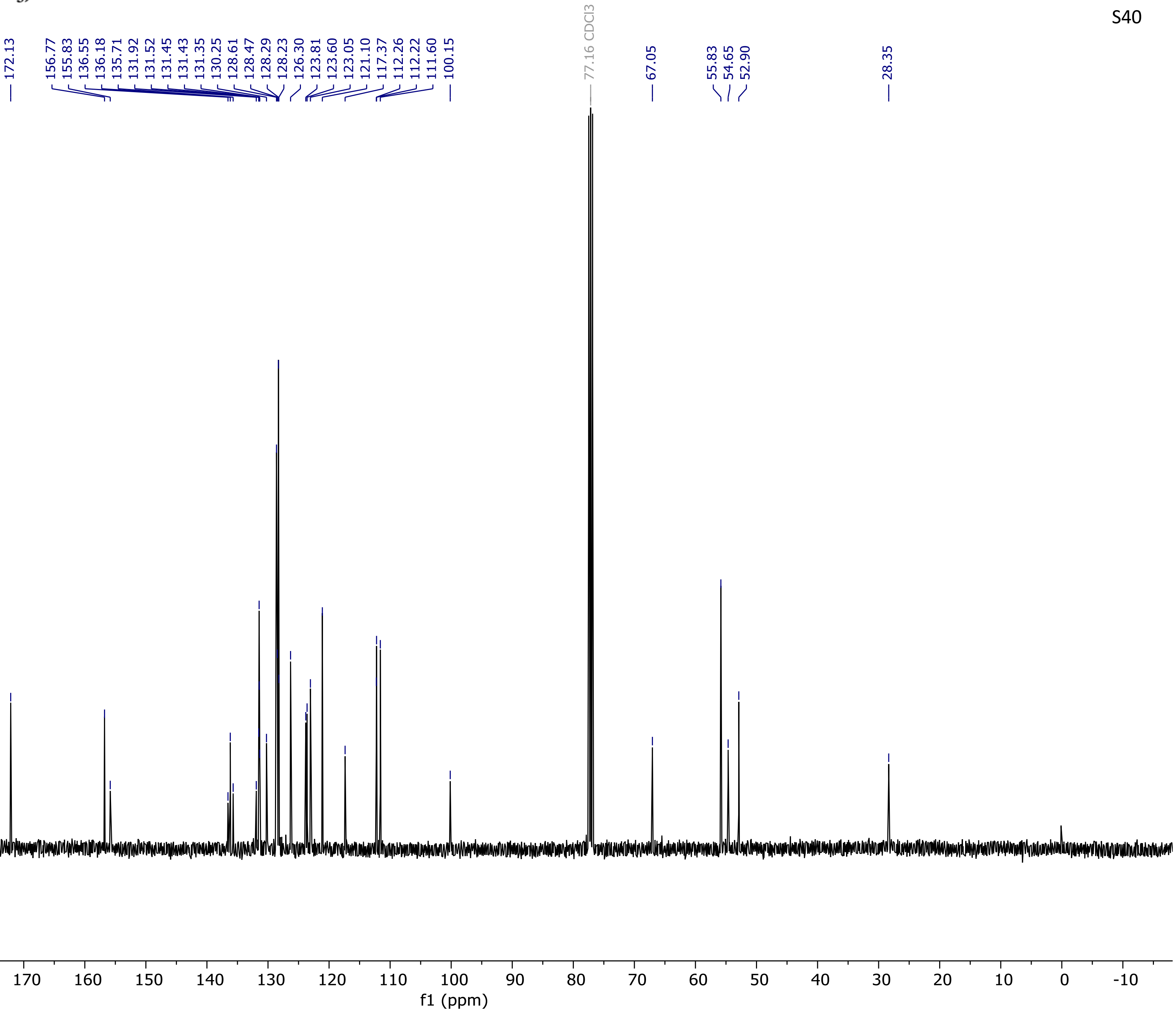

^{1}H NMR (400 MHz, $CDCl_3$)

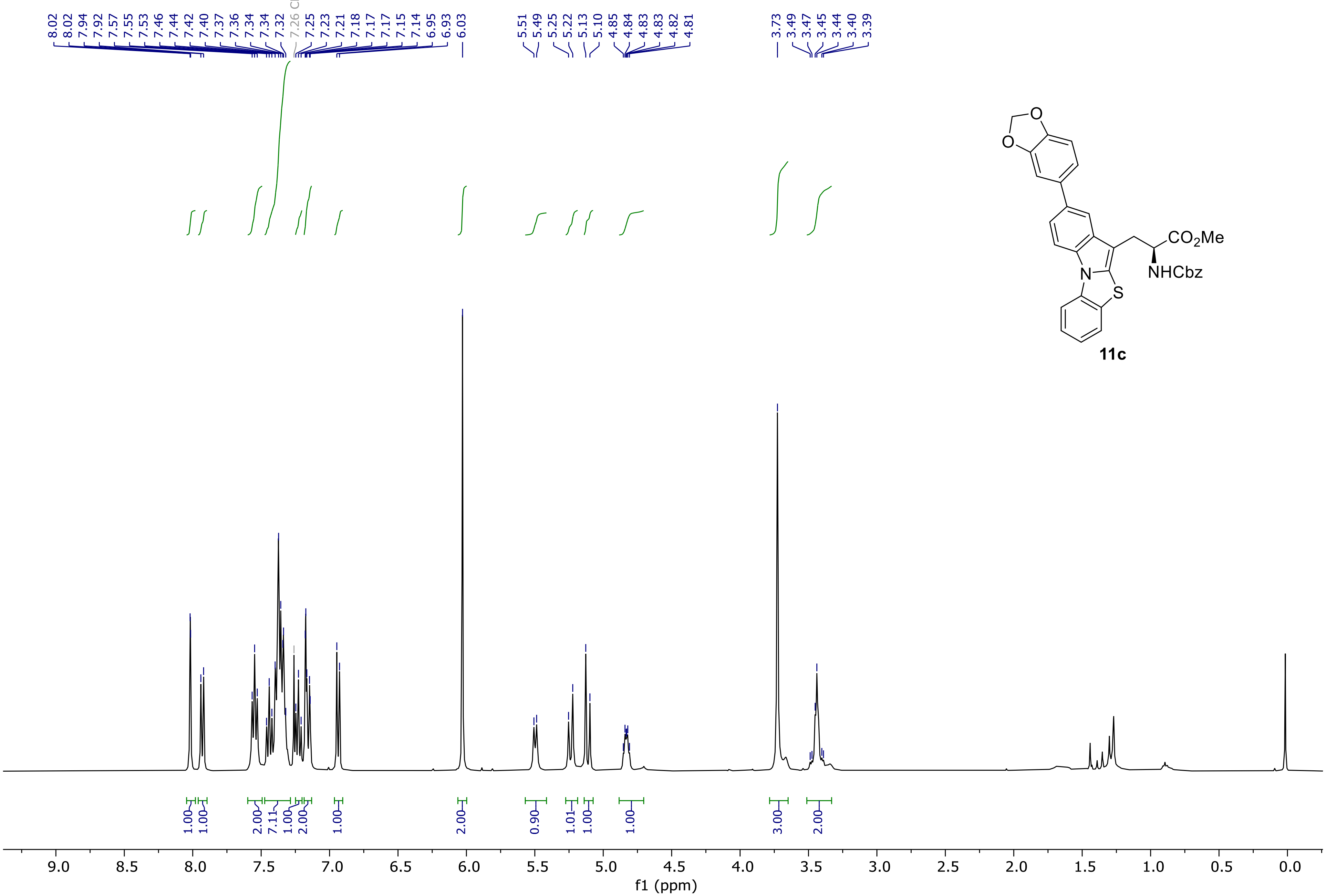

11c

$^{13}C\{^{1}H\}$ NMR (101 MHz, CDCl$_3$)

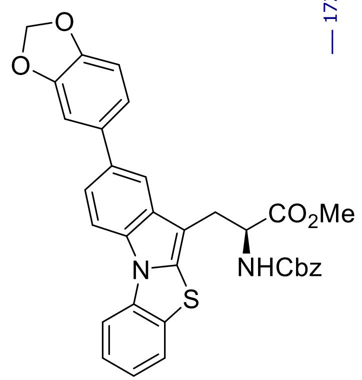

11c

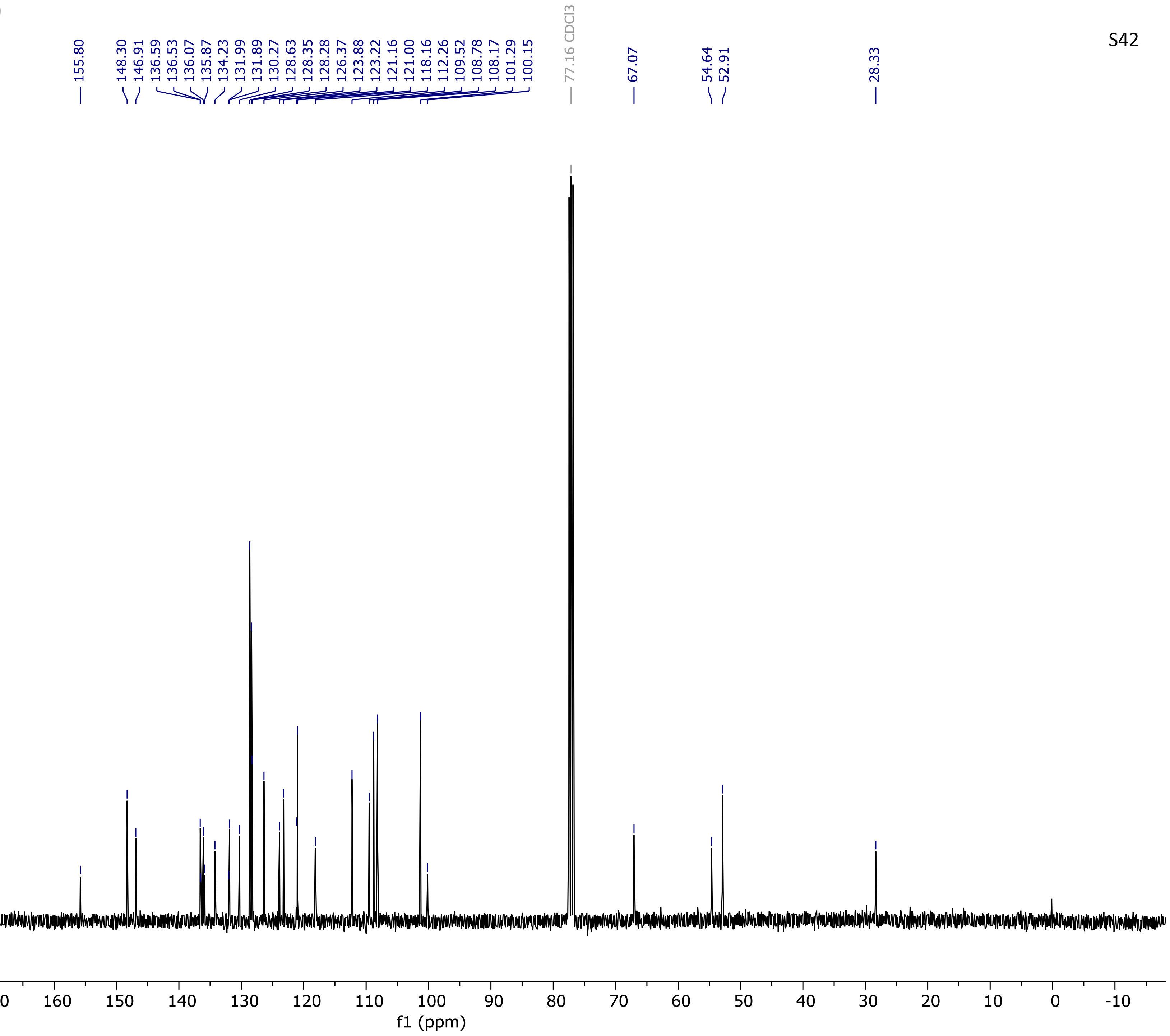

^{1}H NMR (400 MHz, $CDCl_3$)

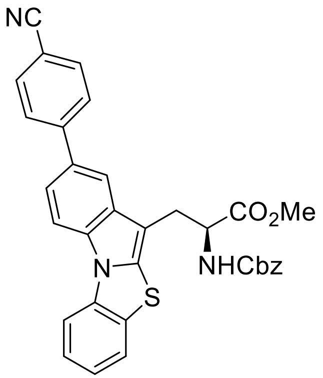

11d

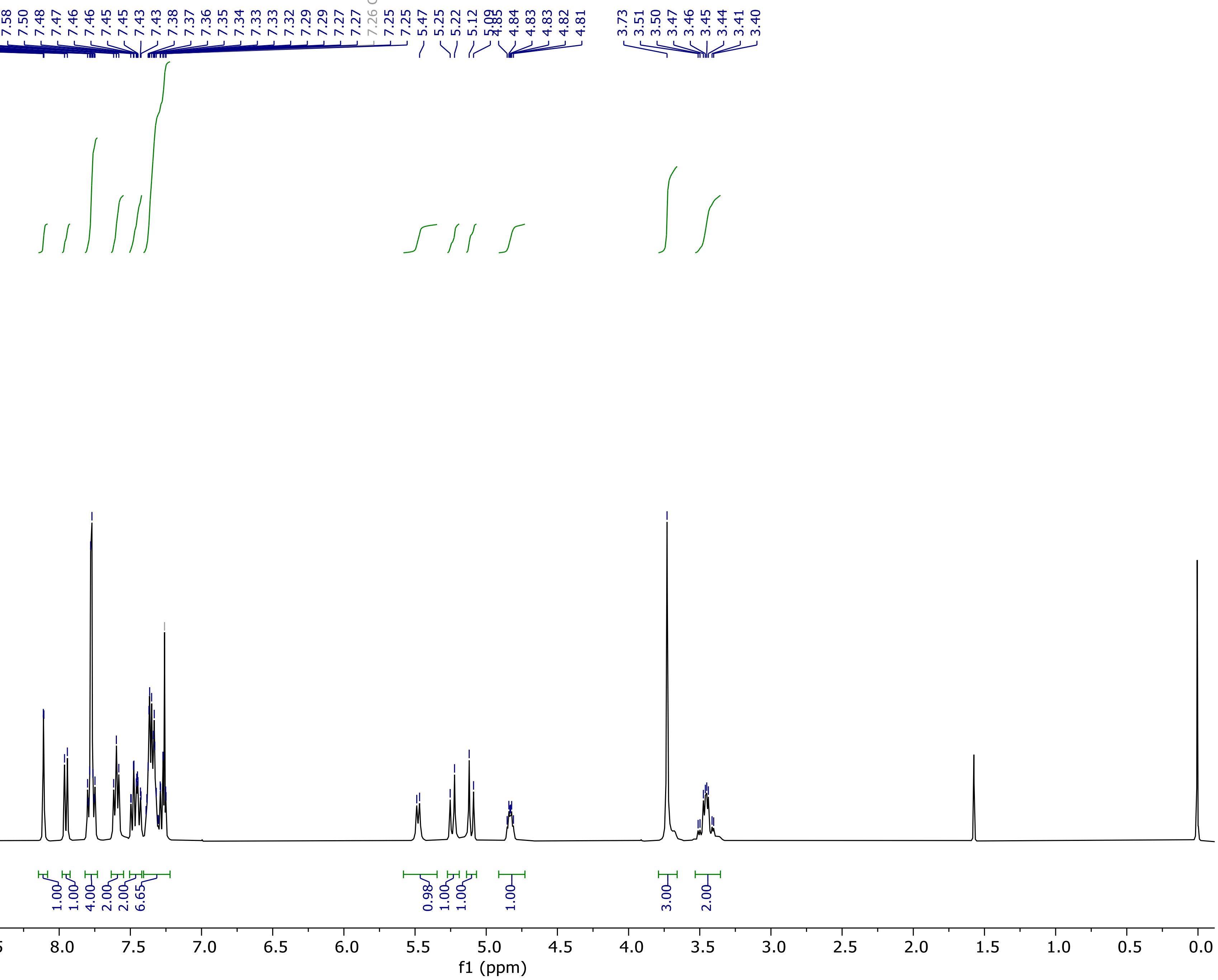

$^{13}C\{^{1}H\}$ NMR (101 MHz, $CDCl_3$)

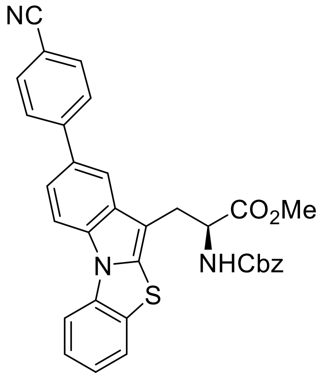

11d

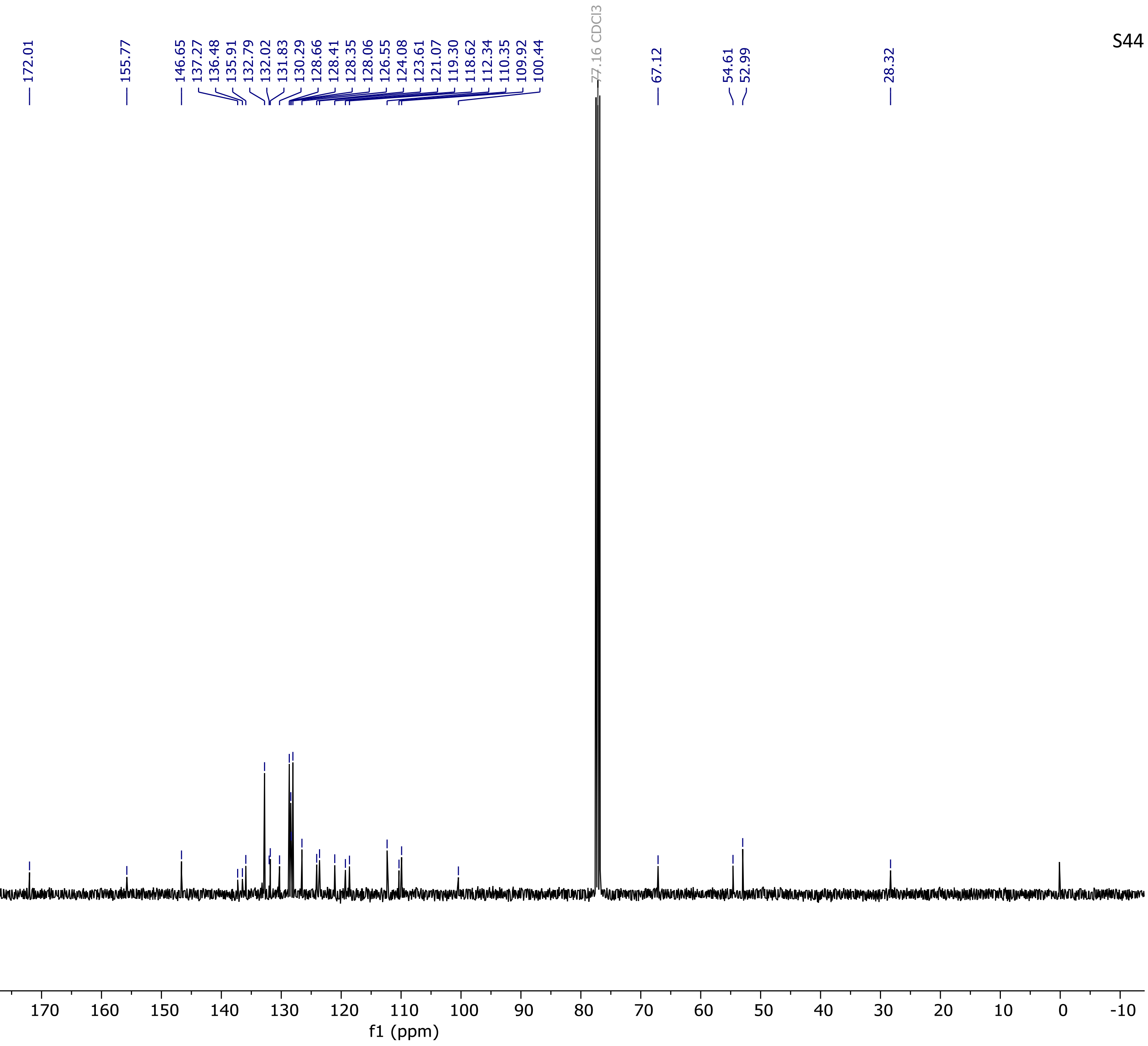

^{1}H NMR (400 MHz, $CDCl_3$)

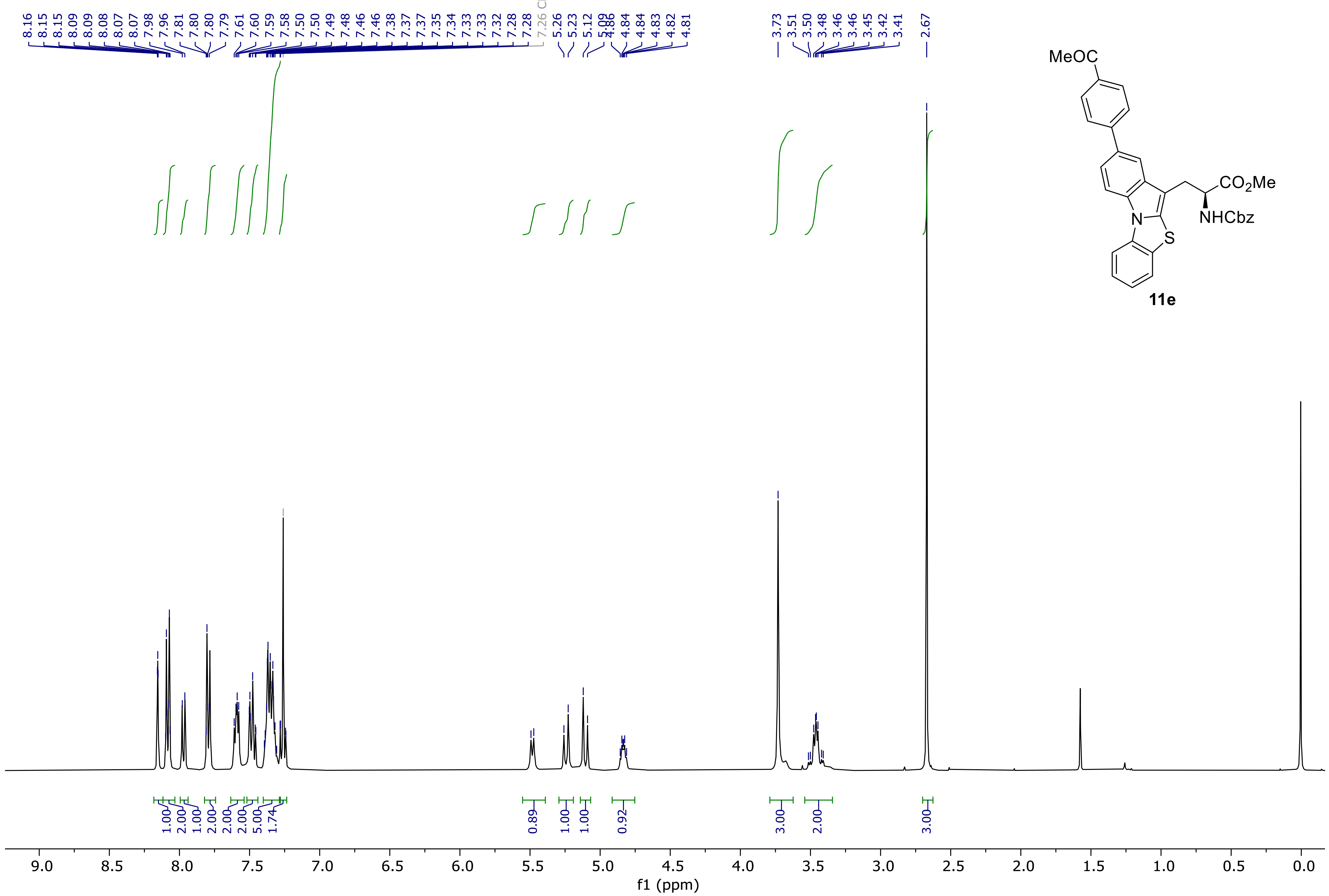

11e

$^{13}C\{^{1}H\}$ NMR (101 MHz, $CDCl_3$)

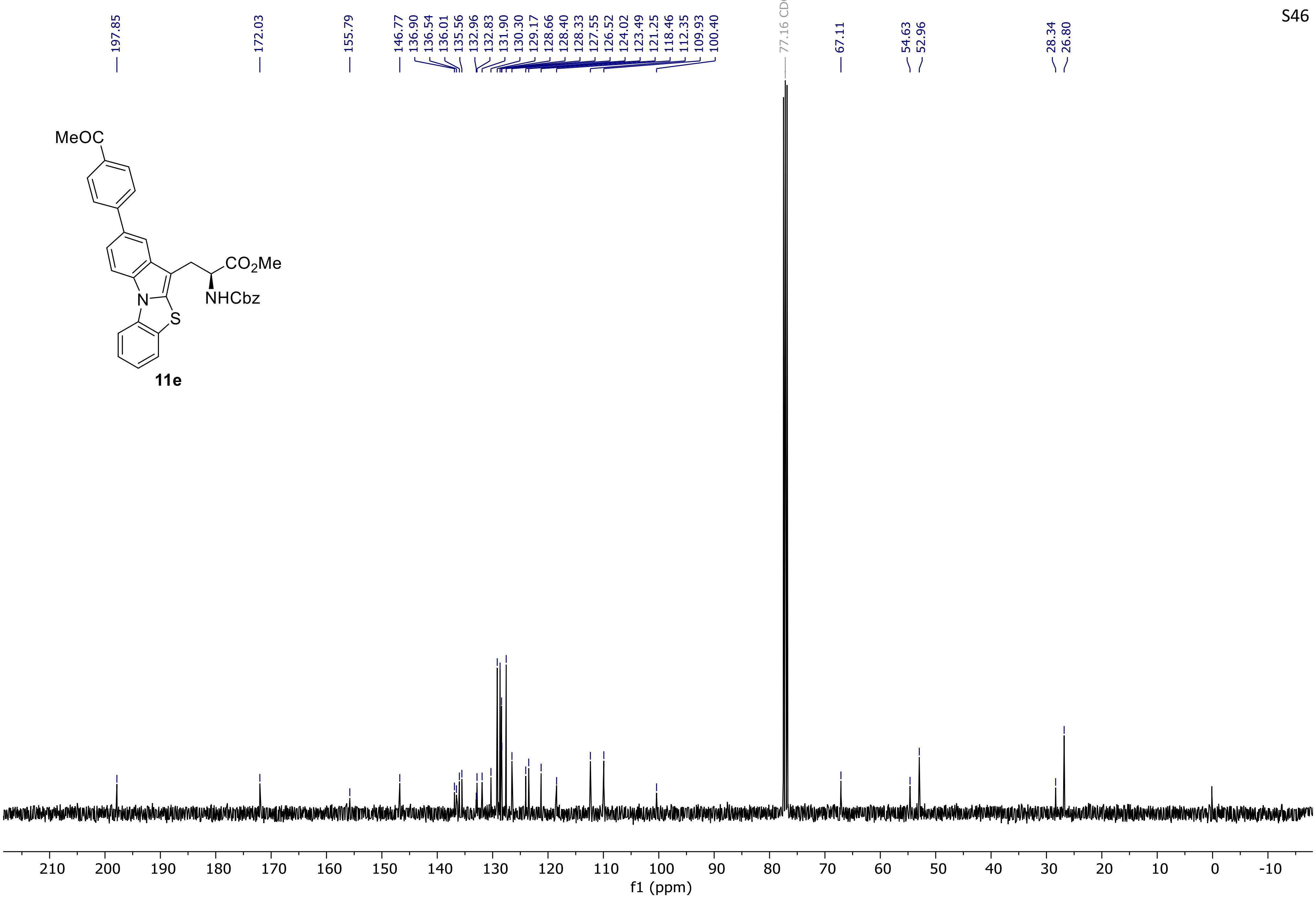

^{1}H NMR (400 MHz, $CDCl_3$)

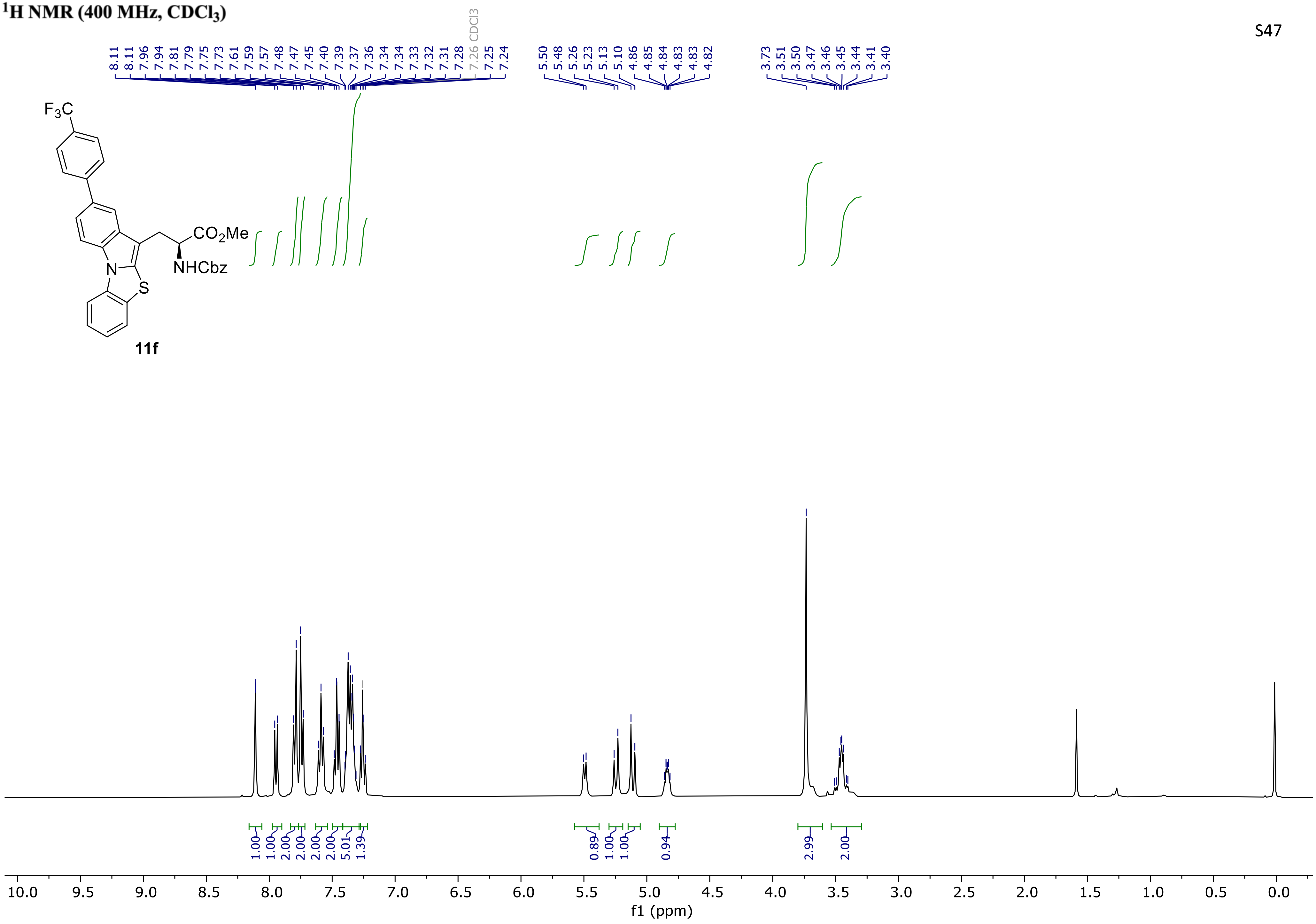

$^{13}C\{^{1}H\}$ NMR (101 MHz, $CDCl_3$)

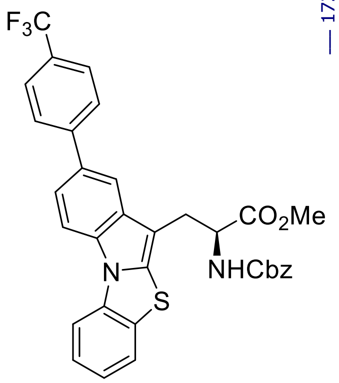

11f

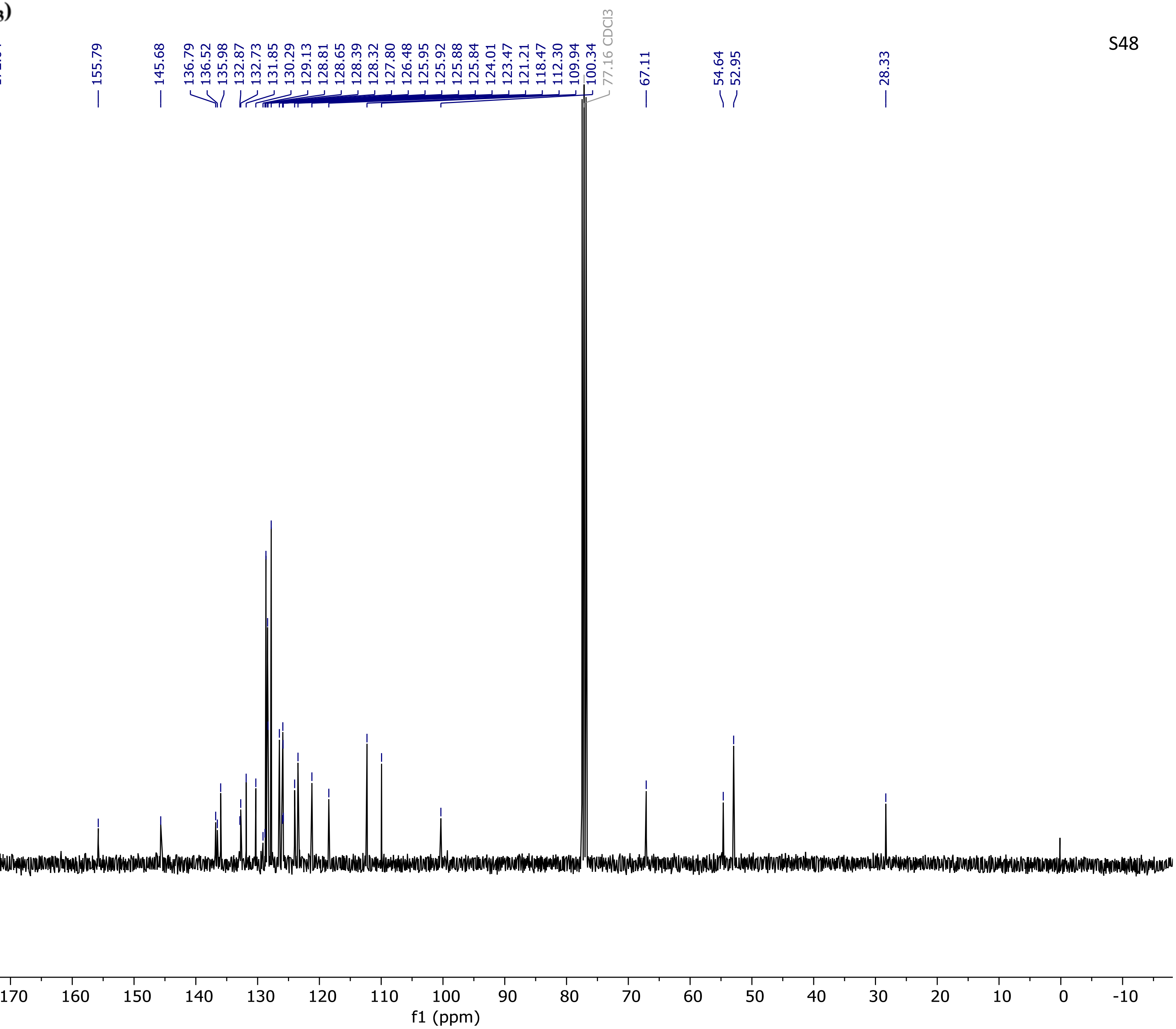

^{1}H NMR (400 MHz, DMSO-d_6)

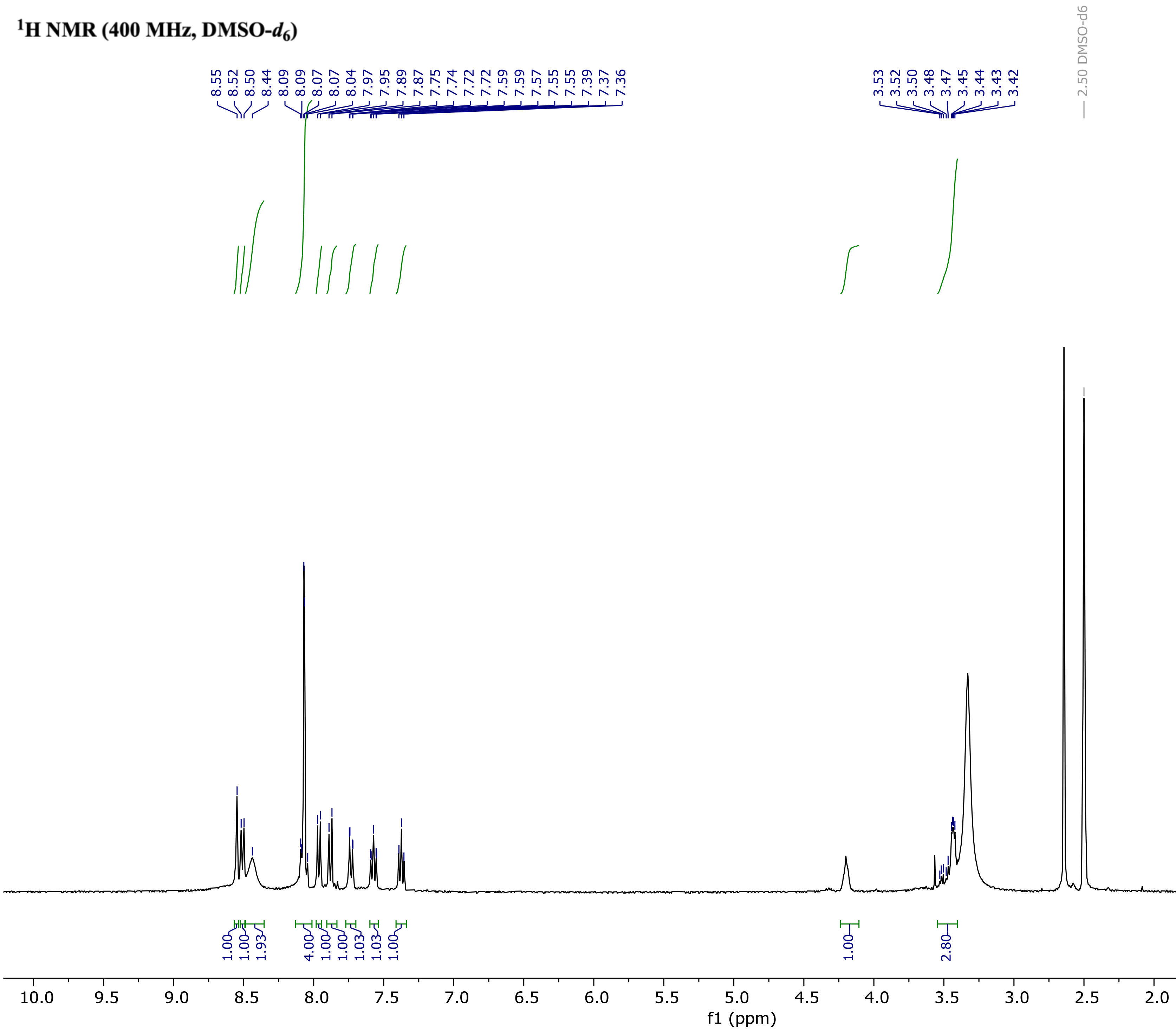

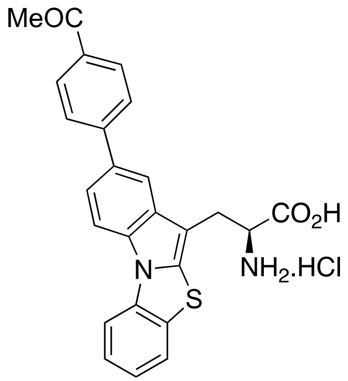

12a

$^{13}C\{^{1}H\}$ NMR (101 MHz, DMSO-d_6)

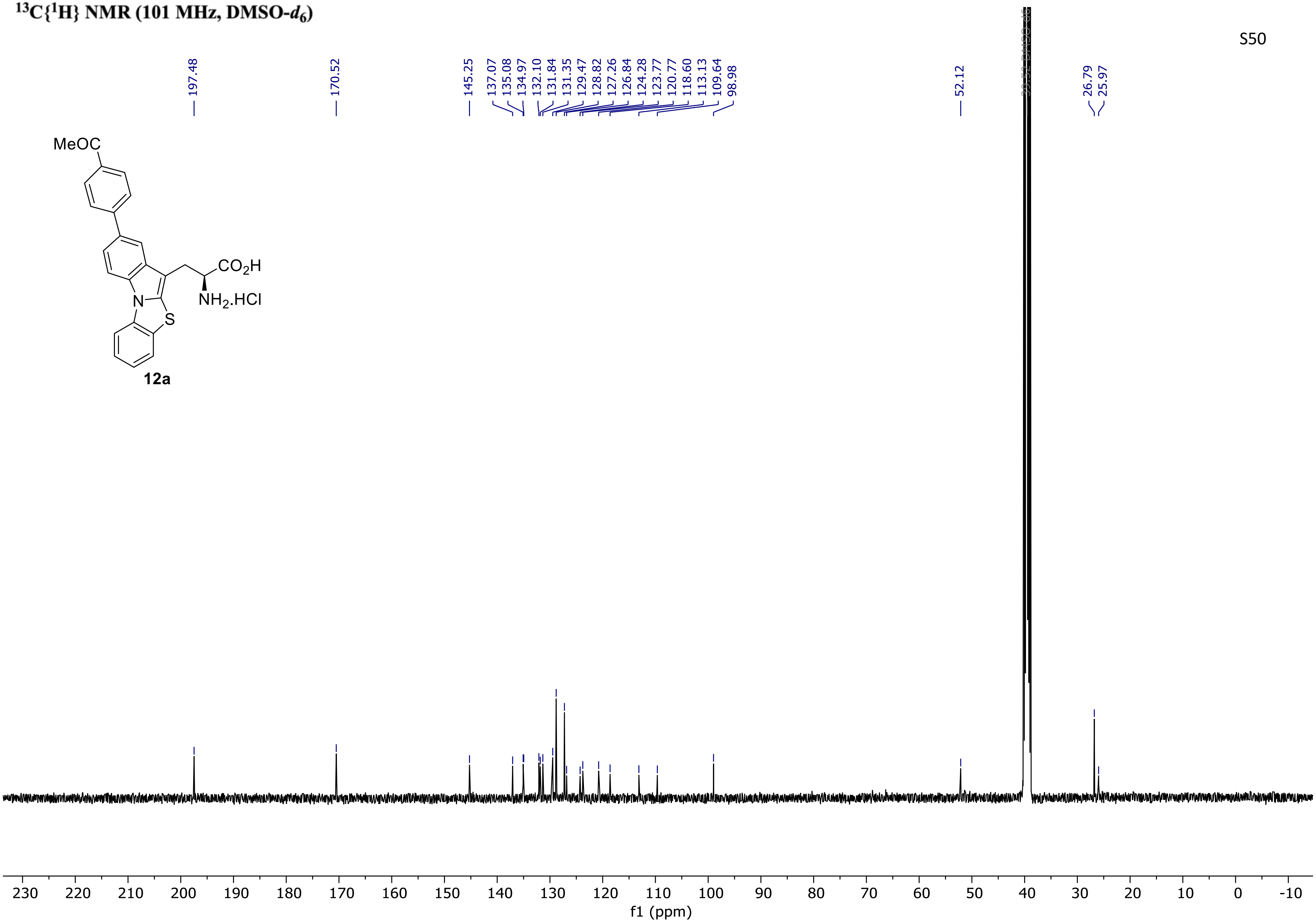

^{1}H NMR (400 MHz, DMSO-d_6)

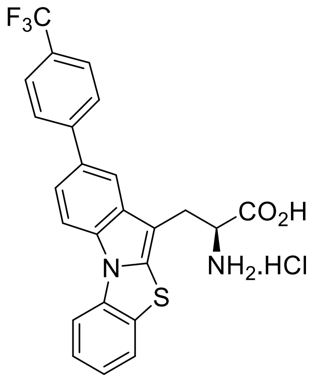

12b

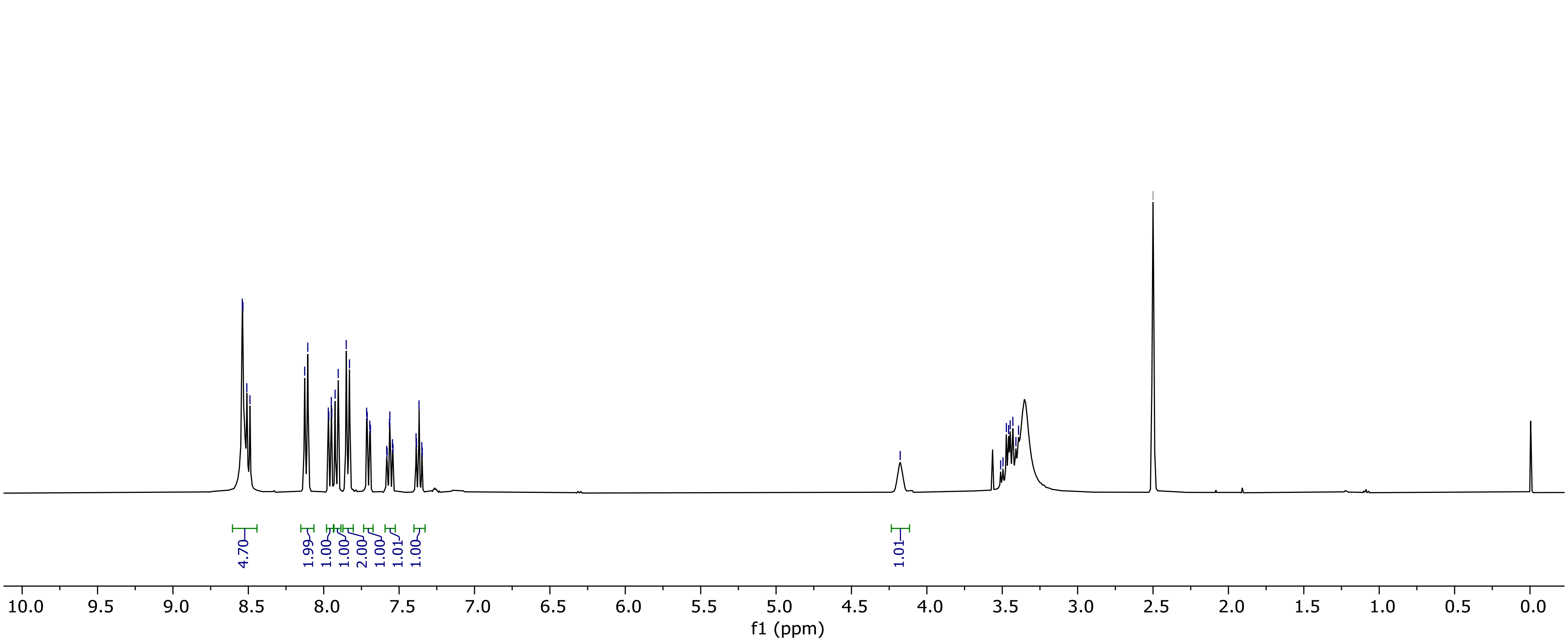

$^{13}C\{^1H\}$ NMR (101 MHz, DMSO-d_6)

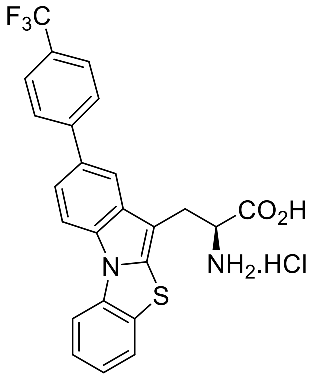

12b

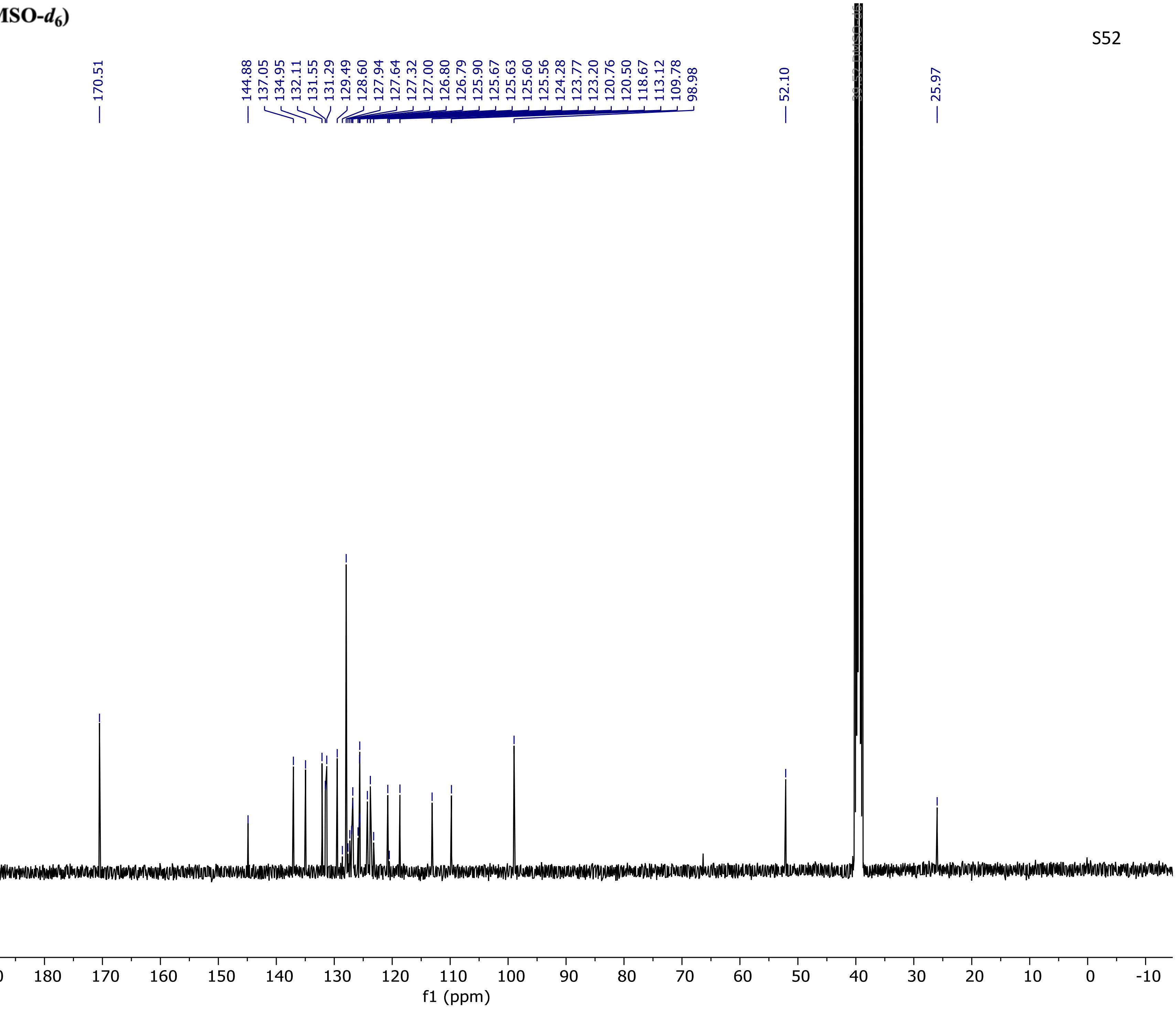

^{1}H NMR (400 MHz, DMSO-d_6)

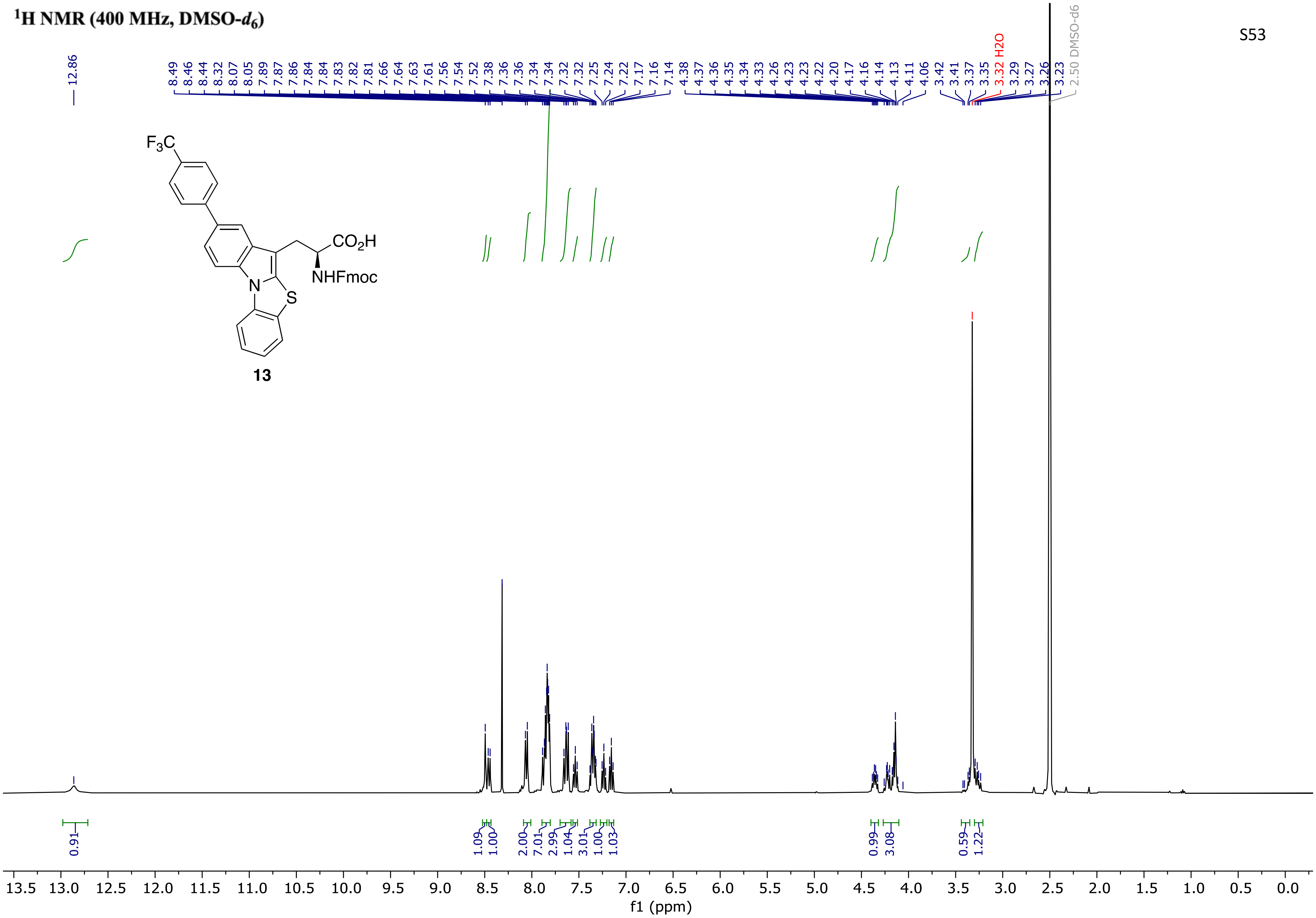

Supplement: Supplementary file 1 — ol3c03851_si_001.pdf [file ol3c03851_si_001.pdf]
